# NOVA1 promotes NSCLC proliferation and invasion by activating Wnt/β-catenin signaling

**DOI:** 10.1186/s12885-022-10164-8

**Published:** 2022-10-25

**Authors:** Lianyue Qu, Yulong Tian, Fan Wang, Zixuan Li

**Affiliations:** 1 Departmentof Pharmacy, The First Hospital of China Medical University, Shenyang, China; 2 Key Laboratory of Diagnostic Imaging and Interventional Radiology of Liaoning Province, The First Hospital of China Medical University, Shenyang, People’s Republic of China

**Keywords:** NOVA1, NSCLC, β-Catenin, Invasion, Proliferation

## Abstract

**Background:**

Neuro-oncological ventral antigen 1 (NOVA1) is a neuron-specific RNA-binding protein which regulates alternative splicing in the developing nervous system. Recent research has found that NOVA1 plays a significant role in carcinogenesis. In this paper, we examine the role of NOVA1 in non-small cell lung cancer (NSCLC) and its underlying molecular mechanisms.

**Methods:**

The expression of NOVA1 in NSCLC was detected by immunohistochemistry and correlations between NOVA1 expression and clinicopathological factors were analyzed by chi–square tests. Kaplan–Meier survival analysis and the Cox regression model were used to evaluate the predictive effect of prognostic factors. Western blotting, Cell Counting Kit-8, colony formation, apoptosis, migration and invasion assays were used to detect the effects of silencing (si)NOVA1 RNA on Wnt/β-catenin signaling and biological behavior in NSCLC cell lines.

**Results:**

Our study showed that expression of NOVA1 was up-regulated and significantly correlated with poor differentiation (*p* = 0.020), advanced TNM stage (*P* = 0.001), T stage (*P* = 0.001) and lymph node metastasis (*P* = 0.000) as well as the expression of β-catenin (*P* = 0.012) in NSCLC. The down-regulation of NSCLC by siRNA significantly inhibited proliferation, migration and invasion and promoted apoptosis in NSCLC cells. Expression of Wnt signaling molecules, including β-catenin, activated β-catenin, cyclin D1, matrix metalloproteinase (MMP)-2 and MMP-7, was also significantly reduced by siNOVA1. The inhibition of Wnt/β-catenin signaling in A549 and H1299 cells by siNOVA1 was reversed after treatment with a β-catenin expression plasmid.

**Conclusion:**

The present study suggests that NOVA1 may serve as a potential prognosis biomarker in NSCLC. High NOVA1 expression was associated with poor survival rate. Finally, in vitro experiments verified that NOVA1 promotes NSCLC cell proliferation and invasion by regulating Wnt/β-catenin signaling.

**Supplementary Information:**

The online version contains supplementary material available at 10.1186/s12885-022-10164-8.

## Background

Lung cancer is one of the cancers with the highest incidence and represents a leading cause of cancer-related deaths worldwide [1]. Although many efforts have been made to study its pathogenesis and treatment, the overall 5-year survival of patients with lung cancer is still low [2]. Following the principles of precision medicine, the development of new biomarker-driven targeted therapies has become more important, and discovery of new biomarkers and therapeutic agents is urgently needed [3].

Neuro-oncological ventral antigen (Nova) was first identified as an antigen in a rare neurological disorder [4]. It functions as a sequence-specific RNA binding protein in the brain and contains a YCAY motif (where Y represents either U or C) [5]. Nova was also the first mammalian tissue-specific splicing factor to be identified [6]. The Nova family consists of NOVA1 and Nova2 subtypes. Previous research revealed that there are ~ 700 NOVA1/Nova2 alternatively spliced exons [7]. NOVA1 is a brain-specific splicing factor predominantly expressed in the ventral spinal cord and midbrain [8]. In addition to its neuronal functions, NOVA1 has other biological roles. Increasing evidence has demonstrated that NOVA1 is involved in numerous pathological processes including cancer. Overexpression of NOVA1 was associated with lower survival rate and increased recurrence in hepatocellular carcinoma patients [9]. Up-regulation of NOVA1 may also play a role in the development of natural killer cell and T-cell lymphomas [10]. It is a candidate biomarker predicting poor prognosis of gastric cancer patients [11]. High levels of NOVA1 were also associated with poor survival outcomes in astrocytoma [12]. Previous research found that NOVA1 knockdown reduces lung cancer cell growth phenotypes [13]. However, the relationship between NOVA1 expression and clinicopathological features of patients and the underlying molecular mechanisms need to be explored.

The Wnt signaling pathway is essential for both normal embryonic development and cell differentiation [14]. β-catenin acts as a transcriptional activator of the Wnt signaling pathway. The dephosphorylated form of β-catenin is activated and contributes to aberrant nuclear overexpression of the pathway [15]. Previous studies revealed that aberrant β-catenin expression was an independent prognostic marker of non-small cell lung cancer (NSCLC) [16]. As a member of a novel family of RNA-binding proteins, we hypothesize that NOVA1 may play a role in regulating the expression and phosphorylation of β-catenin and thereby impact tumor progression of NSCLC.

In this study, we first examined the expression of NOVA1 in NSCLC tissues and analyzed its correlation with clinicopathological factors. Subsequently, we explored the impacts of NOVA1 on cell proliferation, apoptosis, migration and invasion after NOVA1 RNA interference. Finally, we demonstrated that NOVA1 regulates the proliferative and invasive abilities of NSCLC cells by promoting activation of the Wnt/β-catenin signaling pathway.

## Methods

### Immunohistochemistry

Paired cancer and adjacent noncancer paraffin tissue sections used for IHC staining were purchased from Outdo Biotech Co., Ltd. (Shanghai, China). The non-biotin amplification complex method (EliVision™ Super, Maixin, Fuzhou, China) was used for immunohistochemical staining. The sections were deparaffinized in xylene and rehydrated in graded alcohols. Antigen retrieval was performed using heat-mediated antigen retrieval in Tris–EDTA pH 9.0 for 30 min. Endogenous peroxidase activity was blocked using 0.3% H_2_O_2_ for 10 min at room temperature, followed by incubation with normal goat serum to reduce non-specific binding. The sections were incubated with anti-NOVA1 rabbit polyclonal antibody (ab183024), anti-β-catenin(ab32572) (1:150; Abcam, Cambridge, MA, USA) at 4 °C overnight, then allowed to return to room temperature for 30 min. The Elivision™ Super HRP (Mouse/Rabbit) IHC Kit was used. Visualization was performed using DAB-2031 (MaiXin). A semi-quantitative scoring system was used to evaluate NOVA1 staining intensity. Nuclear staining was scored as 0 (no staining), 1 (weak), 2 (moderate) or 3 (strong). Percentage scores for the number of cell nuclei stained were assigned as 1 (1–10%), 2 (10–50%) and 3 (51–100%). The scores from each tumor sample were multiplied to give a final score of 9, and the tumors were categorized based on their scores, with < 3 and ≥ 3, indicating low and high expression, respectively. The percentage scores of β-catenin were assigned as 1(0–25% of tumor cells,2(25–75% of tumor cells)and 3(more than 75% of the tumor cells. The intensity of β-catenin in every single case was categorized 1, 2, or 3 (weak, moderate, or strong). The total immunostaining score from each tumor sample were multiplied to give a final score of 9. This score was subdivided into low expression (< 4),and high expression (≥ 4).

### Cell culture and transfection

The normal human bronchial epithelial cell line HBE and the human lung cancer cell lines H1650 and H827 were maintained in RPMI-1640 medium with 10% fetal bovine serum (FBS; Biological Industries, Kibbutz Beit-Haemek, Israel). A549 and H1299 cell lines were maintained in DMEM/Ham’s F12 medium 1:1 (Biological Industries, Xiaopeng, Shanghai) with 10% FBS. PC12 cell lines were maintained in DMEM medium with 10% FBS. All cells were cultured in a 37 °C incubator with a humidified atmosphere containing 5% CO_2_. The cells were grown in sterile T25 cell culture flasks (Corning, Corning, NY, USA) and were passaged every 2–3 days using 0.25% trypsin (Biological Industries) when 90% cell density was reached. For transfections, cells were seeded in a 6-well plate 24 h before the experiment. The pCMV6 plasmid vector (PS100001), pCMV6-β-catenin plasmid, (RC208947), siRNA negative control (NC) (SR30004) and siNOVA1#1 (SR303213) were all purchased from Origene (Rockville, MD, USA). siNOVA1#2 was synthesized by GenePharma Co. (Shanghai, China). The plasmids or siRNAs were transfected into cells using Lipofectamine 3000 (Invitrogen, Carlsbad, CA, USA), according to the manufacturer’s instructions. After transfection, cells were incubated for 48 h before further testing.

### Western blotting

Western blotting was performed as described previously [17].Total protein from cell lines was extracted and denatured in lysis buffer (Cat. 78,510, Thermo Fisher Scientific, Waltham, MA, USA), then SDS-PAGE protein loading buffer was added. Samples were analyzed using SDS-PAGE and then transferred to a PVDF membrane (Millipore, Billerica, MA, USA). After blocking in 5% non-fat milk, the membranes were incubated overnight at 4 °C. The antibodies used were as follows: NOVA1 (ab183024, 1:1000; Abcam, Cambridge, MA, USA), β-catenin (393,501, 1:100; Santa Cruz Biotechnology, Dallas, TX, USA), active β-catenin (8814) and E-cadherin (3915S, both 1:500; Cell Signaling Technology, Danvers, MA, USA), cyclin B1 (4135S), cyclin D1 (2978S), matrix metalloproteinase (MMP)2 (40994S) and MMP-7 (3801S) all diluted 1:1000; Cell Signaling Technology), and GAPDH (AF7021, 1:3000; Affinity Biosciences, Beijing, China). After primary antibody incubation, the membranes were further incubated with secondary antibody anti-HRP-rabbit IgG (1:5000; Multi Sciences Biotech, Hangzhou, China) at 37 °C for 1 h. Protein bands were visualized with an ECL detection system (Bio-Rad, Hercules, CA, USA. Relative density was quantified using Image Lab™ software (Bio-Rad). In order to reduce the operation error, the blots cut prior to hybridisation with antibodies or prior to chemoluminescence.

The images of replicate blots performed or fuller-length, original, unprocessed blot performed were provided in supplementary material.

### RNA isolation and Real-time PCR

Total RNA was isolated according to the manufacturer’s instructions using TRIzol reagent (Invitrogen). Real-time PCR was performed using SoFast™ EvaGreen® Supermix (Bio-Rad) in a total volume of 20 μl on the Light Cycler®480 II (Roche) as follows: 95 °C for 30 s, followed by 40 cycles of 95 °C for 5 s and 60 °C for 20 s. The primer sequences are listed in Table 1.

**Table 1:** Primer sequences of qRT-PCR

Genes	Primer sequences
NOVA1-F	AGGACCAATACGGGCGAAGACG
NOVA1-R	CACTCGCTCAGTAGTACCTGG
β-catenin-F	AAAGCGGCTGTTAGTCACTGG
β-catenin-R	CGAGTCATTGCATACTGTCCAT
GAPDH-F	ACAACTTTGGTATCGTGGAAGG
GAPDH -R	GCCATCACGCCACAGTTTC

### Cell apoptosis experiments

After transfection with siRNA for 48 h, the A549 and H1299 cells were collected and washed with phosphate-buffered saline twice.

The washed cells were stained using an Annexin V-FITC and PI Apoptosis Detection Kit (Multi Sciences Biotech) according to the manufacturer’s instructions. Apoptosis was analyzed by flow cytometry using Flow Cytometer (BD C6 Plus)and the percentage of cell apoptosis was analyzed using BD Accuri™ software.

### Cell Counting Kit-8 (CCK-8) assays

Cells were plated 24 h post-transfection in 96-well plates. After 48 h of treatment, 10 μl of Cell Counting Kit-8® solution (Dojindo, Kumamoto, Japan) was added and incubated at 37 °C and 5% CO2 for 2 h. The absorbance was taken at 450 nm using a photospectrometer (Thermo Fisher Scientific, Waltham, MA, USA).

### Matrigel invasion assay

We used Matrigel (BD Biosciences, San Jose, CA, USA) and 24-well Transwell chambers with a pore size of 8 mm (Costar, Cambridge, MA, USA) to assess the invasive ability of transfected cells according to the manufacturers’ instructions. DMEM/Ham’s F12 medium (1:1; Biological Industries) in a volume of 700 µl with 15% serum was added to the lower chamber. Cells were trypsinized in 100 µl of serum-free medium and 2 × 10^5^cells were transferred to the upper chamber after transfection for 48 h. The cells located on the lower surfaces of the membrane were fixed with 4% paraformaldehyde and then stained with 0.1% crystal violet (Solarbio, Beijing, China). Cells were counted in ten randomly selected high-power fields under the microscope.

### Colony formation assay

Colony formation assays were performed to examine the biological effect of Nova1 on tumor cell survival. Cells transfected with siNC or siNova1 were plated in six-well plates at a density of 1 × 10^3^ cells/well. Cell colonies were photographed and counted after staining with crystal violet 14 days after plating.

### Wound healing assay

After transfection for 48 h, A549 and H1299 cells were seeded in 6-well plates until confluence reached 90%. A sterile 200-μL pipette tip was used to make a single scratch across the well surface. The cells were then cultured with serum-free medium and the plates were photographed by a digital camera under an inverted microscope (Nikon, Tokyo, Japan) at the same position at 0 and 24 h. Image-Pro Plus 6.0 software (Media Cybernetics, Inc., Silver Springs, MD, USA) was used for quantitative analysis.

### Statistical analysis

All statistical analyses were performed using SPSS 26.0 software (IBM, Armonk, NY, USA). The Chi–square-test was used to examine possible correlations between NOVA1 expression and clinicopathological factors. Kaplan–Meier survival analysis was used to evaluate the prognostic value of NOVA1. A Cox-proportional risk regression model was employed to test the mixed effect of variables for those most closely correlated with the expression levels of NOVA1. Other results were analyzed using Student’s *t*-test. *P* < 0.05 was considered a statistically significant result. Data are shown as the means ± standard deviation (SD).

## Results

### NOVA1 expression was correlated with clinical features and prognosis in NSCLC

**Fig.1:**
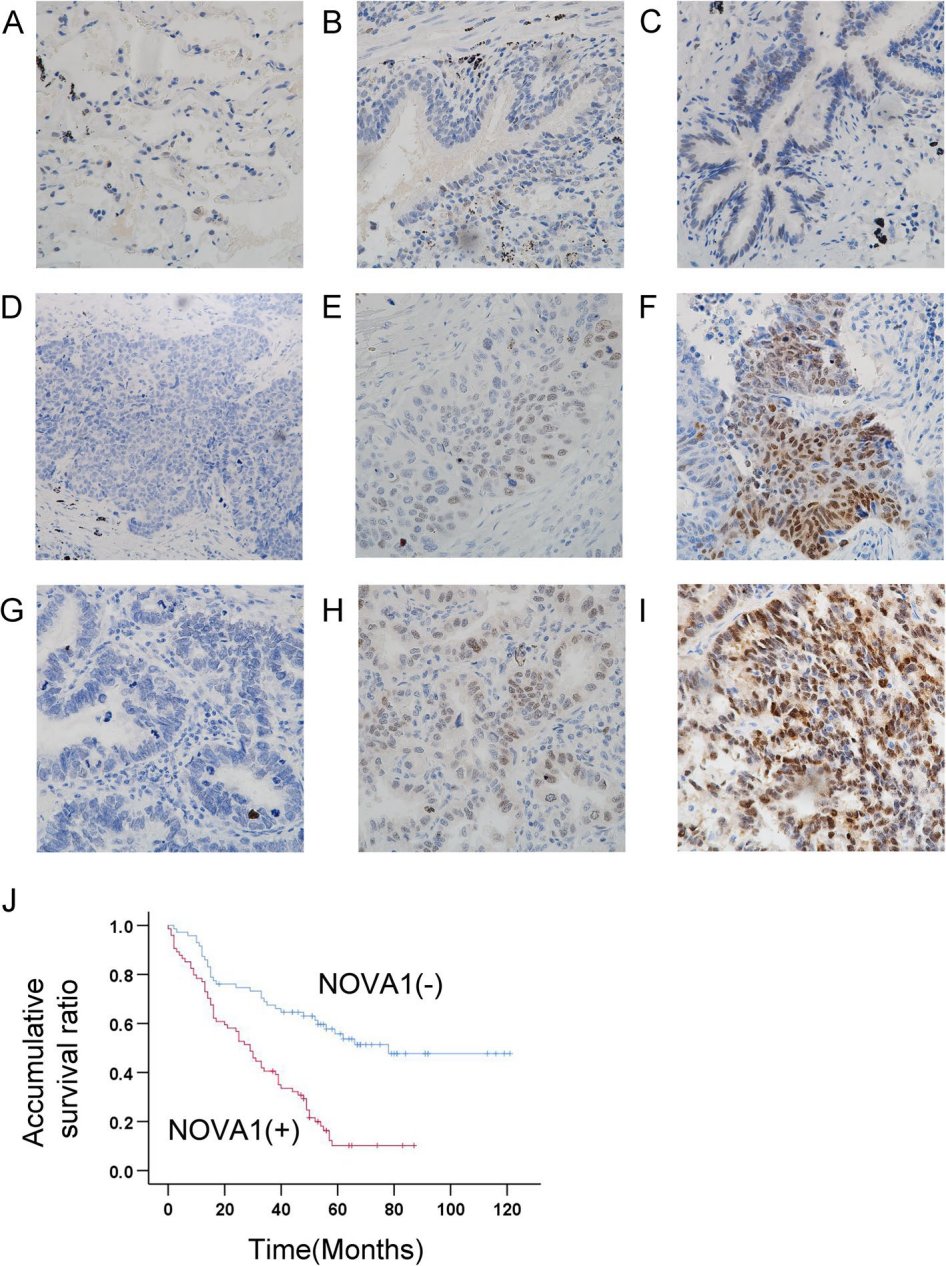
Positive NOVA1 expression correlated with clinical significance and malignant prognosis of NSCLC. **A** Negative expression of NOVA1 in normal alveolar cells. **B** Negative expression of NOVA1 in normal bronchial epithelium. **C** Weak positive expression of NOVA1 in normal bronchial epithelium. **D** Negative expression of NOVA1 in lung squamous cell carcinoma. **E** Weak positive expression of NOVA1 in lung squamous cell carcinoma. **F** Strong positive expression of NOVA1 in lung squamous cell carcinoma. **G** Negative expression of NOVA1 in lung adenocarcinoma. **H** Weak positive expression of NOVA1 in lung adenocarcinoma. **I** Strong positive expression of NOVA1 in lung adenocarcinoma.** J** Kaplan–Meier survival curves of patients with or without NOVA1 expression

We analyzed the expression of NOVA1 in 145 NSCLC specimens and 30 adjacent normal lung tissue specimens. NOVA1 was mainly expressed in the nuclei of cancer cells. In corresponding normal lung tissues, NOVA1 was neither expressed in alveolar cells (Fig. 1A) nor in 21 cases (70.00%) of bronchial epithelial cells (Fig. 1B), and only nine cases (27.00%) of normal bronchial epithelial cells showed some expression of NOVA1 (Fig. 1C). In contrast, high expression of NOVA1 was observed in 51.03% of lung cancer tissues (74/145; Fig. 1D-I).

As listed in Table 2, expression of NOVA1 was not related to age (*p* = 0.597), gender (*p* = 0.154) or histology type of lung cancer (*p* = 0.714). The high expression of NOVA1 correlated significantly with poor differentiation (*p* = 0.020), advanced TNM stage (*P* = 0.001), T stage (*P* = 0.001) and lymph node metastasis (*P* = 0.000) of lung cancer. Analysis of Kaplan–Meier survival curves showed that the median survival time of patients with positive NOVA1 expression (32.862 ± 2.991 months) was significantly shorter than in those without NOVA1 expression (74.737 ± 5.898 months, *P* = 0.000; Fig. 1J).

**Table 2:** Correlations between NOVA1 expression and clinicopathological factors in lung cancers

	N	NOVA1 Negative	NOVA1 Positive	*P*-value
Age				
< 60	48	25 (52.1%))	23 (47.9%))	0.597
≥ 60	97	46 (47.4%)	51 (52.6%)	
Gender				
Male	100	45 (45.0%)	55(55.0%)	0.154
Female	45	26(57.8%)	19(42.2%)	
Histological type				
Adenocarcinoma	90	43(47.8%)	47(52.2%)	0.714
Squamous cell carcinoma	55	28(50.9%)	27(49.1%)	
Differentiation				
Well-moderate	108	59(54.6%)	49(45.4%)	0.020
Poor	37	12(32.4%)	25(67.6%)	
TNM stages				
I-II	93	55(59.1%)	38(40.9%)	0.001
III-IV	52	16(30.8%)	36(69.2%)	
T stage				
T1	24	19(79.2%)	5(20.8%)	0.001
T2-T4	121	52(43.0%)	69(57.0%)	
Lymph node metastasis				
Negative	76	49(64.5%)	27(35.5%)	0.000
Positive	69	22(31.9%)	47(68.1%)	
β-catenin status				
Negative	43	28(65.1%)	15(34.9%)	0.012
Positive	102	43(42.2%)	59(57.8%)	

A total of eight variables including age, gender, histology type, differentiation, TNM stage, T stage, lymph node metastasis and NOVA1 were included in multiple regression analysis. The results demonstrated that advanced TNM stage (*p* = 0.022), high T stage expression (*p* = 0.002) and high NOVA1 expression (*p* = 0.004) were independent predictors of reduced survival (Table 3).

**Table 3:** Multiple regression analysis of the relationship between NOVA1 and lung cancer

Variables	*P* value	OR (odds ratio) value	95%CI for OR	
			Lower	Upper
Age	0.137	1.018	0.994	1.043
Gender	0.235	1.351	0.822	2.219
Histological type	0.703	0.908	0.555	1.488
Differentiation	0.423	0.820	0.505	1.332
TNM stages	0.022	1.959	1.102	3.484
T stage	0.002	4.070	1.691	9.793
Lymph node metastasis	0.875	0.955	0.539	1.693
NOVA1	0.004	2.024	1.252	3.270

**Fig. 2:**
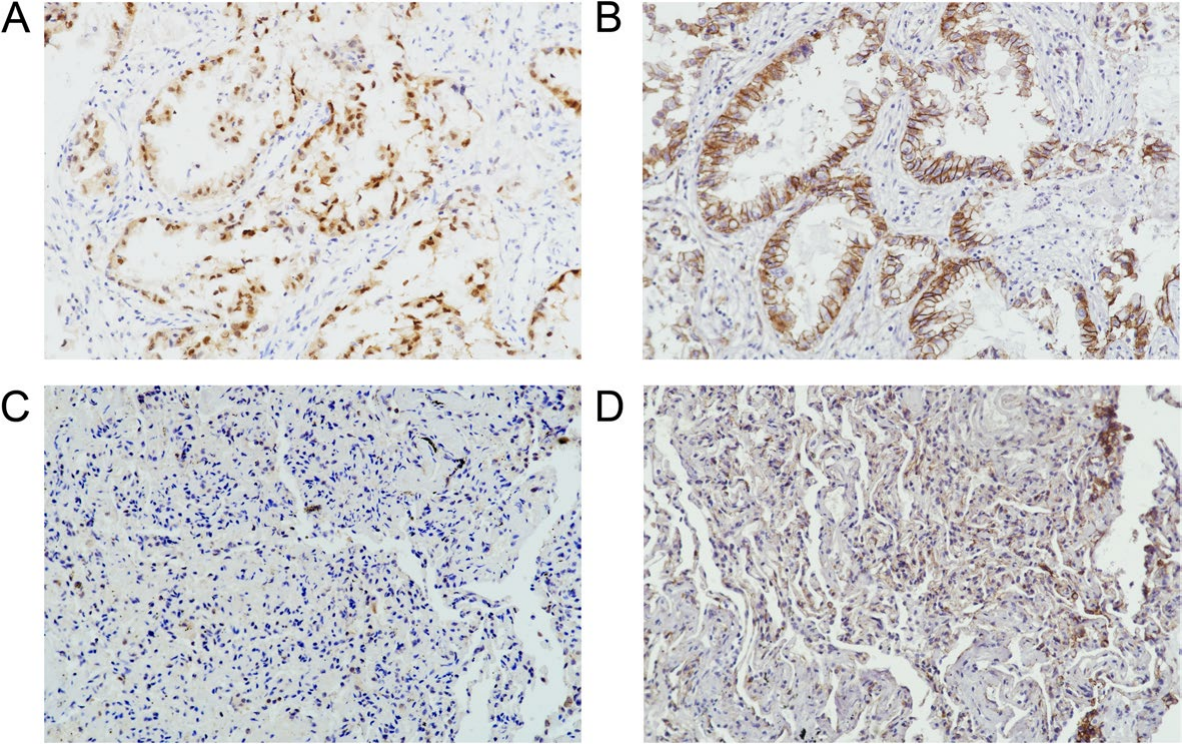
Immunohistochemical staining for NOVA1 and β-catenin in two representative NSCLC samples. **A** Strong expression of NOVA1. **B** Strong expression of β-catenin. **C** Weak expression of NOVA1. **D** Weak expression of β-catenin

We also examined the relation between the NOVA1 protein expression and β-catenin status in NSCLC. We found that β-catenin was expressed in the cell membrane and cytoplasmic. A significant correlation between NOVA1 and β-catenin was observed (*p* = 0.012) (Fig. 2 and Table 2)

### NOVA1 promoted proliferation and inhibited apoptosis in NSCLC cells

**Fig. 3:**
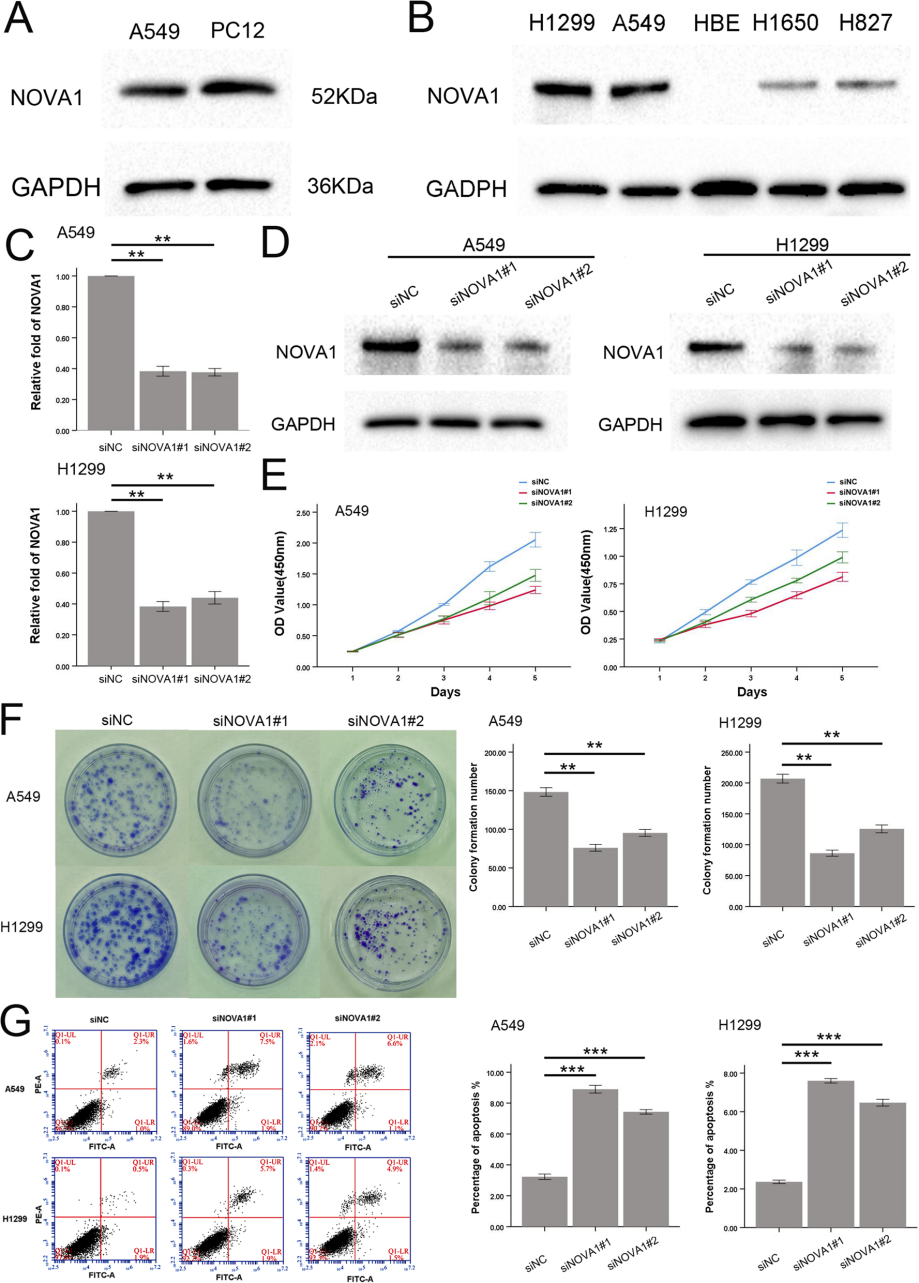
NOVA1 promoted proliferation and inhibited apoptosis in NSCLC cells. **A** The expression of NOVA1 was detected by western blotting in A549 and PC12 cells **B** The expression of NOVA1 was detected by western blotting in human bronchial epithelial cell line (HBE) and NSCLC cell lines (A549, H1299, H1650 and H827). **C** The mRNA levels of NOVA1 in A549 and H1299 cells transfected with siRNA negative control(siNC) and siNOVA1s, **P < 0 .01. **D** The protein levels of NOVA1 in A549 and H1299 cells transfected with siNC and siNOVA1s. **E** Cell viability was detected in A549 and H1299 cells transfected with siRNA negative control(siNC) and siNOVA1s. **F** The images of the colony formation assay in A549 and H1299 cells with or without siNOVA1s.The number of colonies formed by each group is shown in the histogram, **P < 0 .01. **G** The apoptosis of A549 and H1299 cells with or without siNOVA1s were examined by flow cytometer, **P < 0 .01; ***P < 0 .001

We first used the PC12 cell line as a positive control to detect the specificity of NOVA1 antibody (Fig. 3A). We examined the expression of Nova1 in four lung cancer cell lines and in a normal bronchial epithelial cell line (HBE). We found that A549 and H1299 cells showed high Nova1 expression, H1650 and H827 cells showed lower Nova1 expression, and the HBE cell line showed no expression of Nova1 (Fig. 3B). We down-regulated the expression of Nova1 by transfecting Nova1 silencing (si)RNA into A549 and H1299 cells. Compared with the same cells transfected with negative control siRNA (siNC), the siNova1 groups had significantly lower mRNA and protein expression levels of Nova1 (Fig. 3C-D). The results of Cell Counting Kit-8 and colony formation assays revealed that down-regulation of Nova1 produced a remarkable inhibition in the proliferation rate of cancer cells (Fig. 3E-F). Flow cytometry analysis indicated that knockdown of Nova1 also significantly enhanced the apoptosis rate of A549 and H1299 cell lines (Fig. 3G).

### NOVA1 promoted migration and invasion in NSCLC Cells

**Fig. 4:**
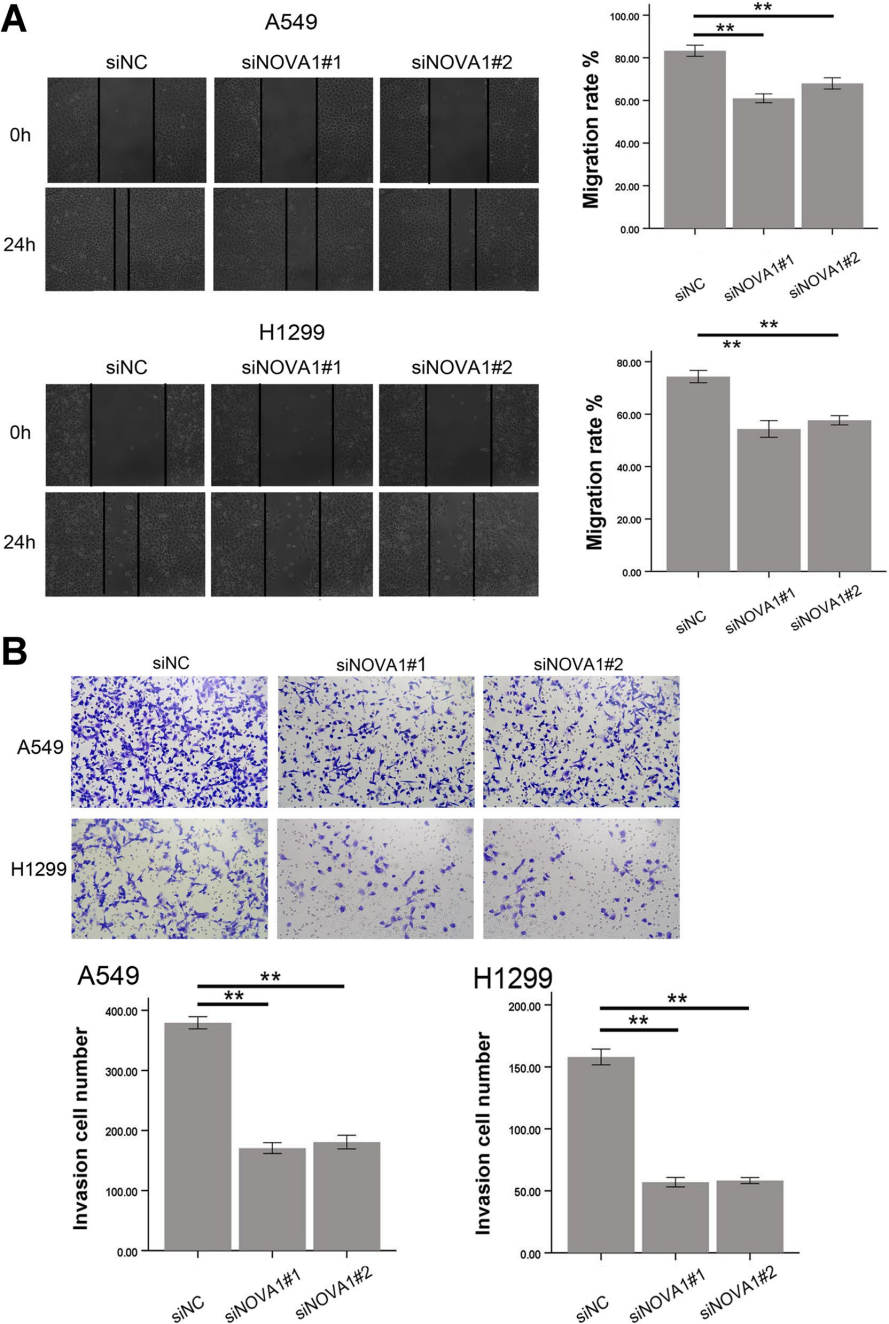
Altered NOVA1 expression modulates the wound healing and invasion of NSCLC cells. **A** A549 and H1299 cells were transfected with or without siNOVA1s and a wound healing assay was undertaken, **P < 0 .01. **B** The invasion of A549 and H1299 cells transfected with or without siNOVA1s, **P < 0 .01

Cell migration ability was detected by wound healing assays. Compared with the controls, NOVA1 siRNA significantly inhibited wound healing of A549 and H1299 cells (Fig. 4A). Similarly, using a Transwell Matrigel assay, we also observed that the knockdown of NOVA1 significantly inhibited cell invasive capacity compared to the NC groups of A549 and H1299 cells (Fig. 4B).

### NOVA1 promoted activation of the Wnt/β-catenin signaling pathway

**Fig. 5:**
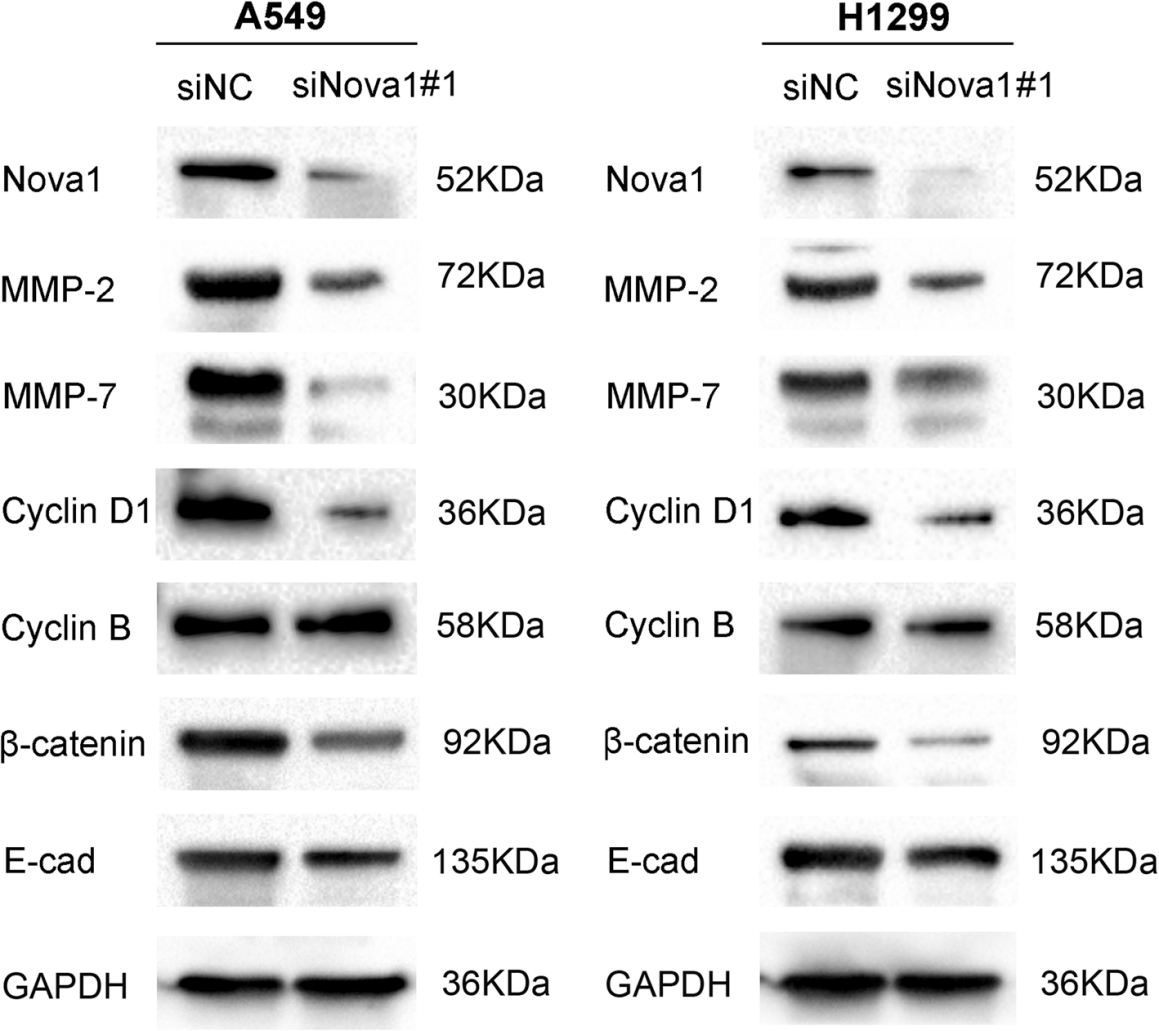
The expression of proteins relevant to Wnt signaling pathway in lung cancer cells after NOVA1 knockdown. Western blotting analysis for NOVA1, MMP-2, MMP-7, Cyclin D1, Cyclin B, β-catenin and E-cadherin in A549 and H1299 cells after transfecting with or without siNOVA1, GAPDH served as an internal control

To elucidate the underlying mechanism by which NOVA1 promoted the proliferation and invasion of NSCLC cells, we investigated the effects of NOVA1 on the expression of key proteins in the Wnt signaling pathway. Western blotting analysis revealed that knockdown of NOVA1 significantly down-regulated the expression of β-catenin, a key molecule in the Wnt pathway. Concomitantly, the expression levels of target genes of the Wnt signaling pathway, such as matrix metalloproteinase (MMP)-2, MMP-7 and cyclin D1 were also significantly reduced (*p* < 0.05). However, the expression levels of cyclin B and E-cadherin were not markedly changed (Fig. 5).

### Overexpression of β-catenin reversed the effect of NOVA1 knockdown in NSCLC cells

**Fig. 6:**
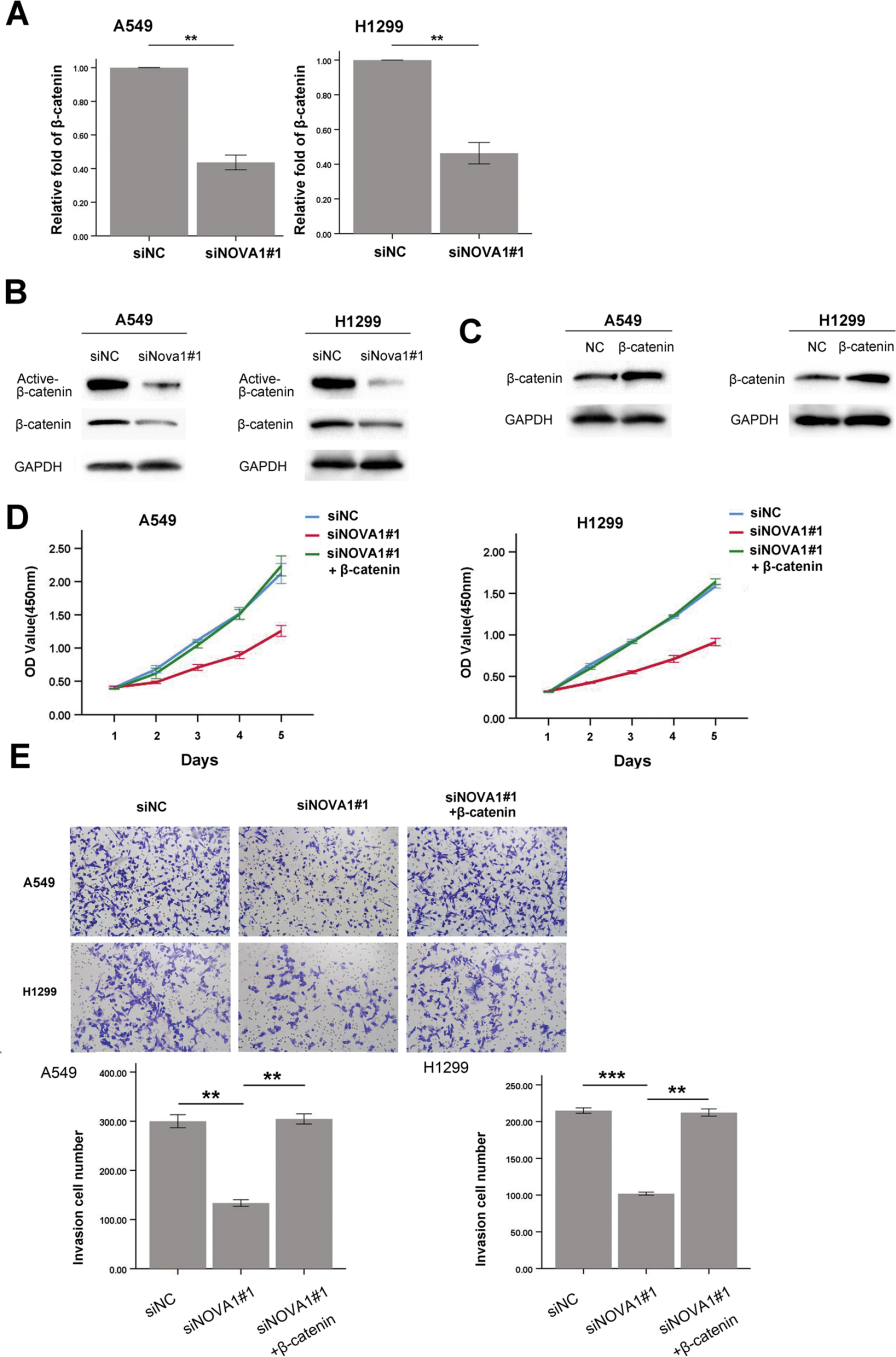
Overexpression of β-catenin can reverse the inhibitory effects of siNOVA1 in lung cancer cells. **A** The mRNA levels of β-catenin in A549 and H1299 cells transfected with siRNA negative siNC and siNOVA1#1, **P < 0 .01. **B** The protein levels of β-catenin and activity of β-catenin in A549 and H1299 cells transfected with siNC and siNOVA1#1. **C** The protein levels of β-catenin in A549 and H1299 cells transfected with β-catenin plasmid. **D** The growth curves of A549 and H1299 cells transfected with siNC, siNOVA1#1 and siNOVA1 + β-catenin plasmid. **E** The invasion of A549 and H1299 cells transfected with siNC, siNOVA1#1 and siNOVA1 + β-catenin plasmid, **P < 0 .01; ***P < 0 .001

To further confirm whether NOVA1 regulation of cell proliferation and invasion were mediated via the activity of β-catenin, we again knocked down NOVA1 and found that the mRNA level of β-catenin was down-regulated after 48 h transfection with NOVA1 siRNA (Fig. 6A). Also attenuated were activated β-catenin protein levels and total β-catenin protein (Fig. 6B). Finally, we employed a β-catenin expression plasmid to evaluate the molecular effects of NOVA1 (Fig. 6C). Knockdown of NOVA1 inhibited proliferation; however, cotransfection with siNOVA1 and β-catenin plasmids restored proliferative ability (Fig. 6D). Matrigel invasion assays also showed that the inhibitory effects of siNOVA1 on invasive capacity were reversed by the β-catenin plasmid (Fig. 6E).

## Discussion

NOVA1 is a well-defined splicing factor responsible for synapse formation [18], which regulates alternative splicing in the developing nervous system [19]. Altered NOVA1 splicing activity is associated with neurological disorders as well as cancer biology [9, 20]. Reports of NOVA1 in NSCLC have been rare [13]; therefore, investigating the functions and underlying mechanisms of NOVA1 in NSCLC may help to provide novel therapeutic targets.

Previous studies have found that high expression of Nova1 is closely associated with poor survival in small cell lung cancer patients and has served as a promising predictive factor for prognosis in this disease [21]. In the present study, we found that Nova 1 is expressed in the nucleus, which is consistent with previous studies of astrocytoma, head and neck squamous cell carcinoma [12, 22], but is not consistent with studies in liver cancer and melanoma [9, 23], in which NOVA1 was only detected in the cytoplasm of liver cancer and melanoma cells. This may mean that NOVA1 has different mechanisms in diverse types of cancer.

We also demonstrated that the expression of NOVA1 was significantly increased in NSCLC tissues compared to normal lung tissues, and that it was correlated with the poor differentiation, TNM stage, T stage and lymph node metastasis of NSCLC. Through univariate analysis, we found that NSCLC patients with high expression of NOVA1 had shorter survival times compared to patients with low expression. COX regression analysis of intratumoral NOVA1 found that NOVA1 was an independent prognostic factor for NSCLC. In addition, we also found that advanced TNM stage and T stage were both strong risk factors for shorter survival time of lung cancer patients. The survival analysis in the present study is consistent with previous studies of SCLC as well as hepatocellular carcinoma and colorectal cancer, which identified NOVA1 expression as associated with unfavorable clinical outcome in both diseases [9, 21, 24]. To the best of our knowledge, this is the first report concerning the expression pattern and clinical significance of NOVA1 in NSCLC.

Next, we investigated the functional role of NOVA1 in NSCLC cell lines. First, our data showed that NOVA1 was highly expressed in four NSCLC cell lines, but no expression of NOVA1 was detected in HBE cells. By transfecting with NOVA1 siRNA, we found that NOVA1 knockdown suppressed the proliferation, migration and invasion of NSCLC cells and promoted their apoptosis.

Recently, NOVA1 had been reported to serve as downstream target gene for a series of microRNAs and was demonstrated to be responsible for the regulation of cellular biological behavior in cancer [25–28]. The inhibitory effect of microRNA miR-193a-5p on the PTEN/PI3k/AKT pathway can be abrogated by NOVA1 in glioblastoma [29]. In PC12 cells, NOVA1 was linked to resistance to hypoxia-induced apoptosis via the Bax/Bcl-2/caspase-3 pathway [30]. NOVA1 enhanced IL-6/JAK2/STAT3 signaling in turn to up-regulate MMPs in colorectal cancer [24]. However, the pathway underlying the effect of NOVA1 in lung cancer requires further study.

Wnt/β-catenin signaling is critical in NSCLC, which substantially impacts NSCLC tumorigenesis, prognosis and resistance to therapy [31]. In the present study, we showed that down-regulation of NOVA1 reduced the expression and activation of β-catenin, as well as the expression of Wnt target genes such as cyclin D1, MMP-2 and MMP-7. β-catenin is a pivotal target molecule in the Wnt pathway. The active form of β-catenin is dephosphorylated β-catenin, which can translocate to the nucleus and stimulate the expression of Wnt target molecules such as cyclin D1 and MMP-7. β-catenin-positive patients have significantly shorter survivals than β-catenin-negative patients in NSCLC [16, 32]. Overexpression of cyclin D1 has been observed in NSCLC and was shown to be a key driver of malignant transformation [33]. MMP-2 and MMP-7 belong to the family of matrix metalloproteinases which plays crucial roles in NSCLC by degrading various protein components of the extracellular matrix [34]. Thus, NOVA1 acts as an oncogene in lung cancers by activating the Wnt signaling pathway.

Expression of NOVA1 in lung cancer cells shifts human telomerase (hTERT) splicing [13].It has been demonstrated that NOVA1 is a β-catenin RNA-binding protein that enhances the stability of β-catenin mRNA in breast cancer cells [35]. In our study, we found that down-regulation of NOVA1 reduced the expression of β-catenin mRNA. The expression of β-catenin and activity of β-catenin were both reduced, which indicates that NOVA1 may decrease the activity of β-catenin through a pre-transcriptional mechanism.

To confirm the relationship between NOVA1 and β-catenin, we employed a β-catenin expression plasmid. We found siNOVA1 inhibited the proliferation rate and invasive capacity of lung cancer cells, which was reversed by the β-catenin plasmid. These results further confirm the correlation between NOVA1 and β-catenin in tissue. Therefore, NOVA1 may regulate the biological behavior of NSCLC by activating the Wnt/β-catenin signaling pathway. 

This study has some limitations that should be mentioned. There were no experiments to examine all of the upstream and downstream proteins associated with cell proliferation and invasion, as well as the signaling pathways associated with cell apoptosis and migration. Furthermore, *in vivo* xenograft tumor experiments should be utilized to verify the relationship between NOVA1 expression and cell biological behavior.

## Conclusions

Taken together, our findings indicate that Nova1 is a novel oncogene which is commonly up-regulated in NSCLC. High Nova1 expression is associated with a poor survival rate. *In vitro* experiments demonstrated that NOVA1 promotes NSCLC cell proliferation and invasion. Our study also suggested that the Wnt/β-catenin signaling axis may play a crucial role in regulating NOVA1-induced malignant behavior of NSCLC cells. These studies thus provide a new perspective on the role of NOVA1 proteins in cancer initiation and progression and shed light on the potential diagnostic value of NOVA1 in NSCLC.

## Supplementary Information

**Additional file 1: Supplementary material.** Original images of WB-1. Original images of WB-2.

## Data Availability

The datasets used and/or analyzed during the current study are available from the corresponding author on reasonable request.

## References

[1] Thai AA, Solomon BJ, Sequist LV, Gainor JF, Heist RS (2021). Lung cancer. Lancet.

[2] Lu T, Yang X, Huang Y, Zhao M, Li M, Ma K (2019). Trends in the incidence, treatment, and survival of patients with lung cancer in the last four decades. Cancer Manag Res.

[3] Kerr KM, Bibeau F, Thunnissen E, Botling J, Ryska A, Wolf J (2021). The evolving landscape of biomarker testing for non-small cell lung cancer in Europe. Lung Cancer.

[4] Buckanovich RJ, Posner JB, Darnell RB (1993). Nova, the paraneoplastic Ri antigen, is homologous to an RNA-binding protein and is specifically expressed in the developing motor system. Neuron.

[5] Ule J, Jensen KB, Ruggiu M, Mele A, Ule A, Darnell RB (2003). CLIP identifies Nova-regulated RNA networks in the brain. Science.

[6] Dredge BK, Darnell RB (2003). Nova regulates GABA(A) receptor gamma2 alternative splicing via a distal downstream UCAU-rich intronic splicing enhancer. Mol Cell Biol.

[7] Saito Y, Miranda-Rottmann S, Ruggiu M, Park CY, Fak JJ, Zhong R (2016). NOVA2-mediated RNA regulation is required for axonal pathfinding during development. Elife.

[8] Graus F, Rowe G, Fueyo J, Darnell RB, Dalmau J (1993). The neuronal nuclear antigen recognized by the human anti-Ri autoantibody is expressed in central but not peripheral nervous system neurons. Neurosci Lett.

[9] Zhang YA, Zhu JM, Yin J, Tang WQ, Guo YM, Shen XZ (2014). High expression of neuro-oncological ventral antigen 1 correlates with poor prognosis in hepatocellular carcinoma. PLoS ONE.

[10] Kim EK, Yoon SO, Kim SH, Yang WI, Cho YA, Kim SJ (2016). Upregulated Neuro-oncological Ventral Antigen 1 (NOVA1) expression is specific to mature and immature T- and NK-Cell lymphomas. J Pathol Transl Med.

[11] Yoon SO, Kim EK, Lee M, Jung WY, Lee H, Kang Y (2016). NOVA1 inhibition by miR-146b-5p in the remnant tissue microenvironment defines occult residual disease after gastric cancer removal. Oncotarget.

[12] Gimenez M, Marie SK, Oba-Shinjo S, Uno M, Izumi C, Oliveira JB (2015). Quantitative proteomic analysis shows differentially expressed HSPB1 in glioblastoma as a discriminating short from long survival factor and NOVA1 as a differentiation factor between low-grade astrocytoma and oligodendroglioma. BMC Cancer.

[13] Ludlow AT, Wong MS, Robin JD, Batten K, Yuan L, Lai TP (2018). NOVA1 regulates hTERT splicing and cell growth in non-small cell lung cancer. Nat Commun.

[14] Wen X, Wu Y, Awadasseid A, Tanaka Y, Zhang W (2020). New advances in canonical Wnt/beta-catenin signaling in cancer. Cancer Manag Res.

[15] Kolegova ES, Shashova EE, Kostromitskii DN, Dobrodeev AY, Kondakova IV (2020). Beta-catenin in non-small cells lung cancer and its association with proteasomes. Bull Exp Biol Med.

[16] Xu X, Sun PL, Li JZ, Jheon S, Lee CT, Chung JH (2011). Aberrant Wnt1/beta-catenin expression is an independent poor prognostic marker of non-small cell lung cancer after surgery. J Thorac Oncol.

[17] Qu L, Tian Y, Hong D, Wang F, Li Z (2022). Mig-6 inhibits autophagy in HCC cell lines by modulating miR-193a-3p. Int J Med Sci.

[18] Ule J, Ule A, Spencer J, Williams A, Hu JS, Cline M (2005). Nova regulates brain-specific splicing to shape the synapse. Nat Genet.

[19] Jensen KB, Dredge BK, Stefani G, Zhong R, Buckanovich RJ, Okano HJ (2000). Nova-1 regulates neuron-specific alternative splicing and is essential for neuronal viability. Neuron.

[20] Shen B, Zhang Y, Yu S, Yuan Y, Zhong Y, Lu J (2015). MicroRNA-339, an epigenetic modulating target is involved in human gastric carcinogenesis through targeting NOVA1. FEBS Lett.

[21] Liu M, Deng S, Xiao T, Gao J (2020). NOVA1 expression is associated with clinicopathological characteristics and prognosis in patients with small cell lung cancer. Transl Cancer Res.

[22] Kim EK, Cho YA, Seo MK, Ryu H, Cho BC, Koh YW (2019). NOVA1 induction by inflammation and NOVA1 suppression by epigenetic regulation in head and neck squamous cell carcinoma. Sci Rep.

[23] Yu X, Zheng H, Chan MTV, Wu WKK (2018). NOVA1 acts as an oncogene in melanoma via regulating FOXO3a expression. J Cell Mol Med.

[24] Hong YG, Xu GS, Yu GY, Zhou JD, Liu QZ, Ni JS (2019). The RNA binding protein neuro-oncological ventral antigen 1 (NOVA1) regulates IL-6 mRNA stability to enhance JAK2-STAT3 signaling in CRC. Surg Oncol.

[25] Sun S, Wang R, Yi S, Li S, Wang L, Wang J (2021). Roles of the microRNA3383p/NOVA1 axis in retinoblastoma. Mol Med Rep.

[26] Cao Y, Zhang F, Wang H, Bi C, Cui J, Liu F (2021). LncRNA MALAT1 mediates doxorubicin resistance of hepatocellular carcinoma by regulating miR-3129-5p/Nova1 axis. Mol Cell Biochem.

[27] Ye CY, Zheng CP, Zhou WJ, Weng SS (2020). MiR-582–5p inhibits the growth and invasion of osteosarcoma cell by targeting NOVA1. Eur Rev Med Pharmacol Sci.

[28] Luo Y, Hao T, Zhang J, Zhang M, Sun P, Wu L (2019). MicroRNA-592 suppresses the malignant phenotypes of thyroid cancer by regulating lncRNA NEAT1 and downregulating NOVA1. Int J Mol Med.

[29] Jin L, Li H, Wang J, Lin D, Yin K, Lin L (2019). MicroRNA-193a-5p exerts a tumor suppressor role in glioblastoma via modulating NOVA1. J Cell Biochem.

[30] Li H, Lv B, Kong L, Xia J, Zhu M, Hu L (2017). Nova1 mediates resistance of rat pheochromocytoma cells to hypoxia-induced apoptosis via the Bax/Bcl-2/caspase-3 pathway. Int J Mol Med.

[31] Stewart DJ (2014). Wnt signaling pathway in non-small cell lung cancer. J Natl Cancer Inst.

[32] Huang CL, Liu D, Ishikawa S, Nakashima T, Nakashima N, Yokomise H (2008). Wnt1 overexpression promotes tumour progression in non-small cell lung cancer. Eur J Cancer.

[33] Gautschi O, Ratschiller D, Gugger M, Betticher DC, Heighway J (2007). Cyclin D1 in non-small cell lung cancer: a key driver of malignant transformation. Lung Cancer.

[34] Jablonska-Trypuc A, Matejczyk M, Rosochacki S (2016). Matrix metalloproteinases (MMPs), the main extracellular matrix (ECM) enzymes in collagen degradation, as a target for anticancer drugs. J Enzyme Inhib Med Chem.

[35] Tang S, Zhao Y, He X, Zhu J, Chen S, Wen J (2020). Identification of NOVA family proteins as novel beta-catenin RNA-binding proteins that promote epithelial-mesenchymal transition. RNA Biol.

