# Supplementary material for: NOVA1 promotes NSCLC proliferation and invasion by activating Wnt/β-catenin signaling
Source: BMC Cancer. 2022 Oct 25;22:1091. doi: 10.1186/s12885-022-10164-8 (PMC9594932; doi:10.1186/s12885-022-10164-8)

Figure 2-A


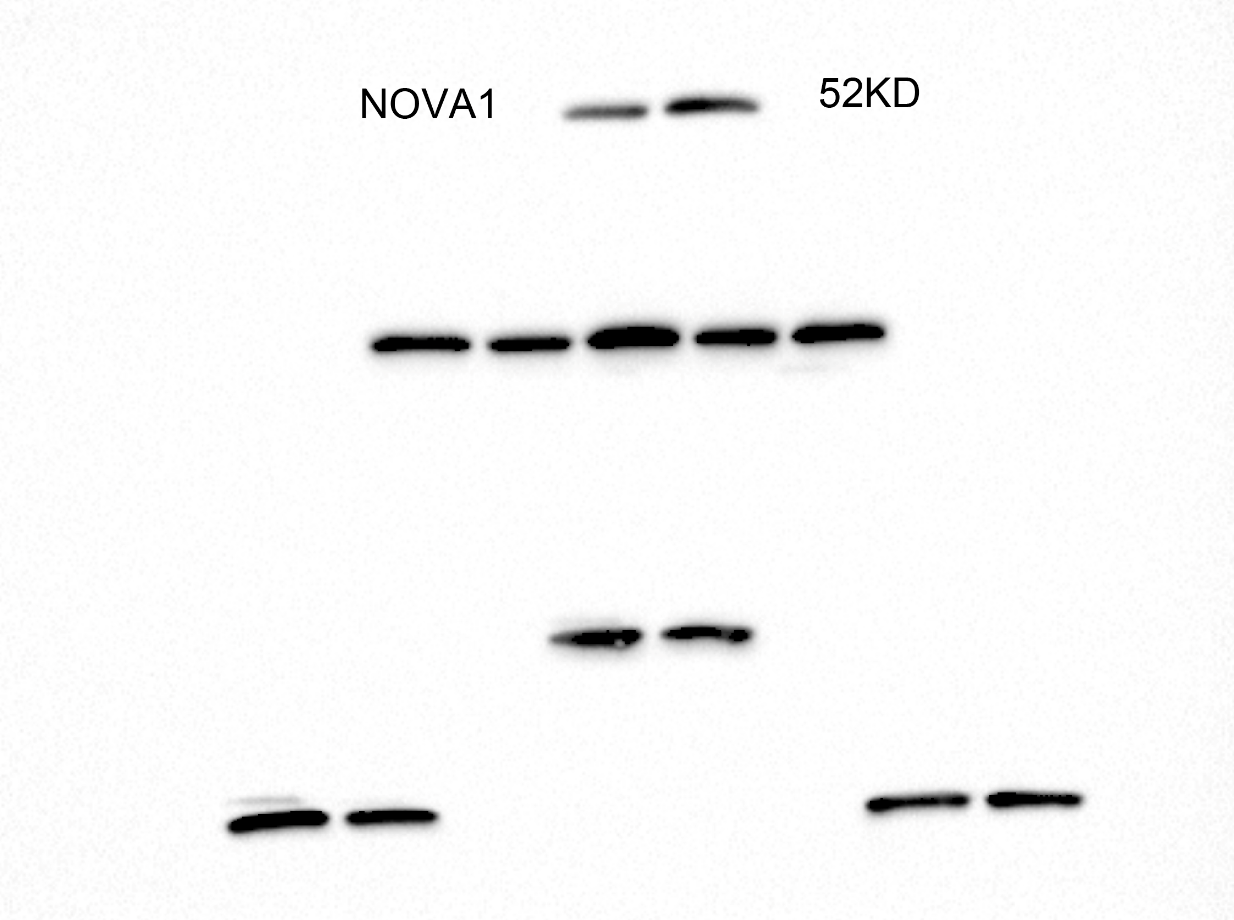


NOVA1

GADPH


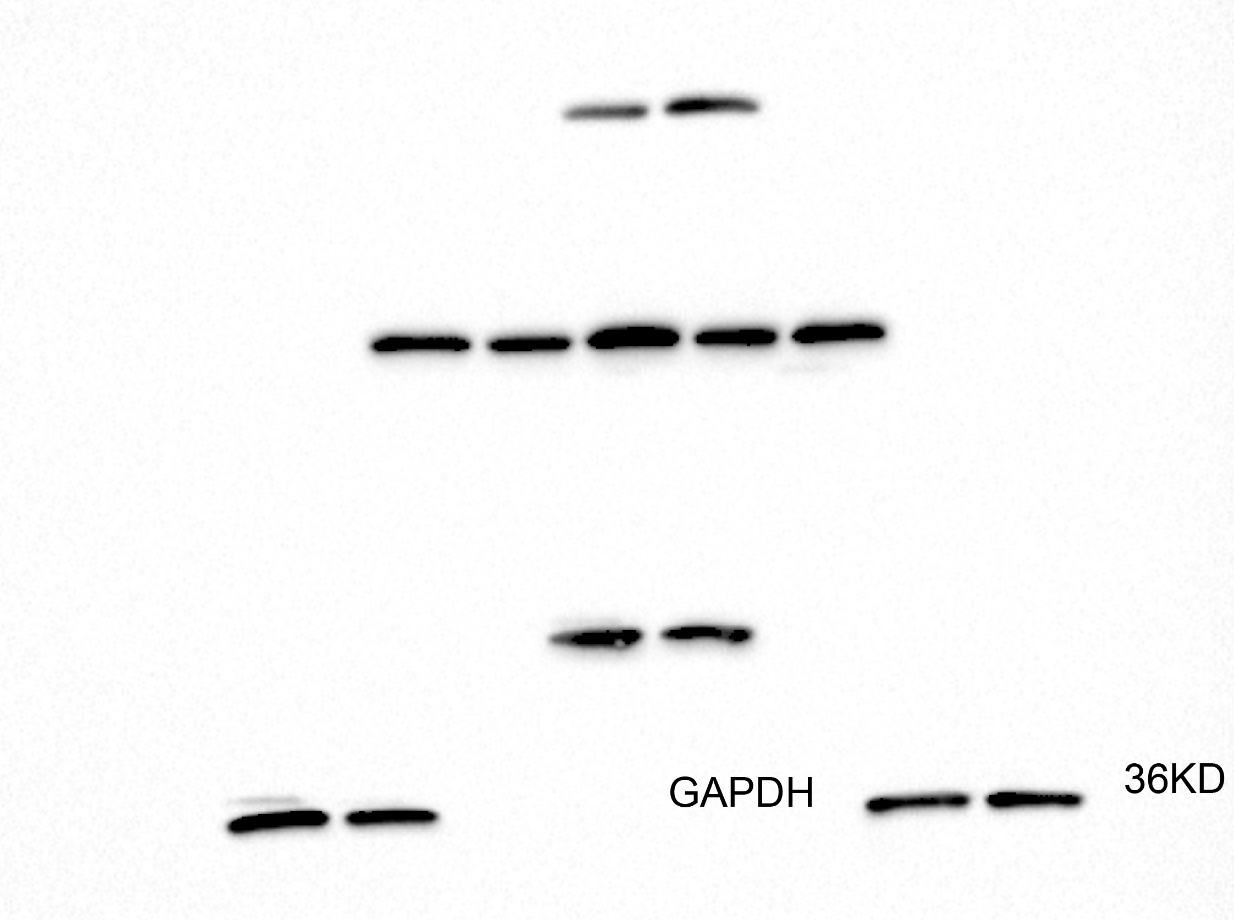


Figure 2-B

NOVA1


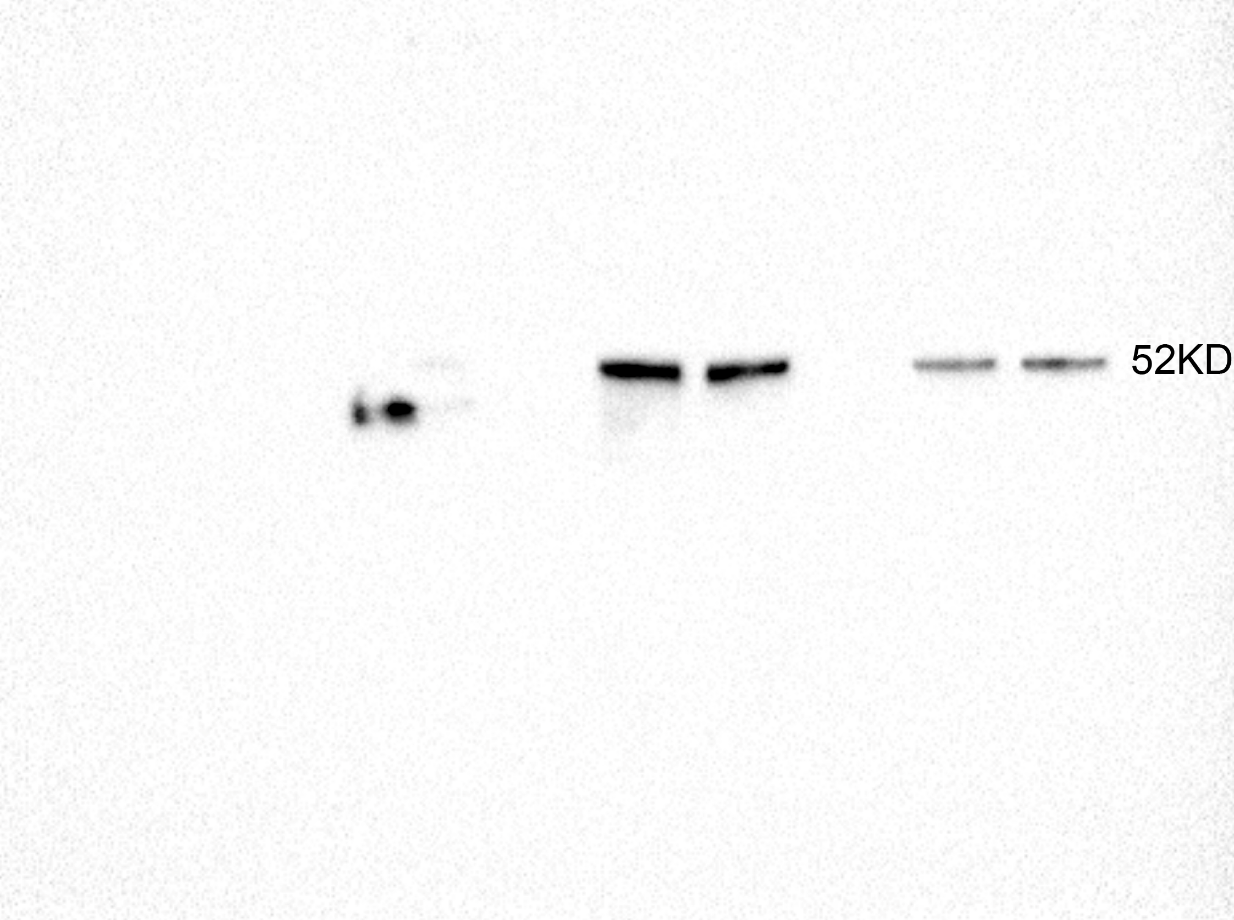


GAPDH


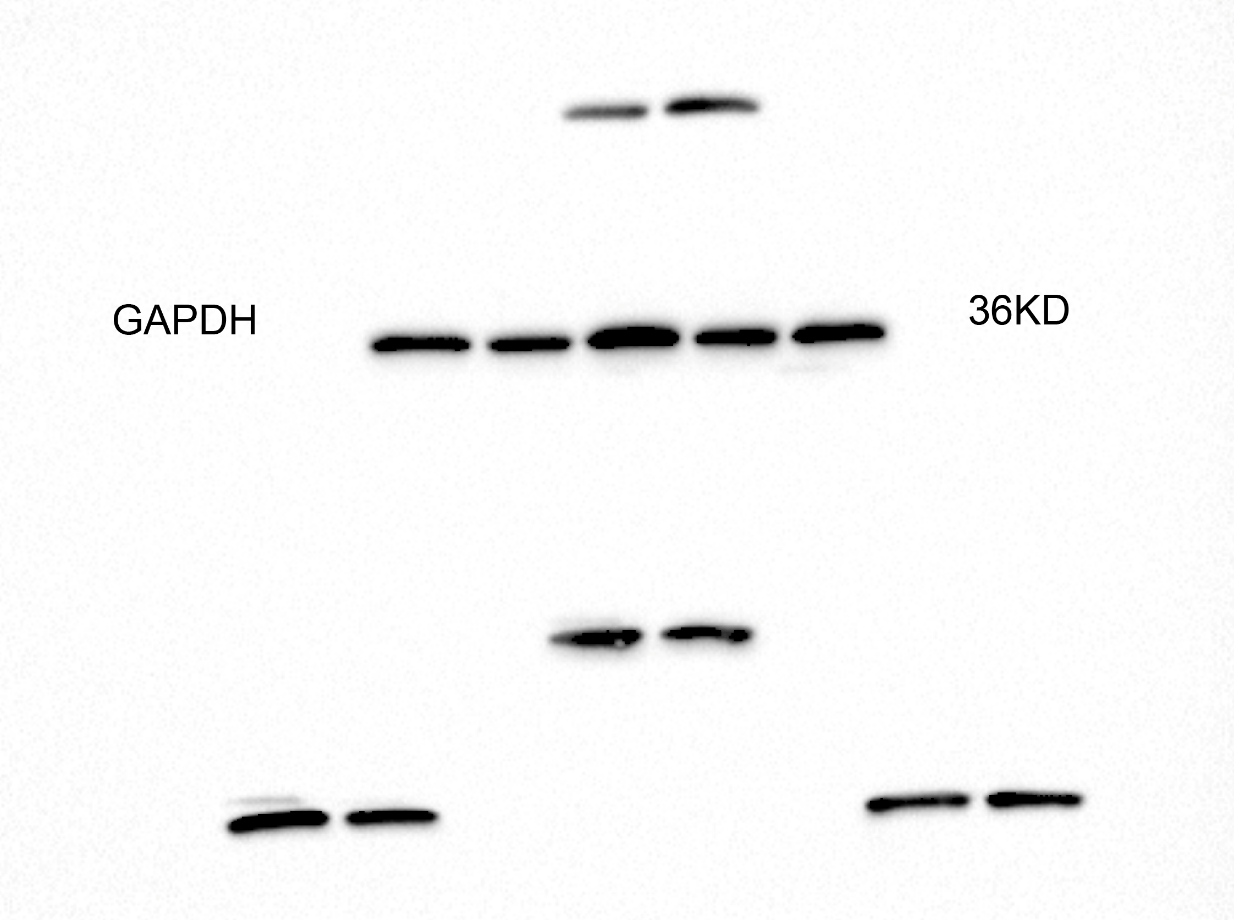


Figure 2-D

NOVA1


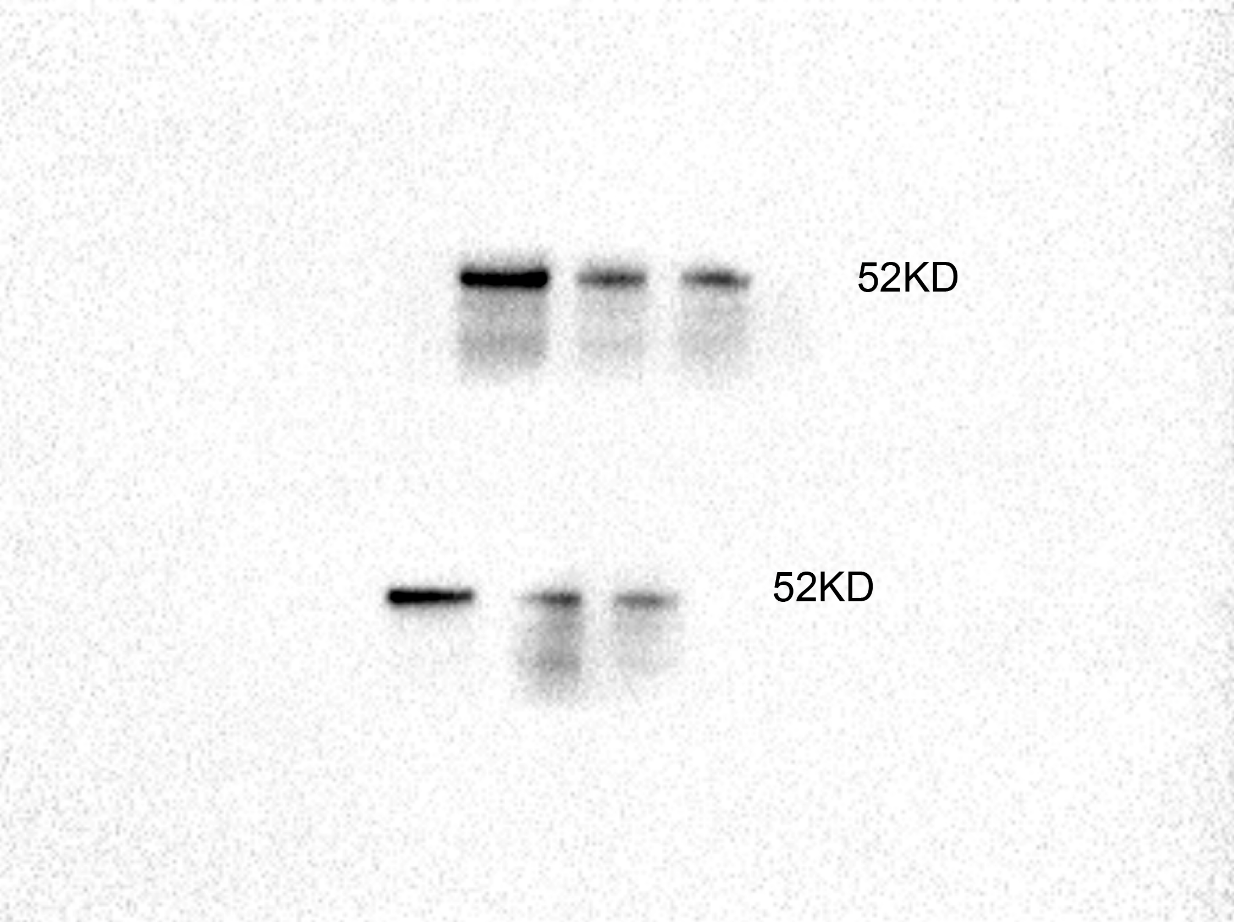


GAPDH


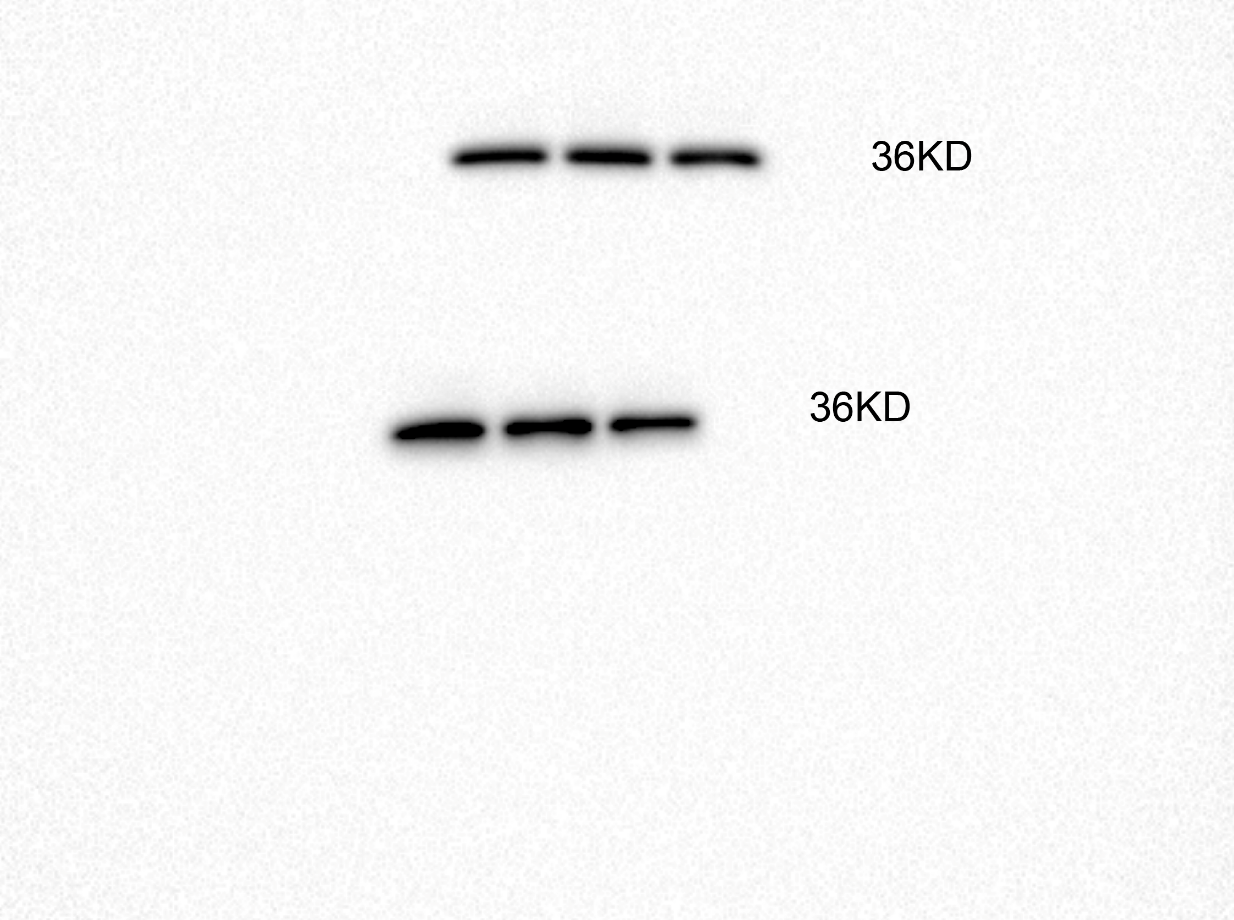


Figure 4

A549-b-catinin-E-ca


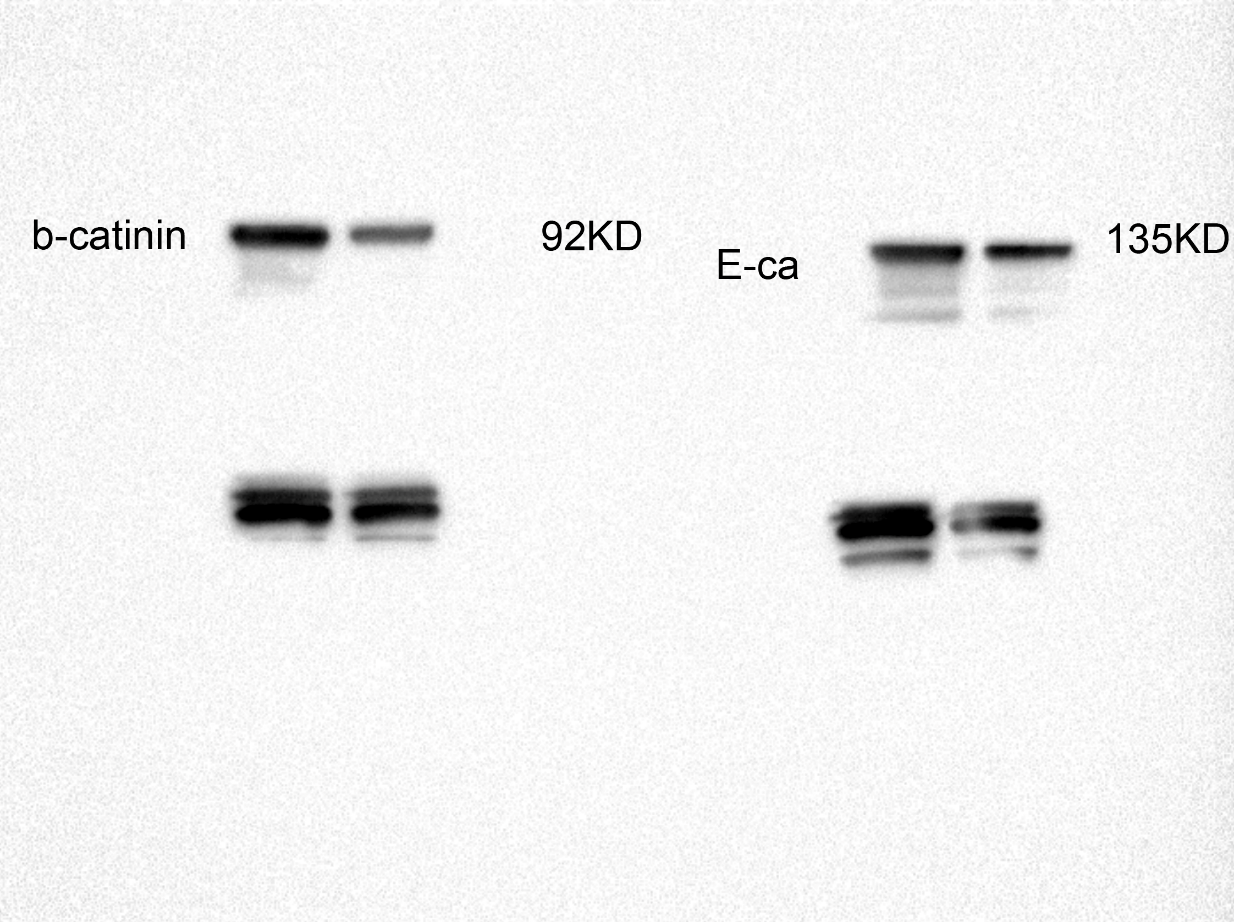


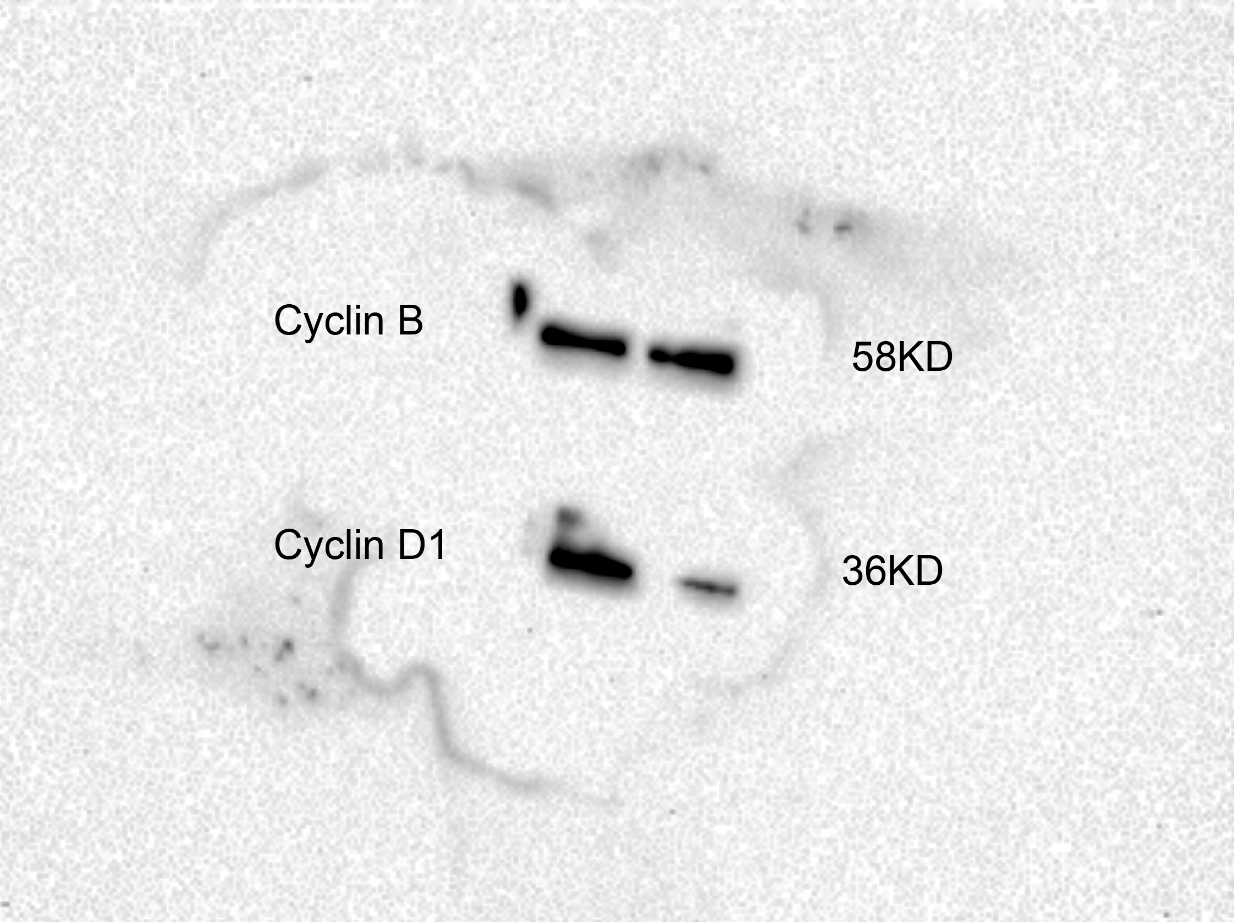


A549-Cyclin D+Cyclin B

A549-GAPDH


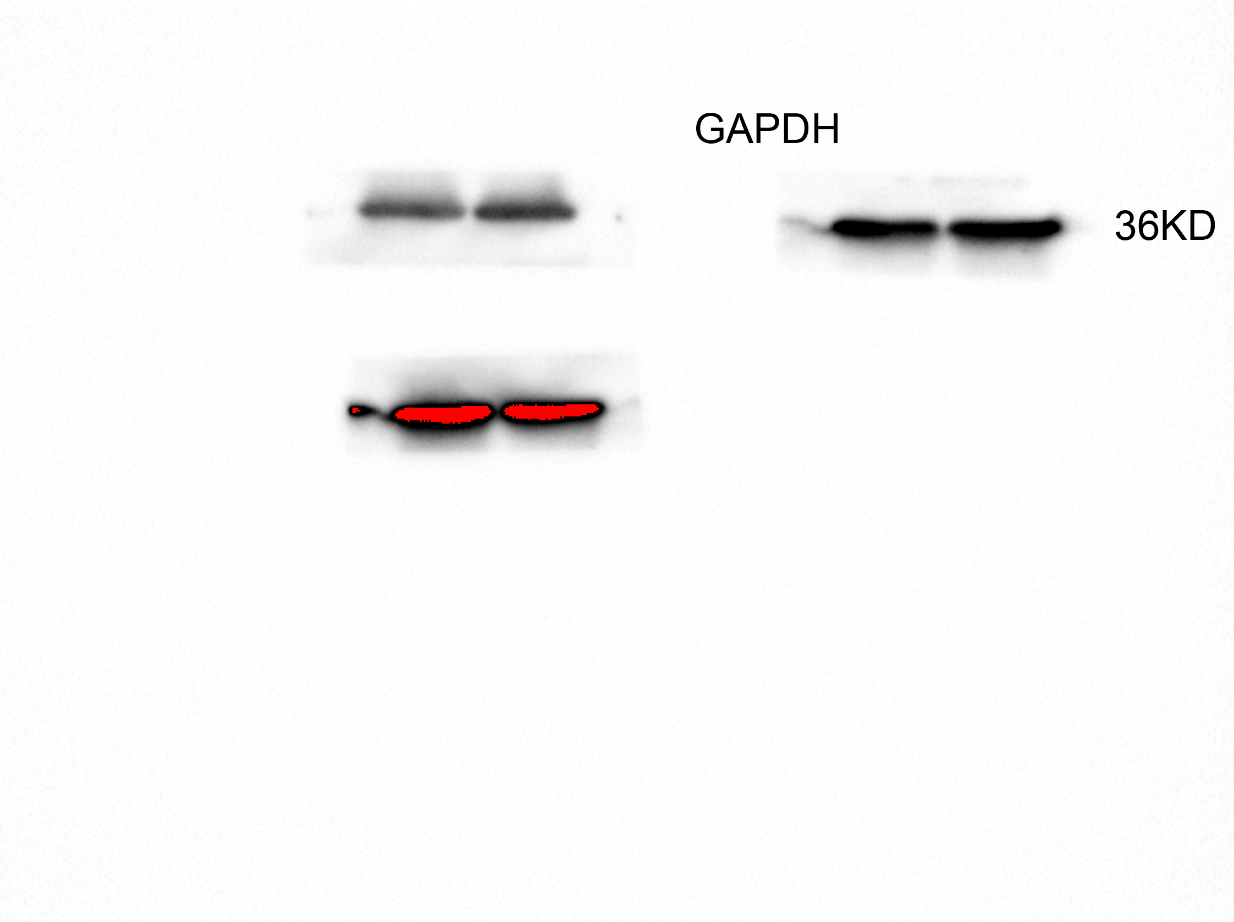


A549-MMP-7


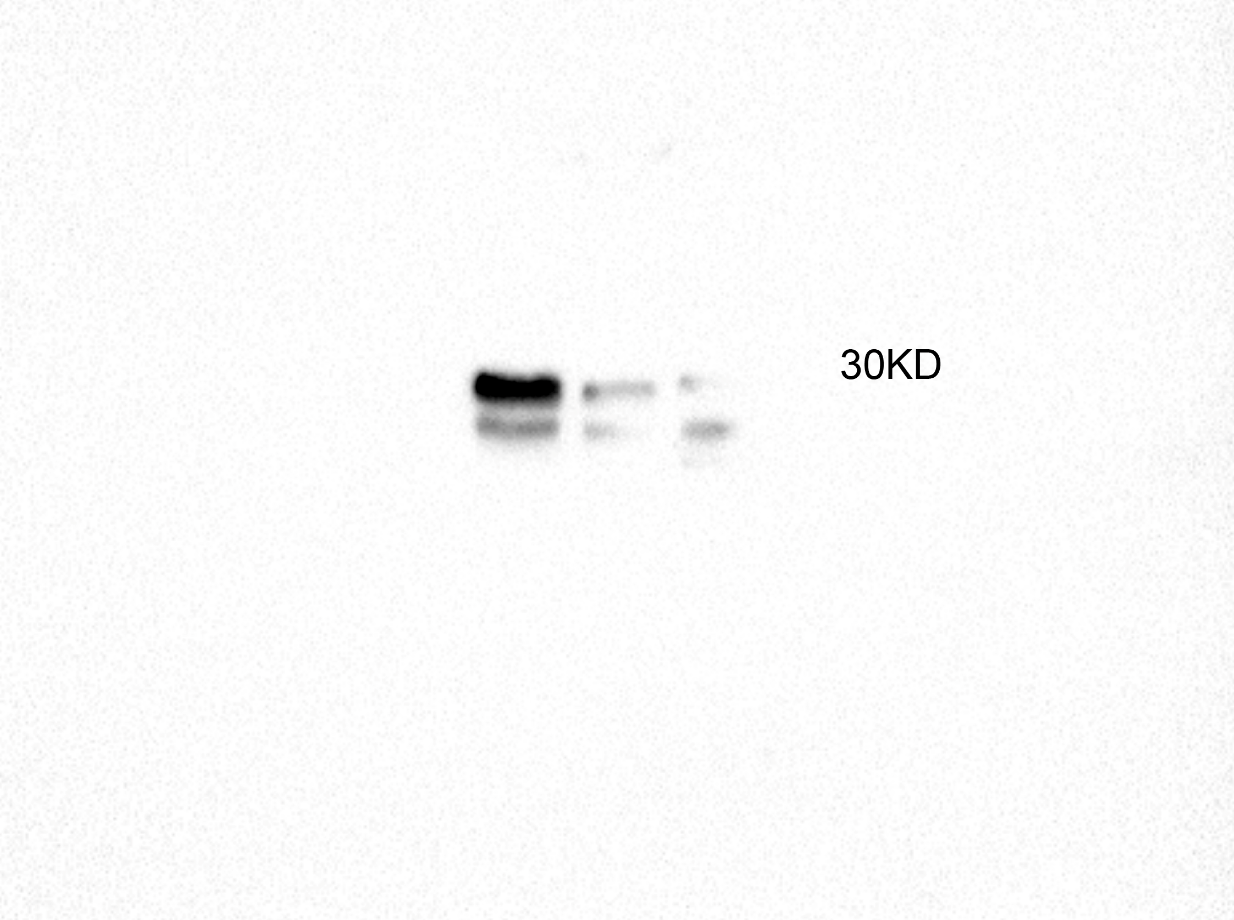


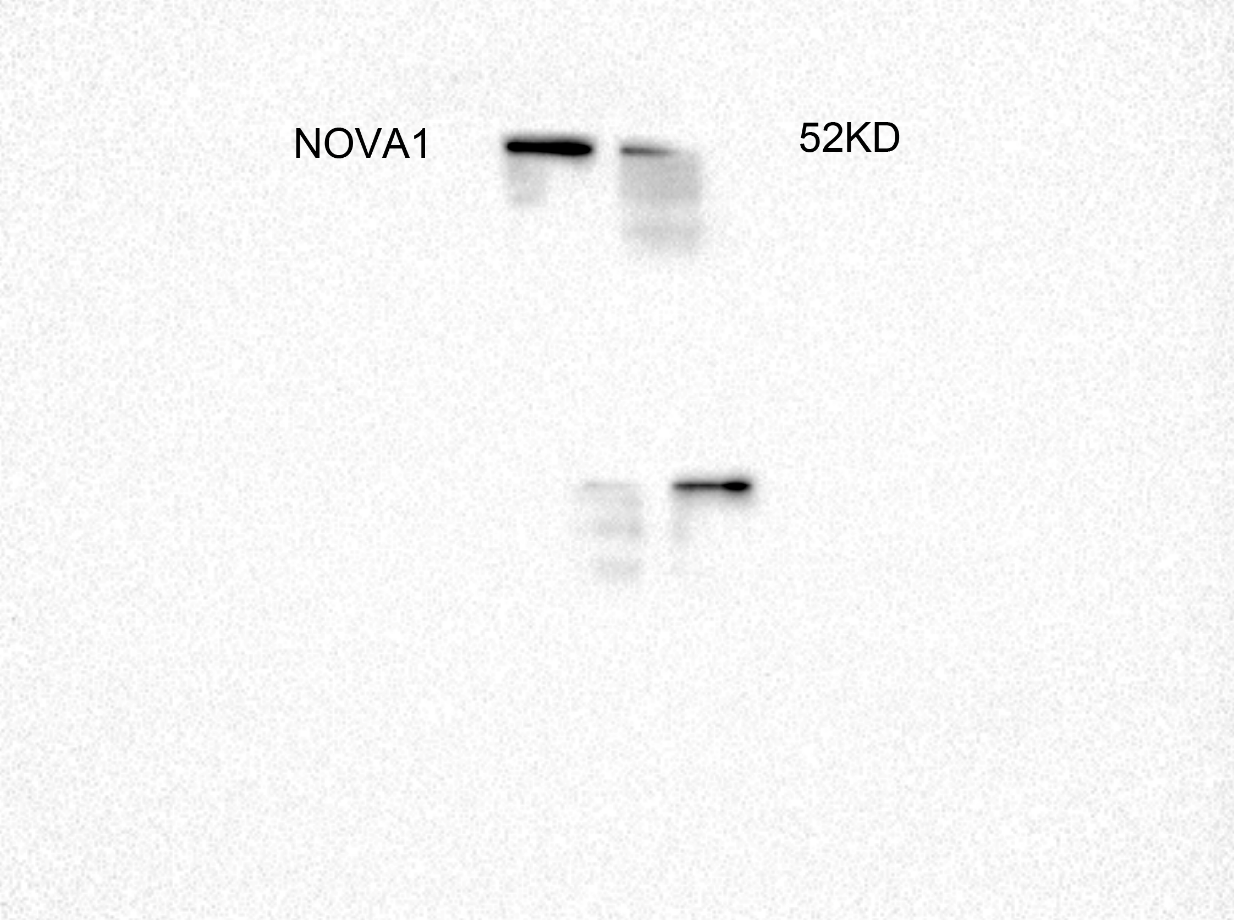


A549-NOVA1

H299-E-cad


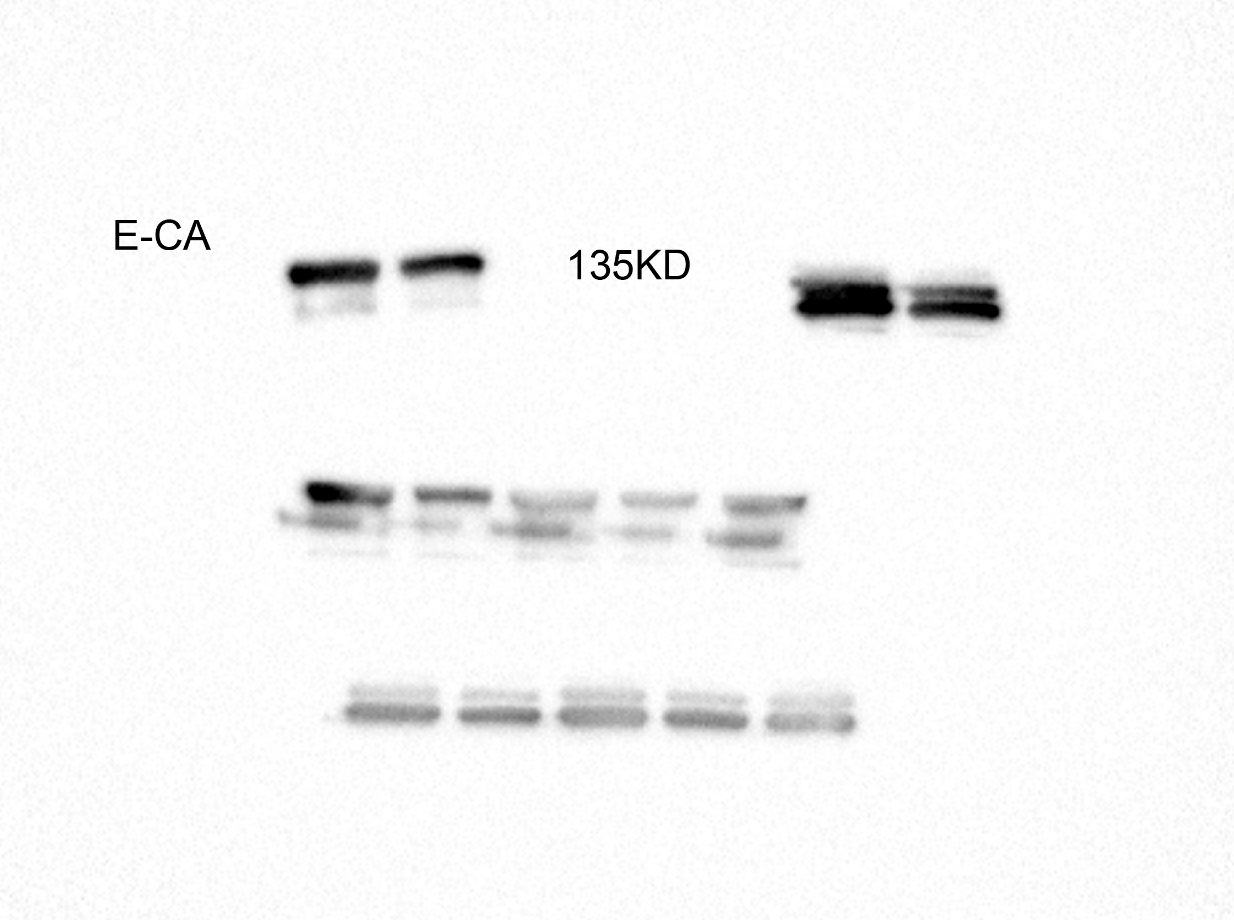


H1299-b-catinin


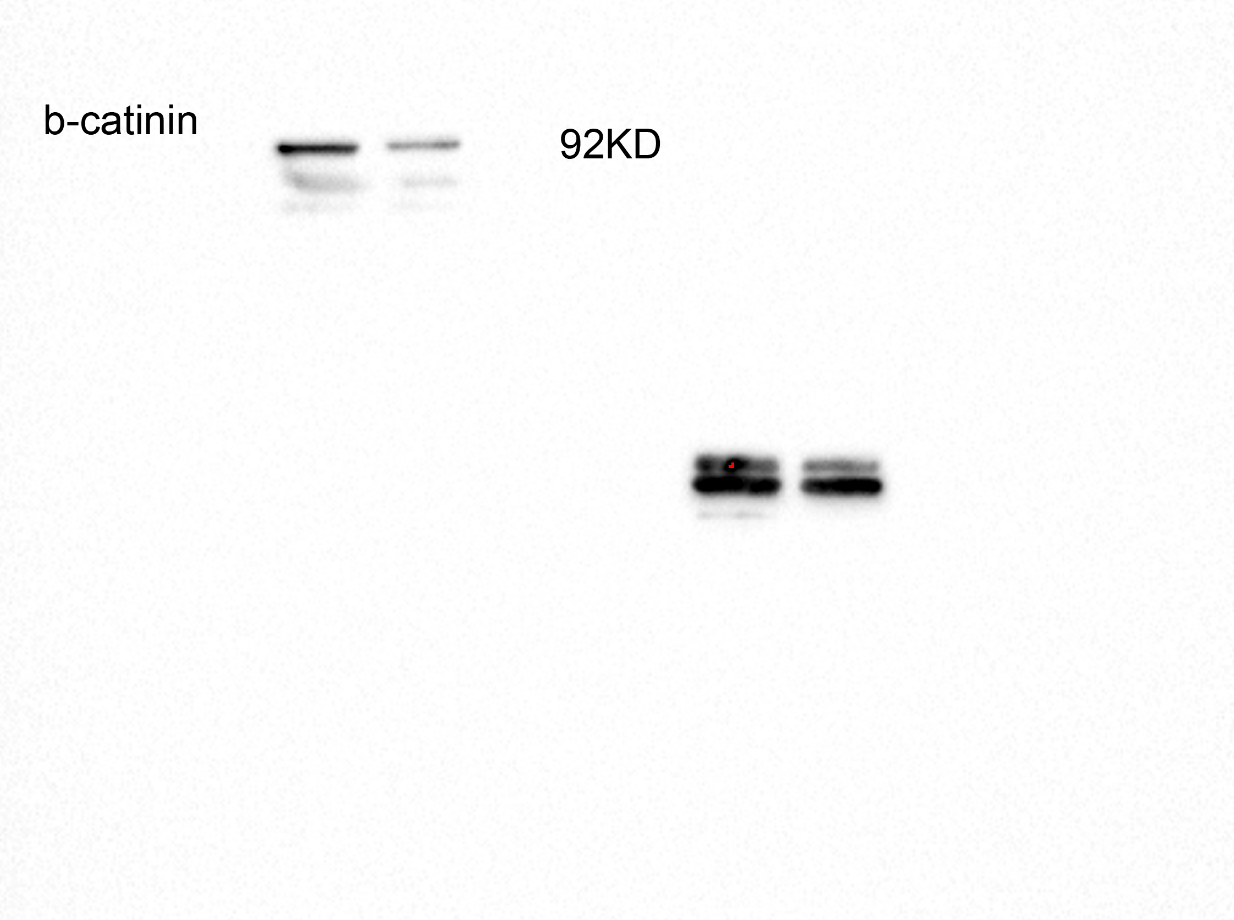


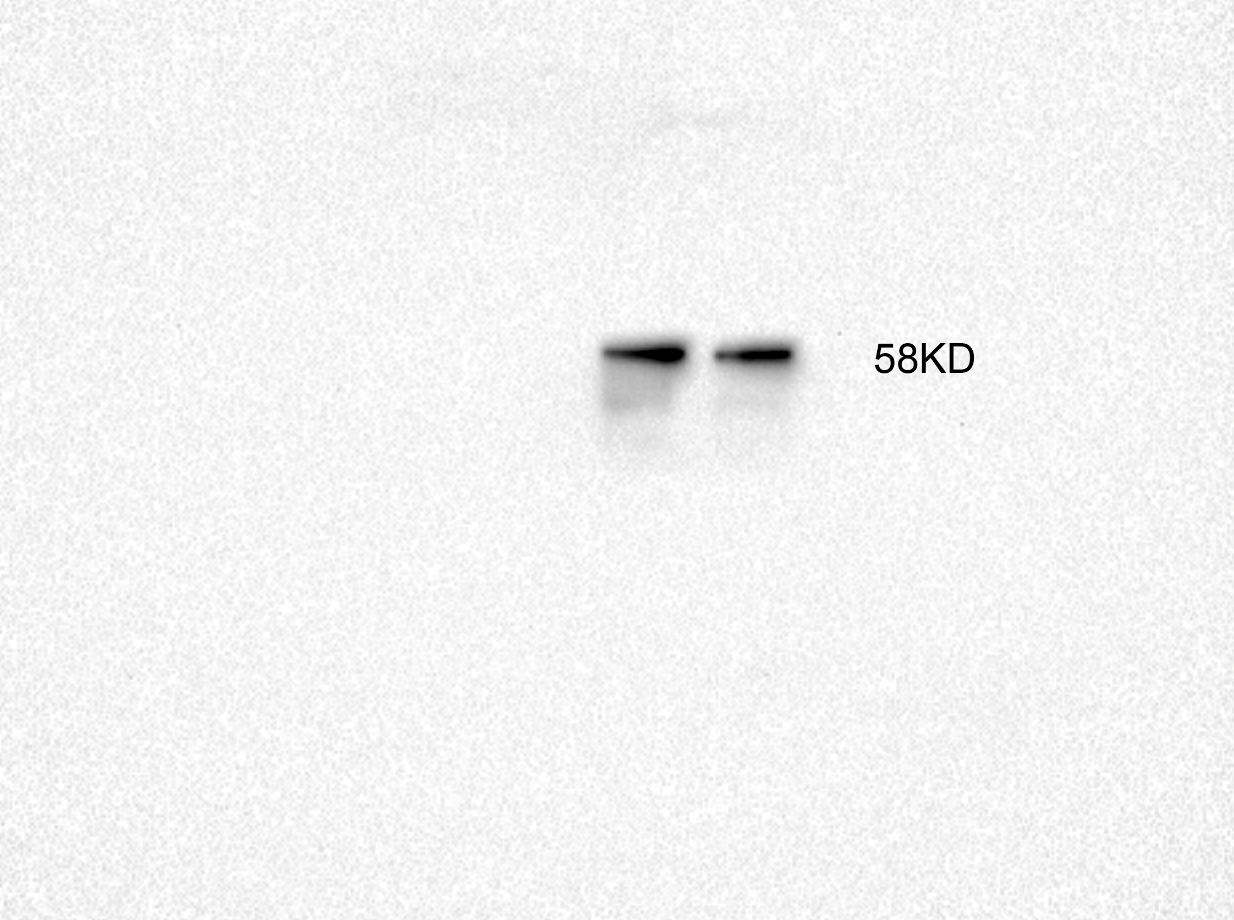


H1299-Cyclin B


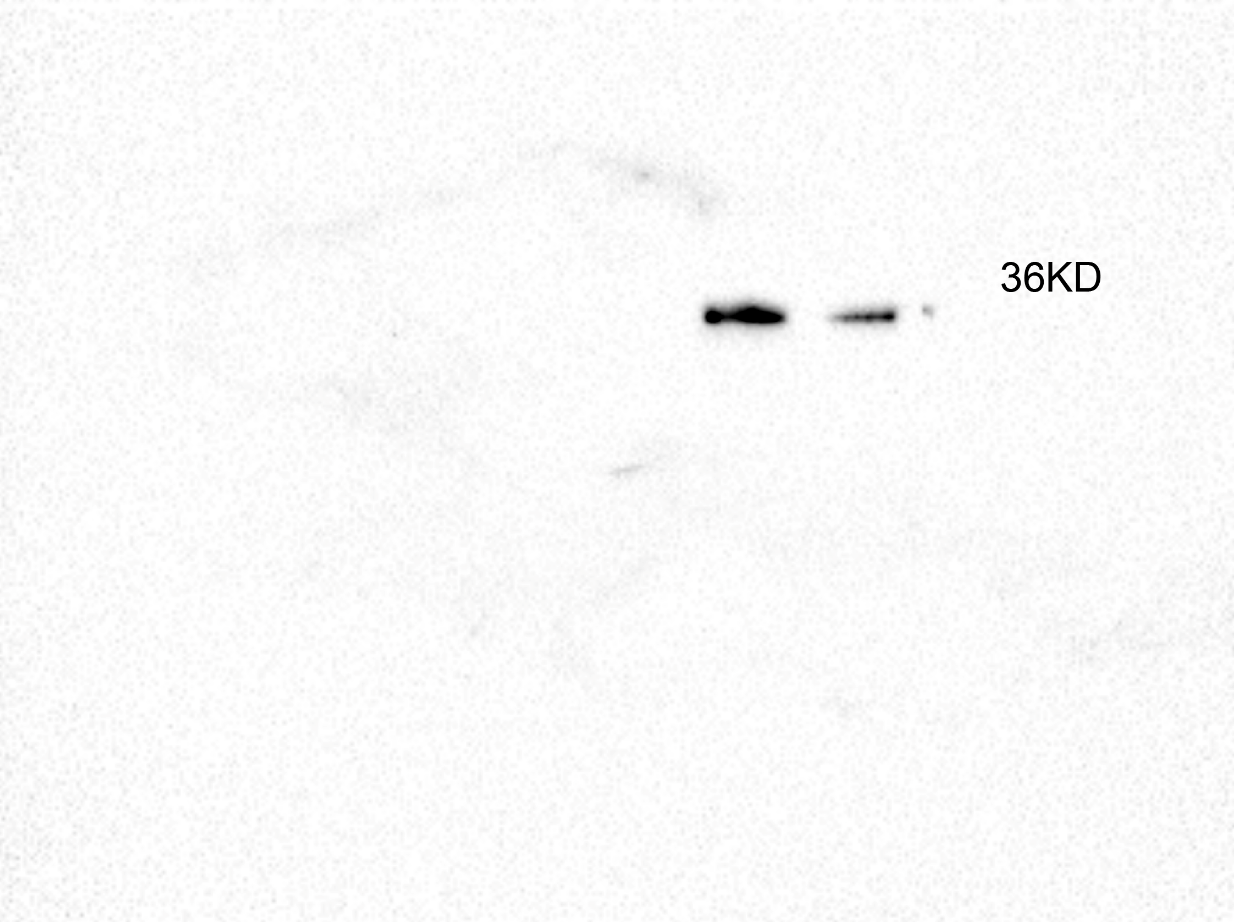


H1299-Cyclin D1

H1299-GADPH


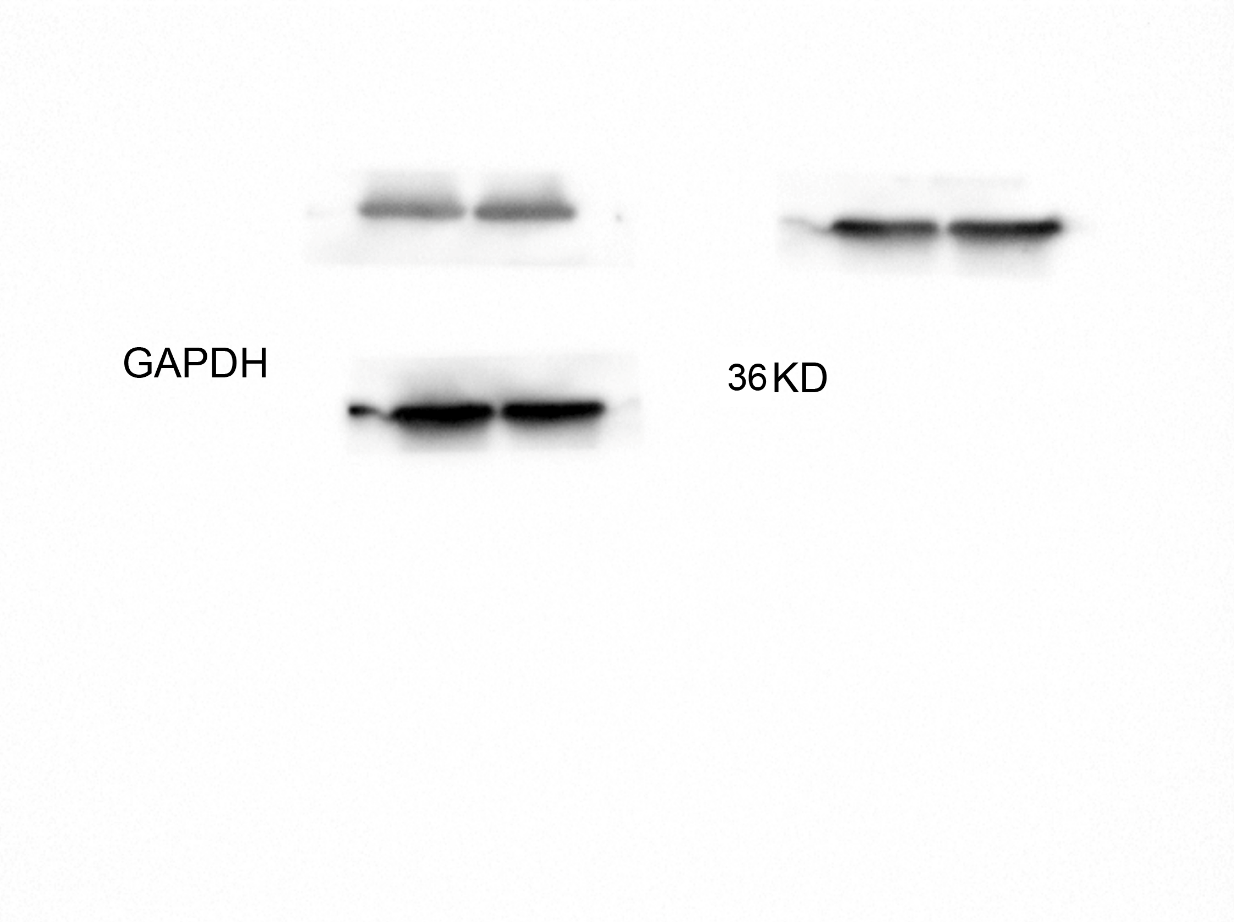


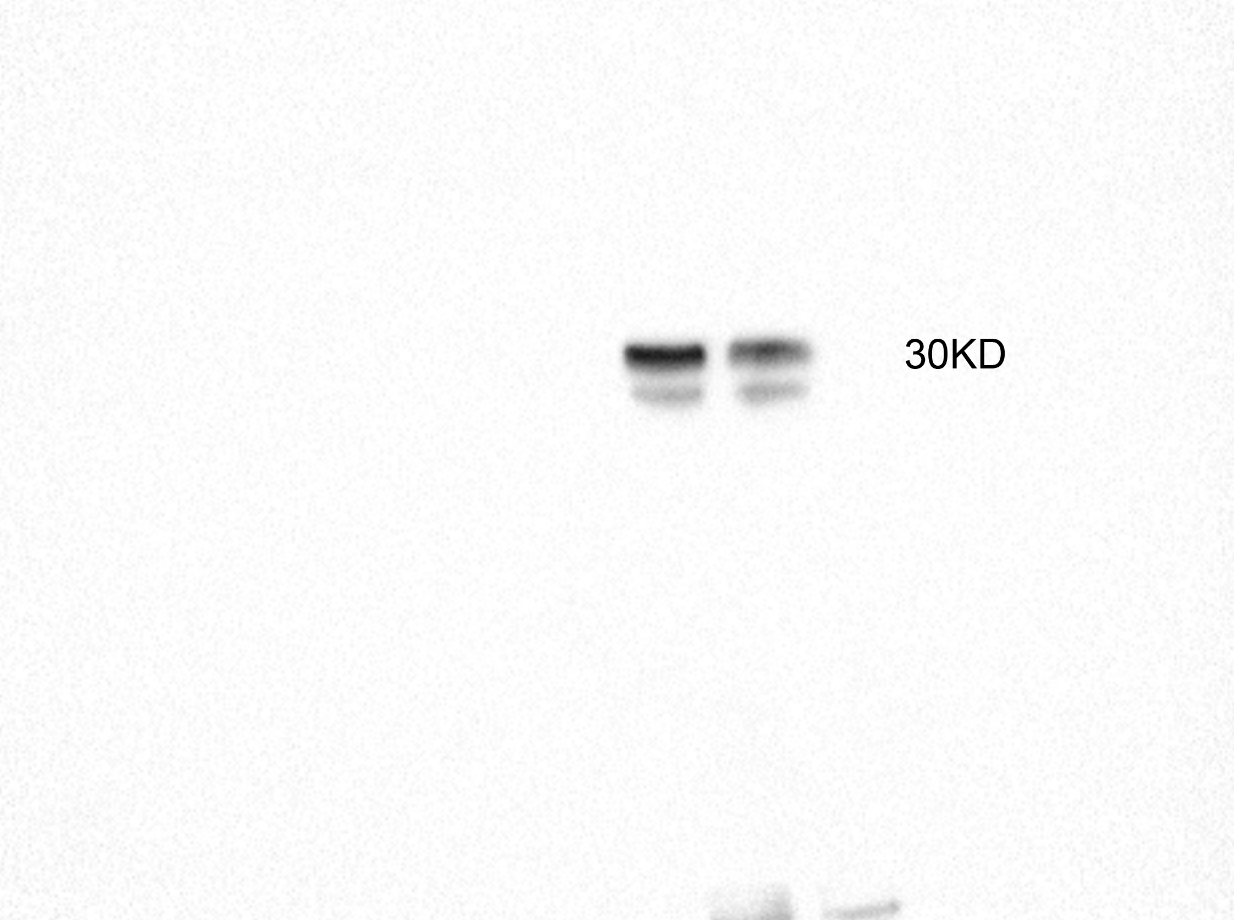


H1299-MMP-7

H1299-NOVA1


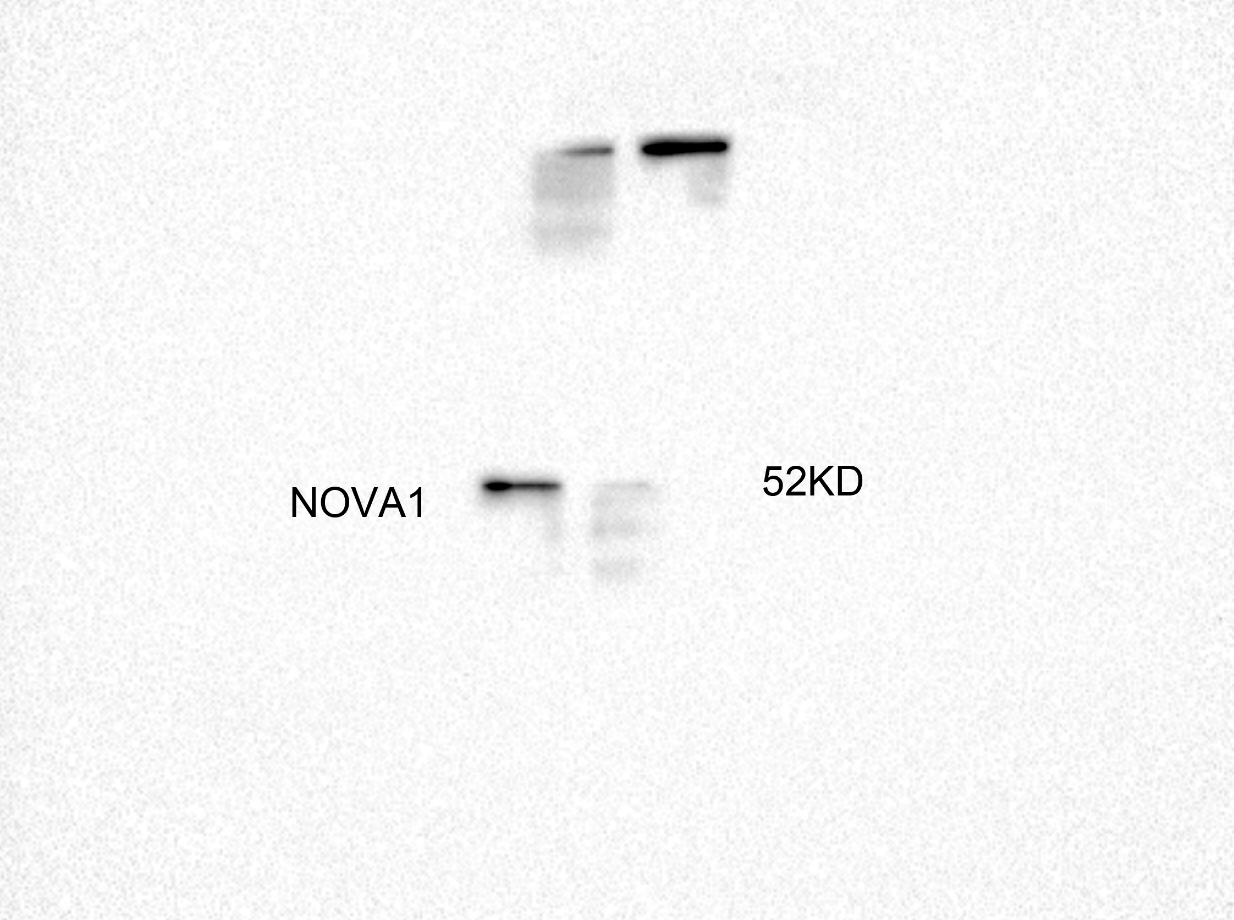


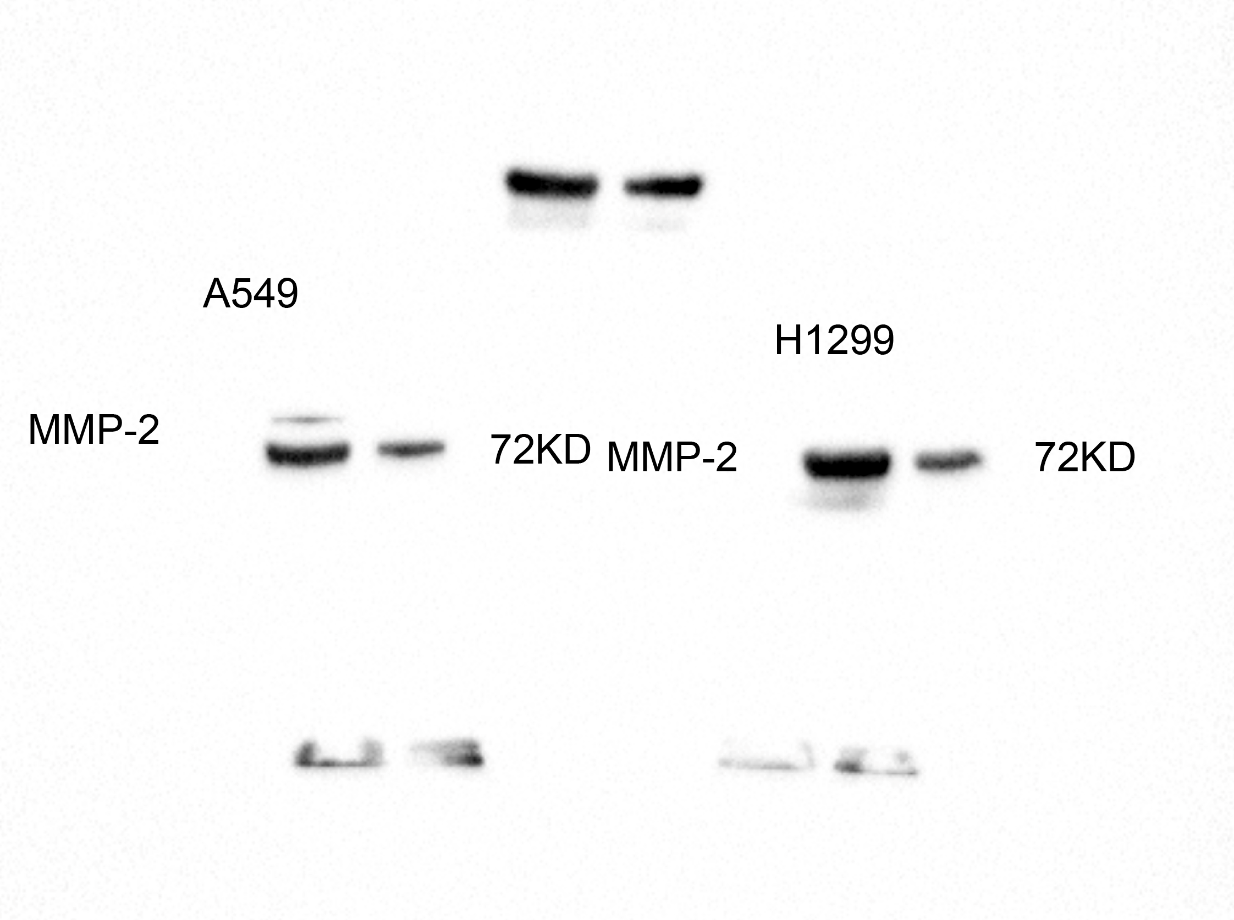


MMP-2

Figure 5B

Figure5B-A549-A-b-catinin


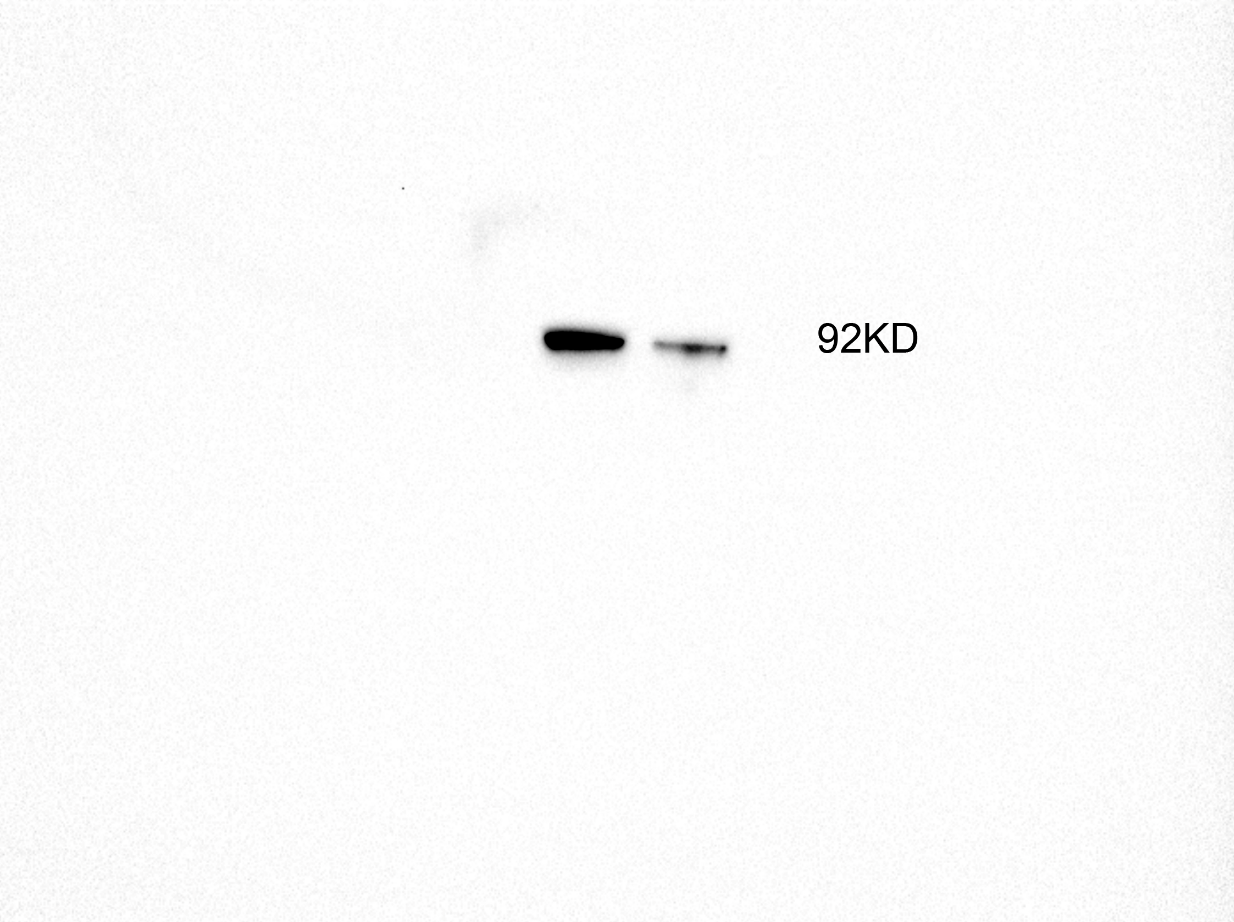


Figure5B-H1299-A-b-catinin


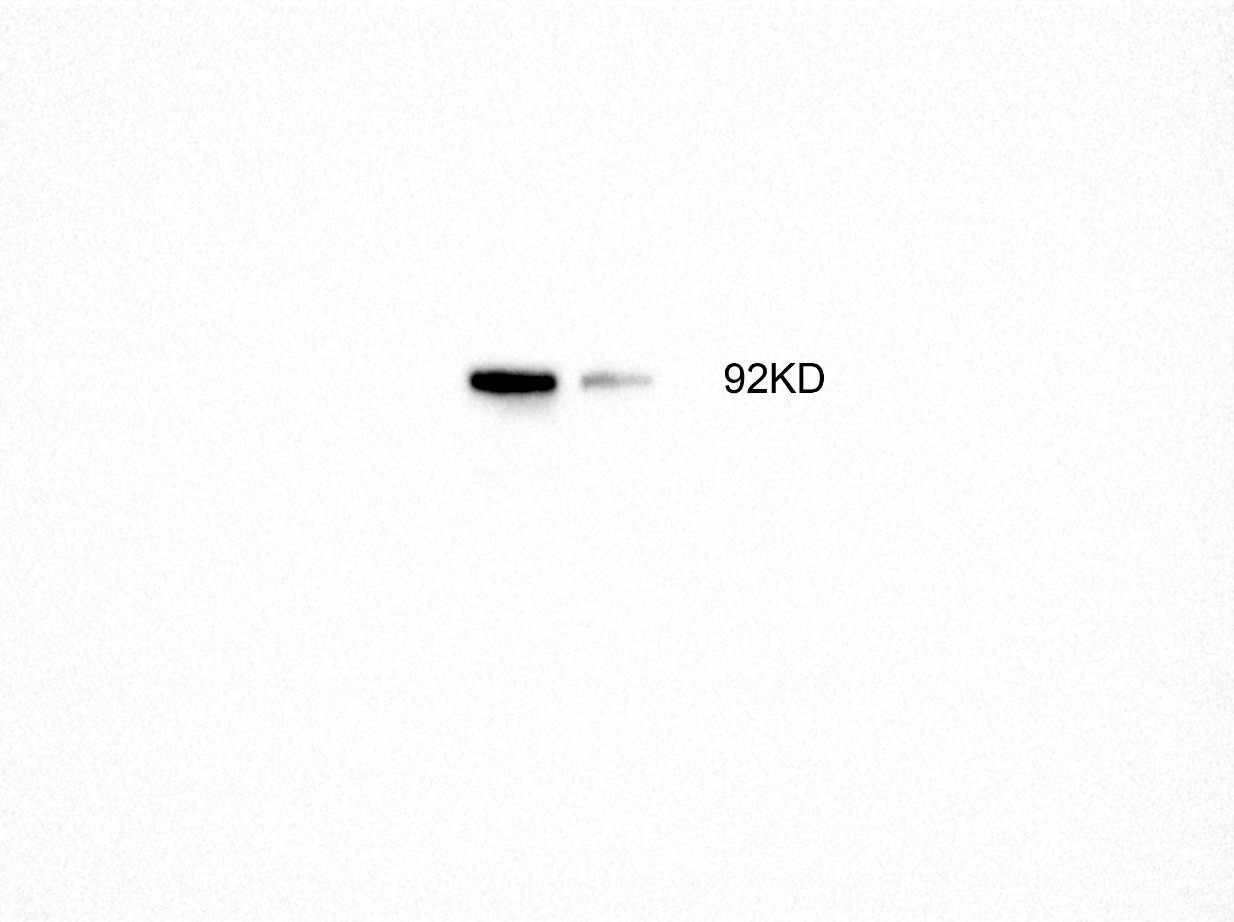


5B-A549-b-catinin


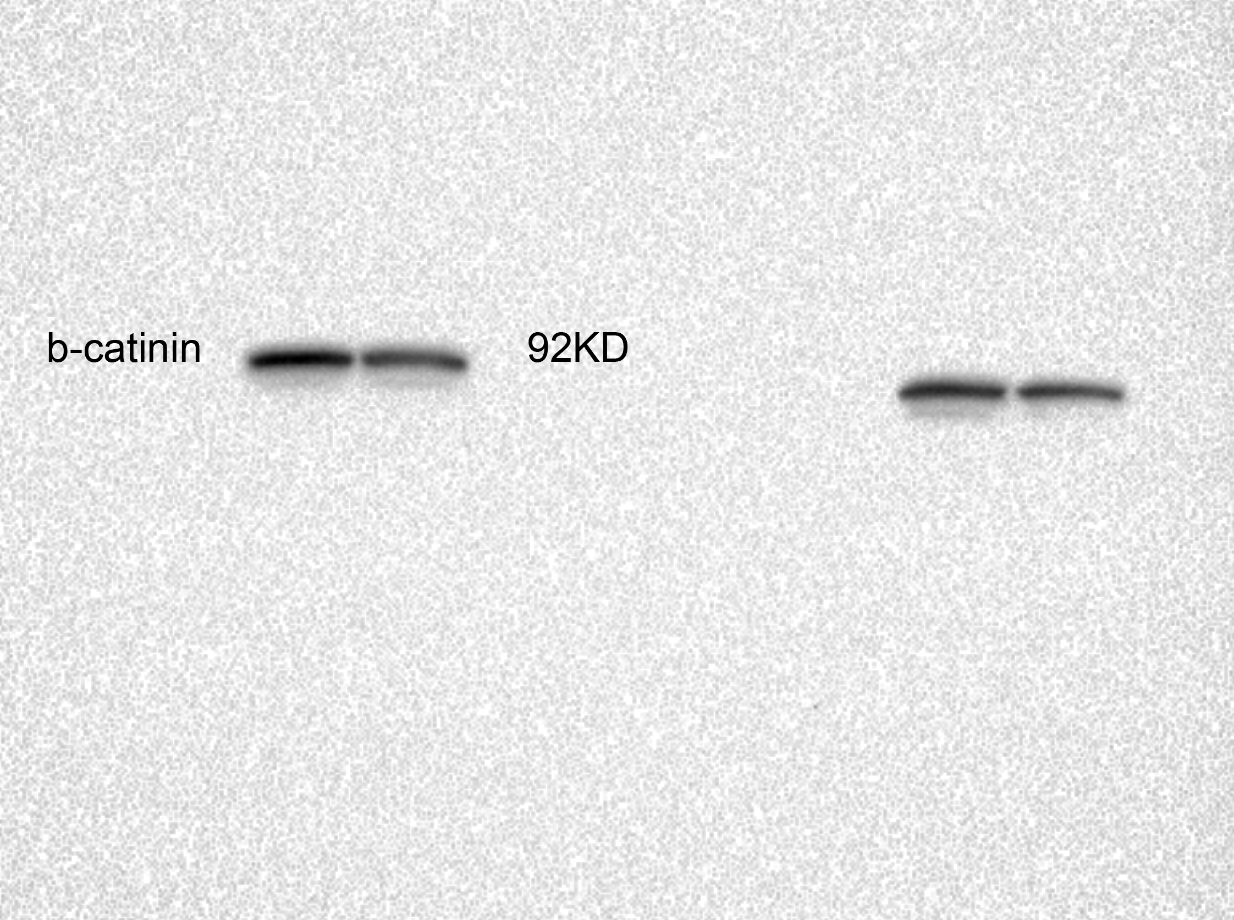


5B-H1299-b-catinin


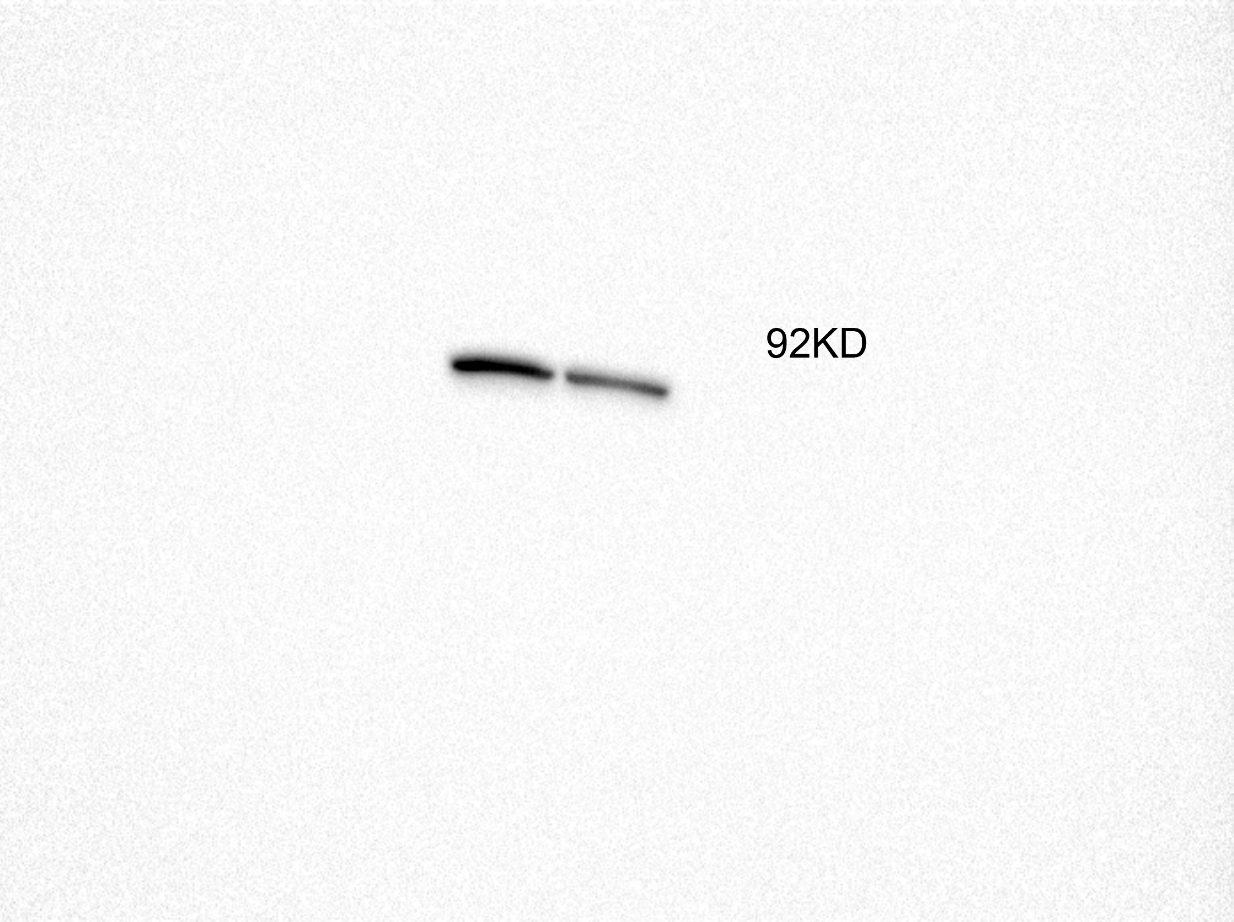


Figure5B-H1299-GADPH


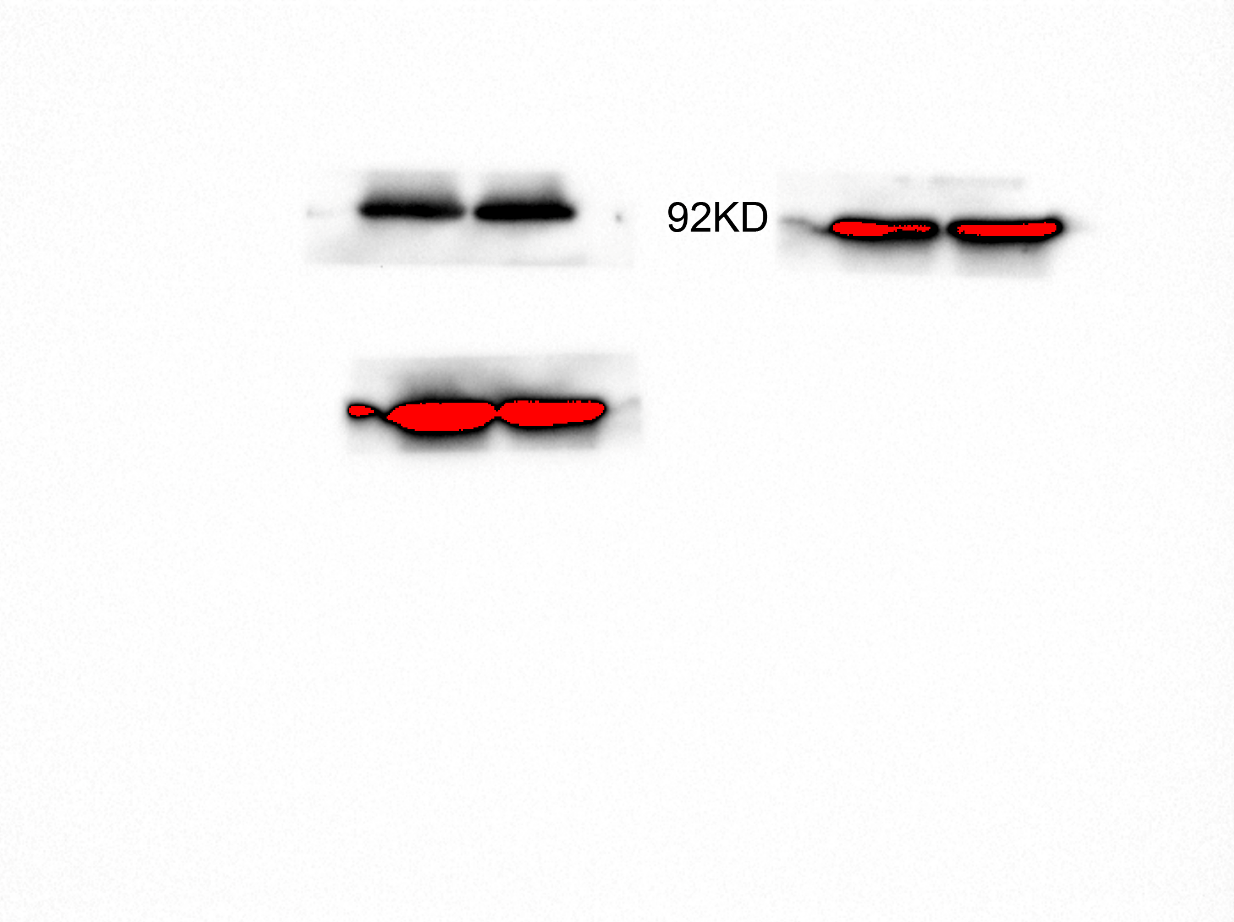


Figure5 C

Figure5B-A549-GADPH+5C-H1299-GAPDH


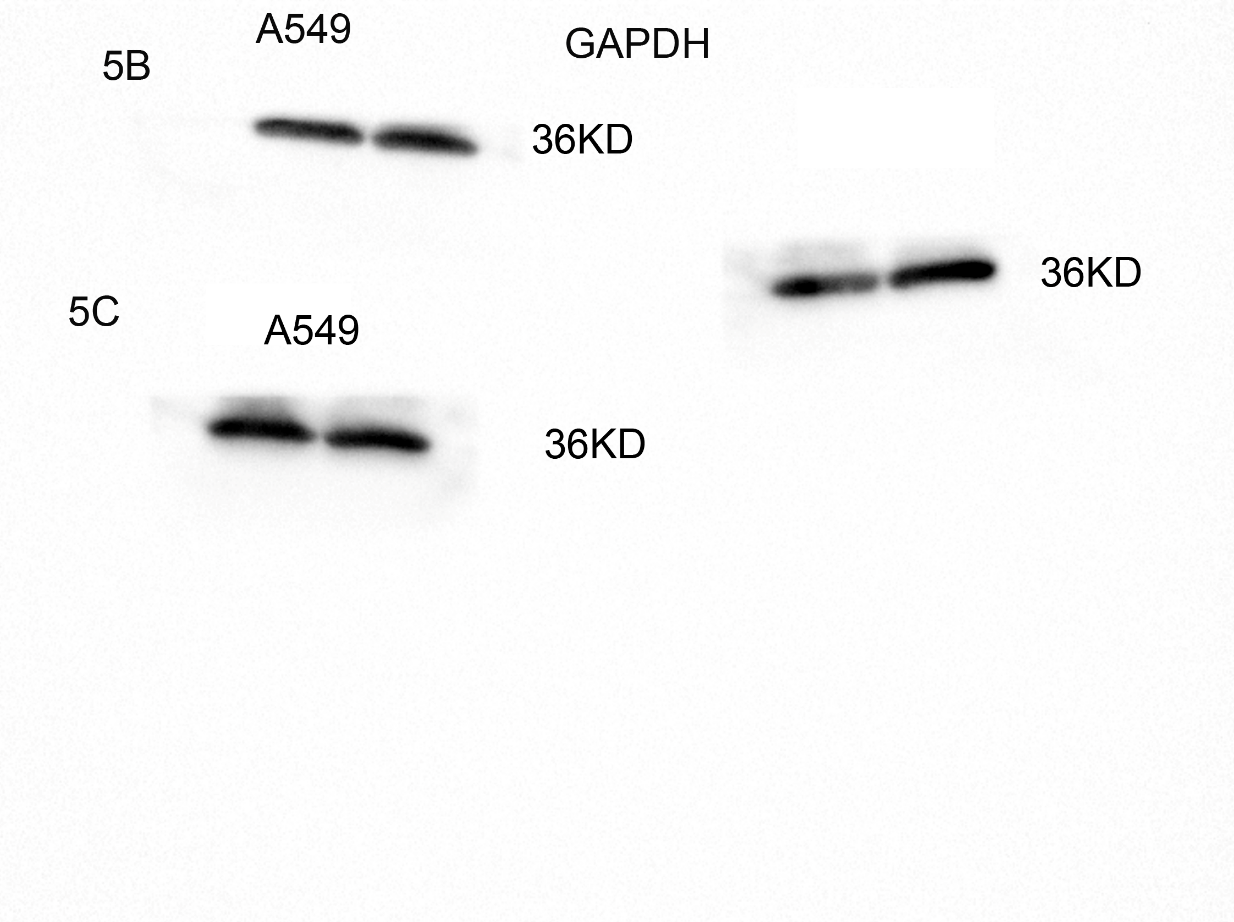


Figure5C-H1299-GADPH


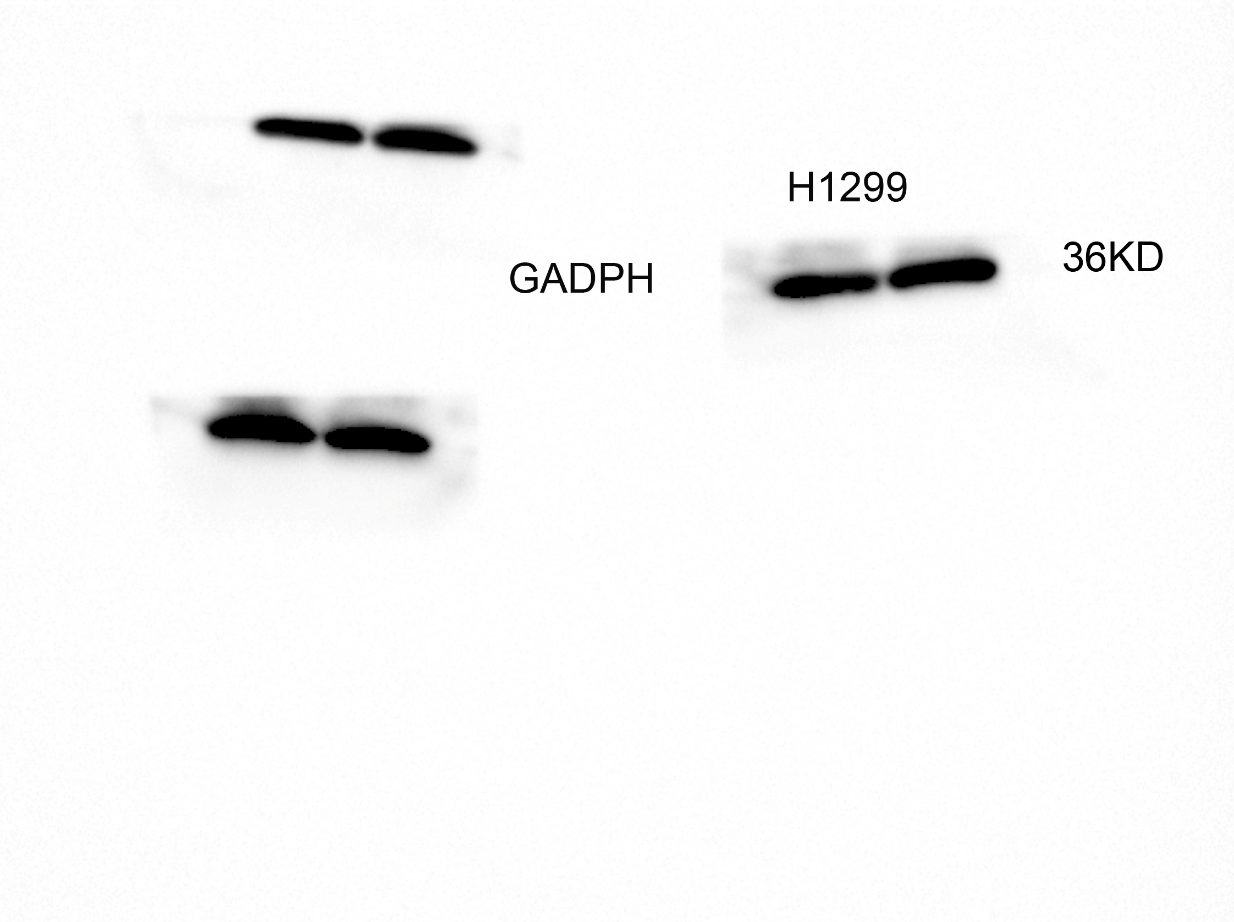


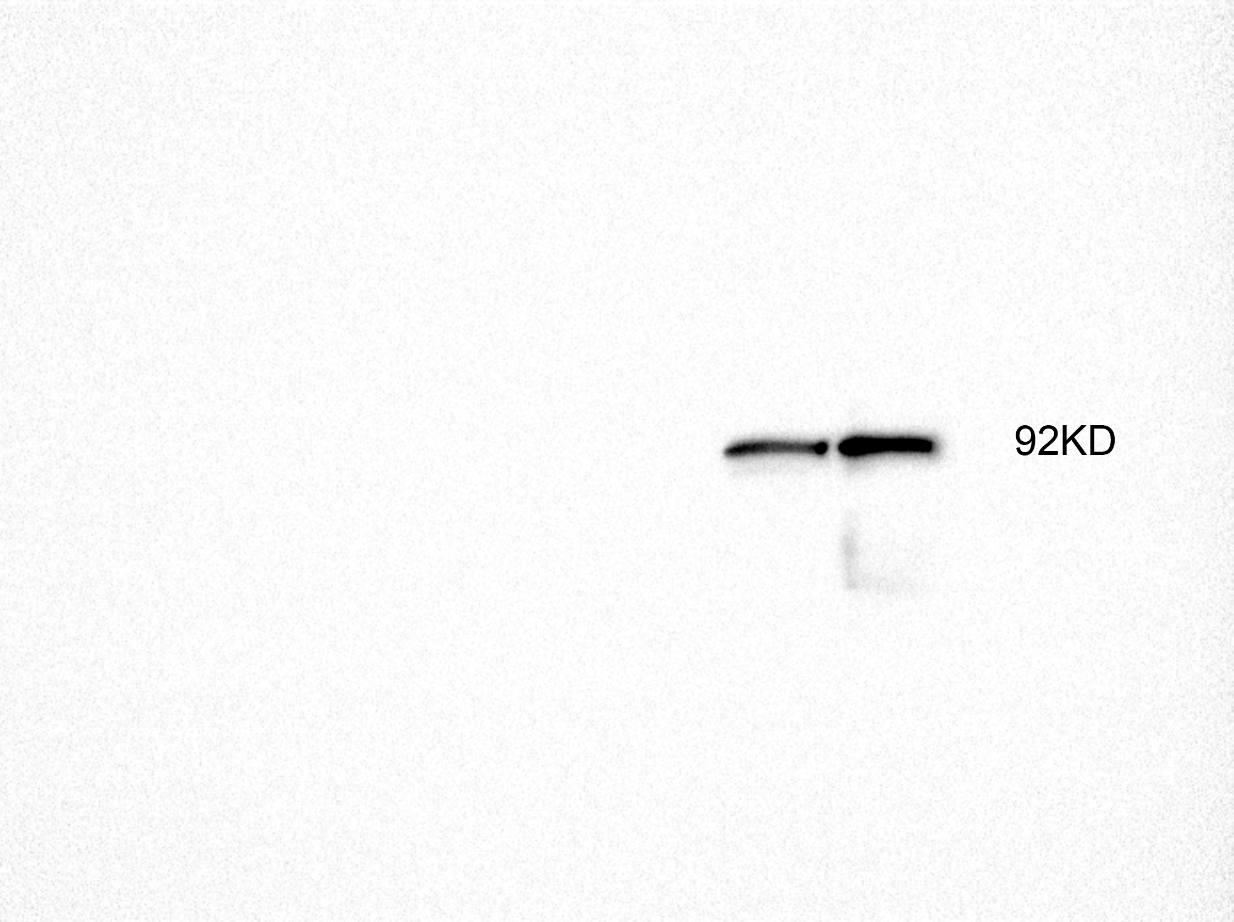


Figure5C A549-b-catinin

Figure5C-H1299-b-catinin


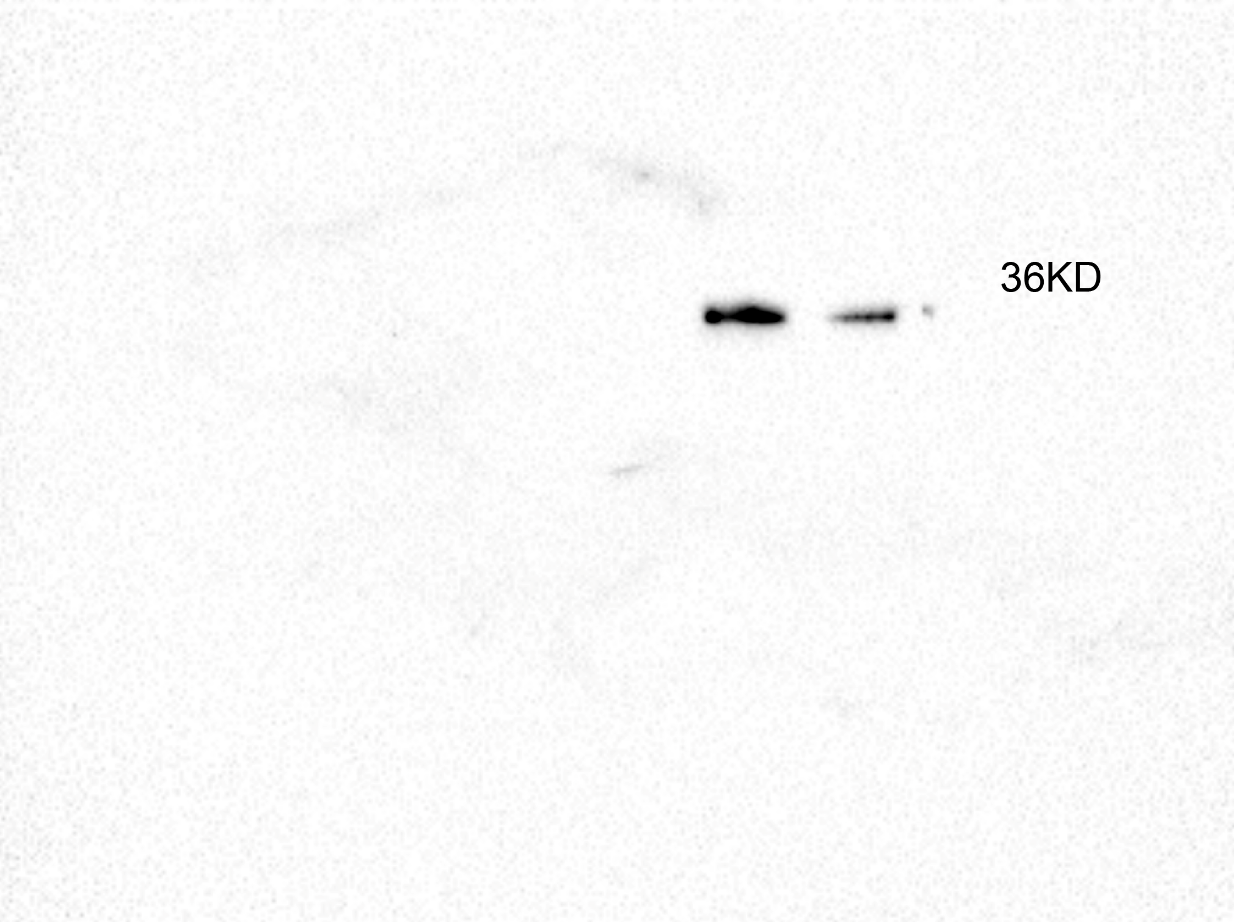

Supplement: Supplementary file 1 — Additional file 1: Supplementary material. Original images of WB-1. Original images of WB-2. [file 12885_2022_10164_MOESM1_ESM.zip › Supplementary material-Original images of WB-1.docx]
