# Supplementary material for: NOVA1 promotes NSCLC proliferation and invasion by activating Wnt/β-catenin signaling
Source: BMC Cancer. 2022 Oct 25;22:1091. doi: 10.1186/s12885-022-10164-8 (PMC9594932; doi:10.1186/s12885-022-10164-8)
Supplement: Supplementary file 1 — Additional file 1: Supplementary material. Original images of WB-1. Original images of WB-2. [file 12885_2022_10164_MOESM1_ESM.zip › Supplementary material-Original images of WB-2.docx]

**Fuller-length, original, unprocessed blot  and of replicate blots of** **NOVA1**


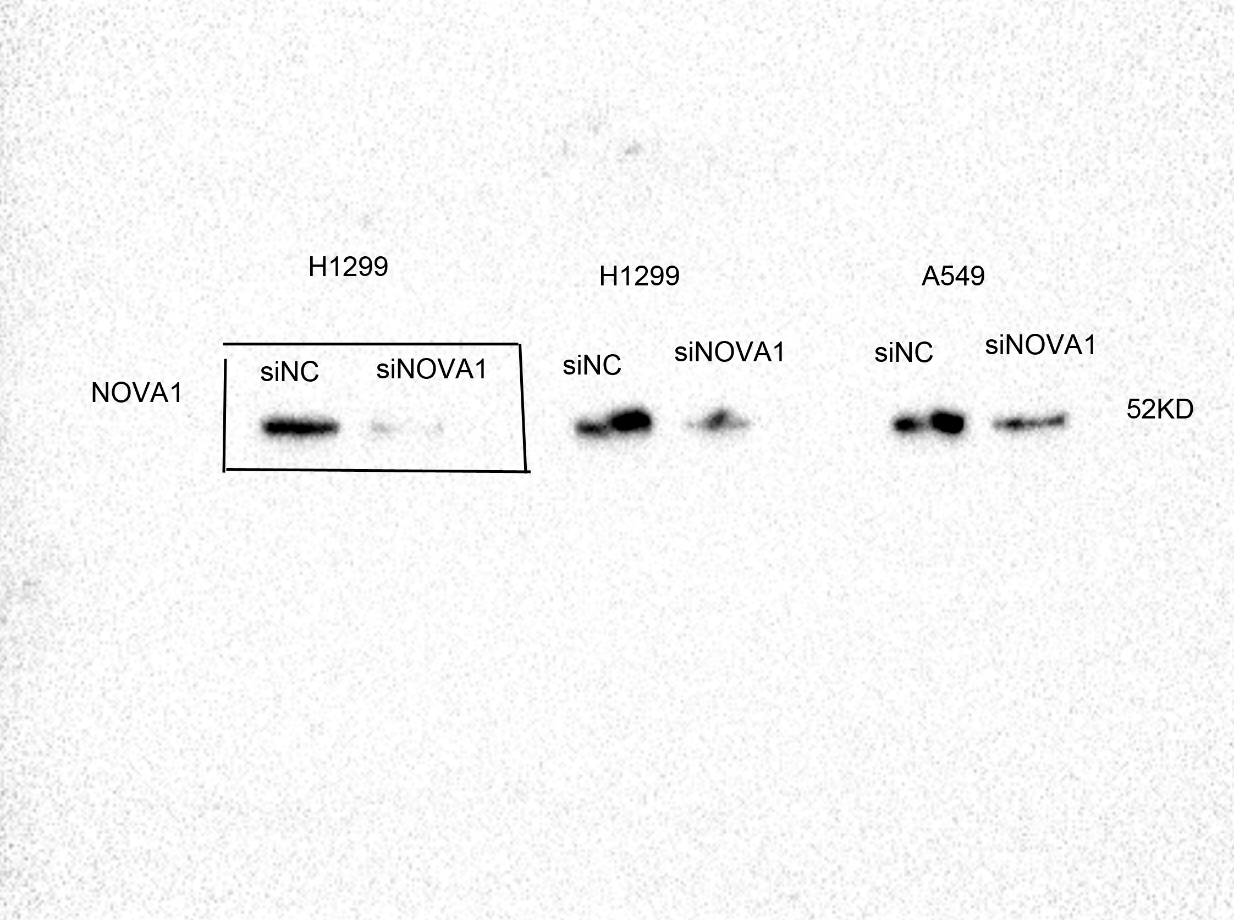

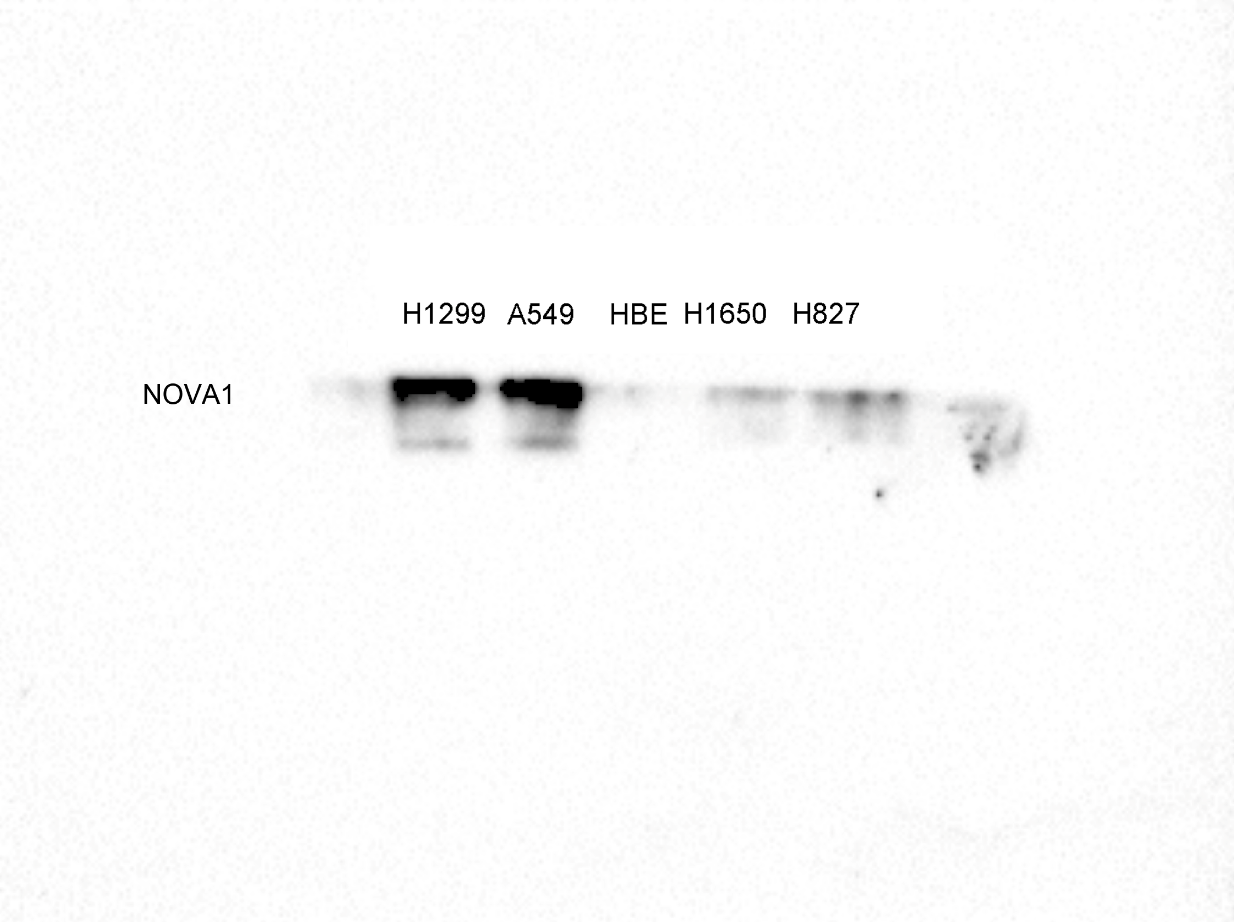

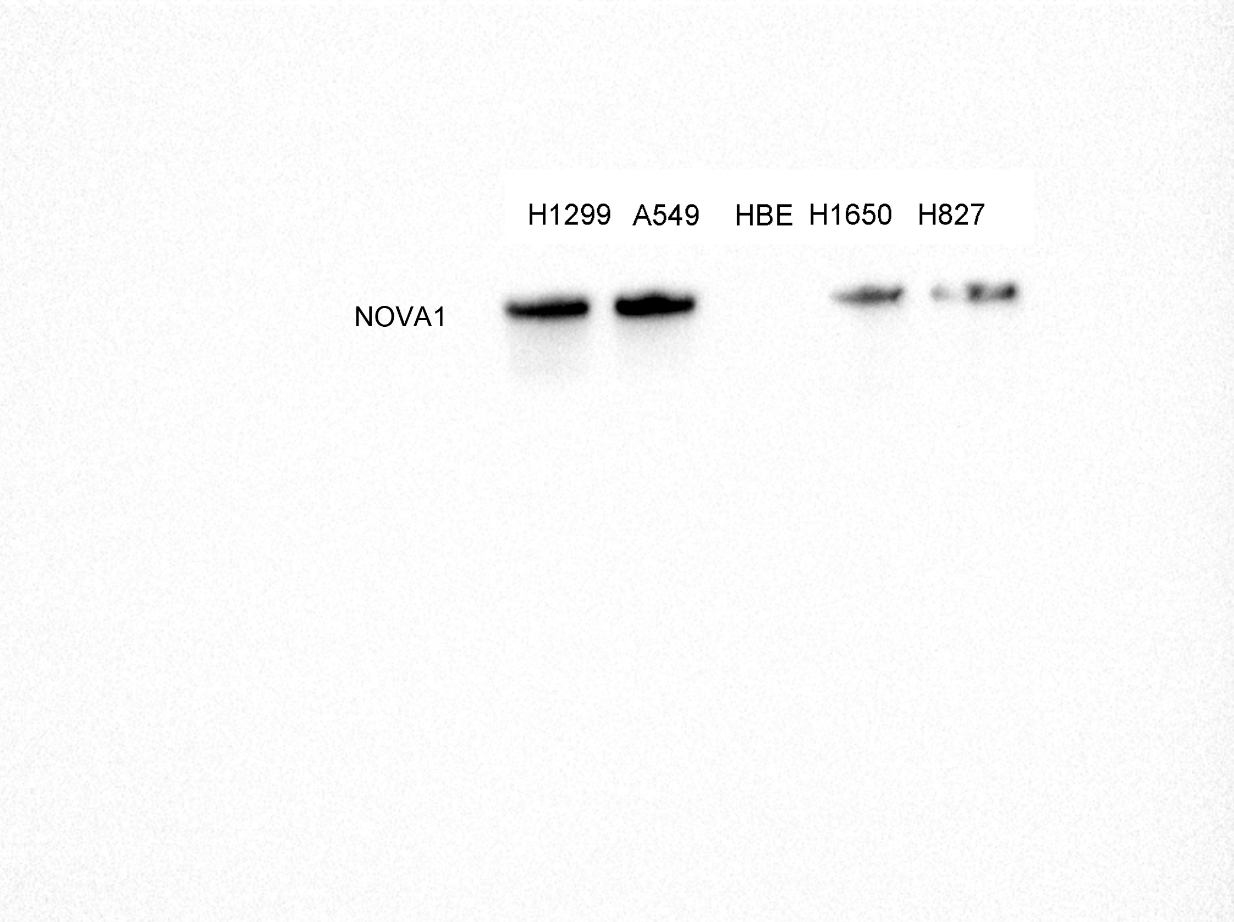


**Fuller-length, original, unprocessed blot  and replicate blots of GAPDH**


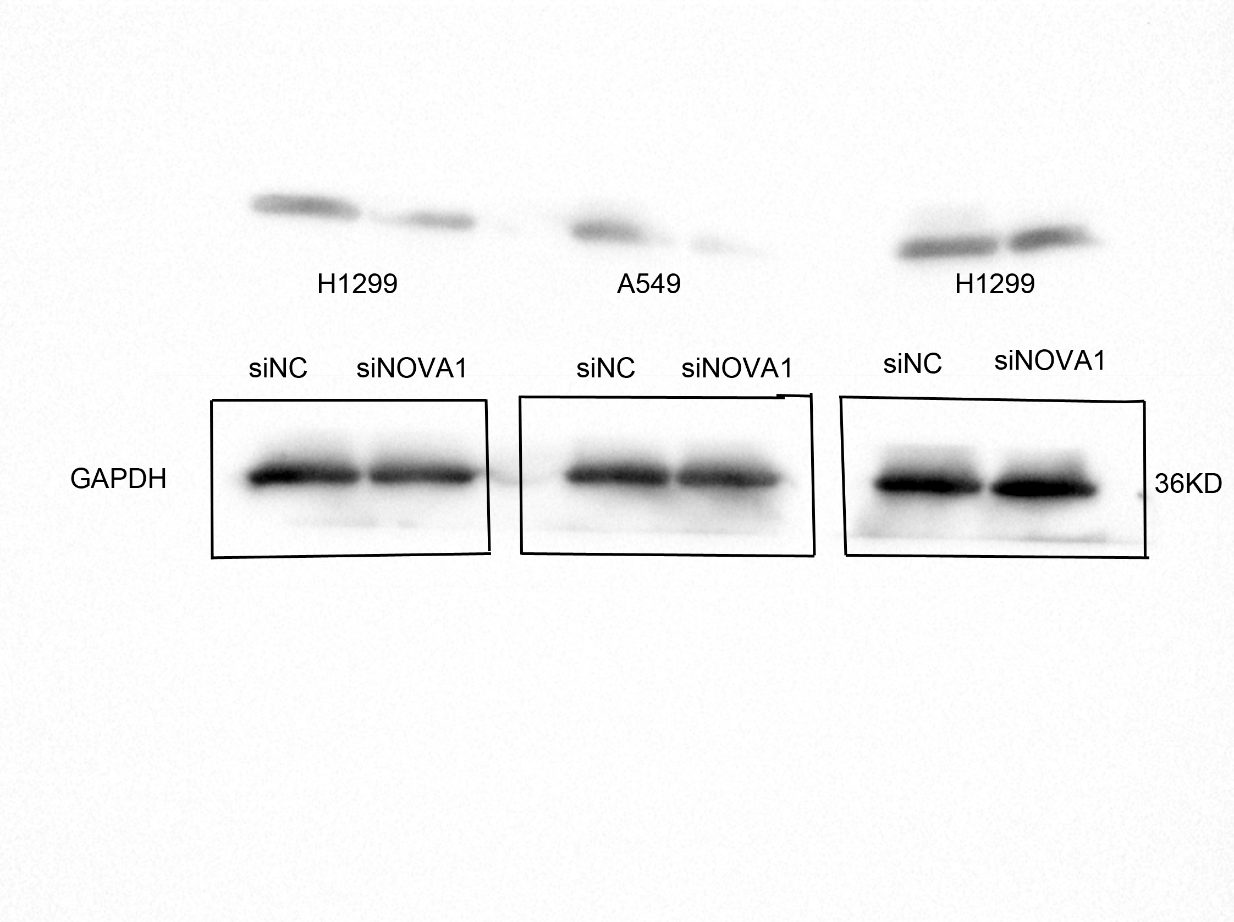

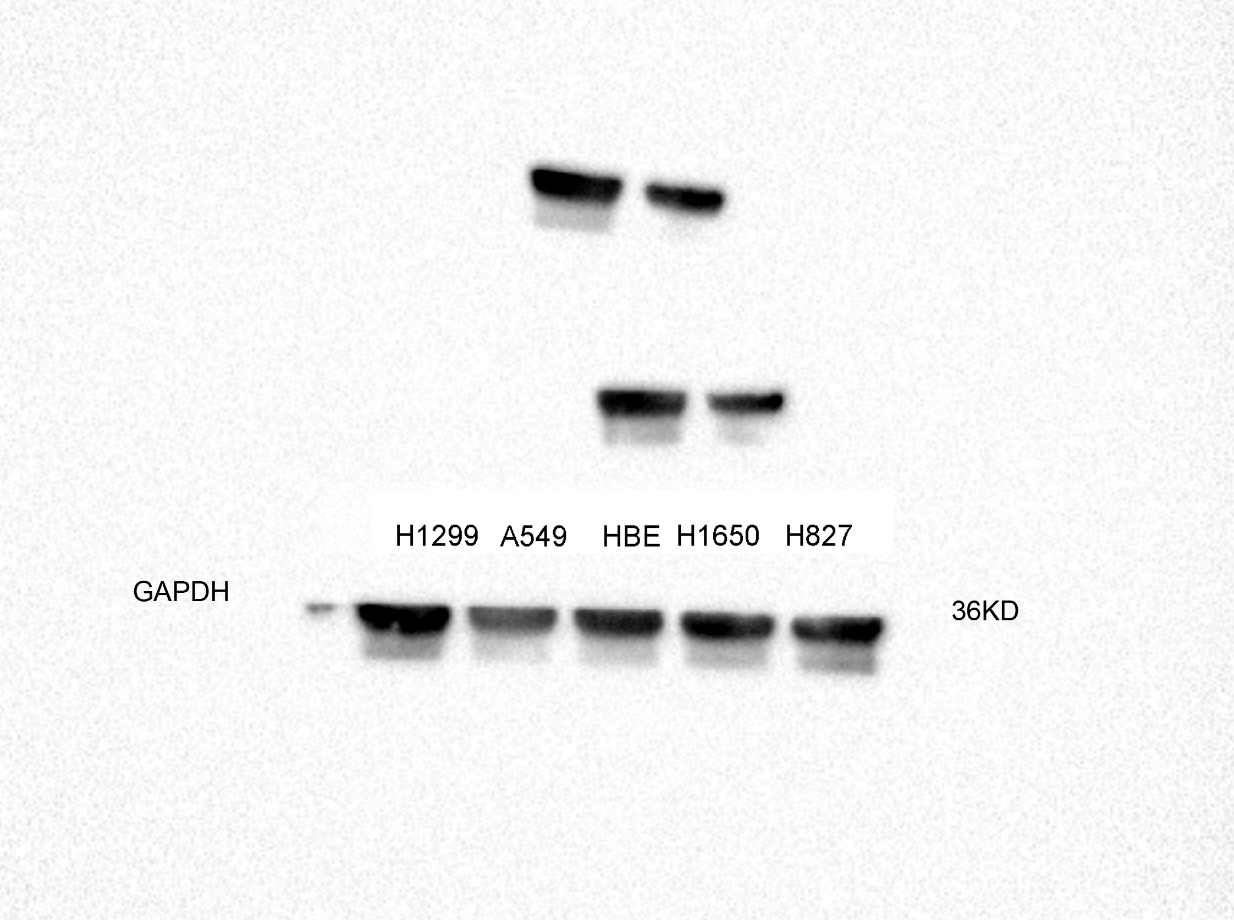

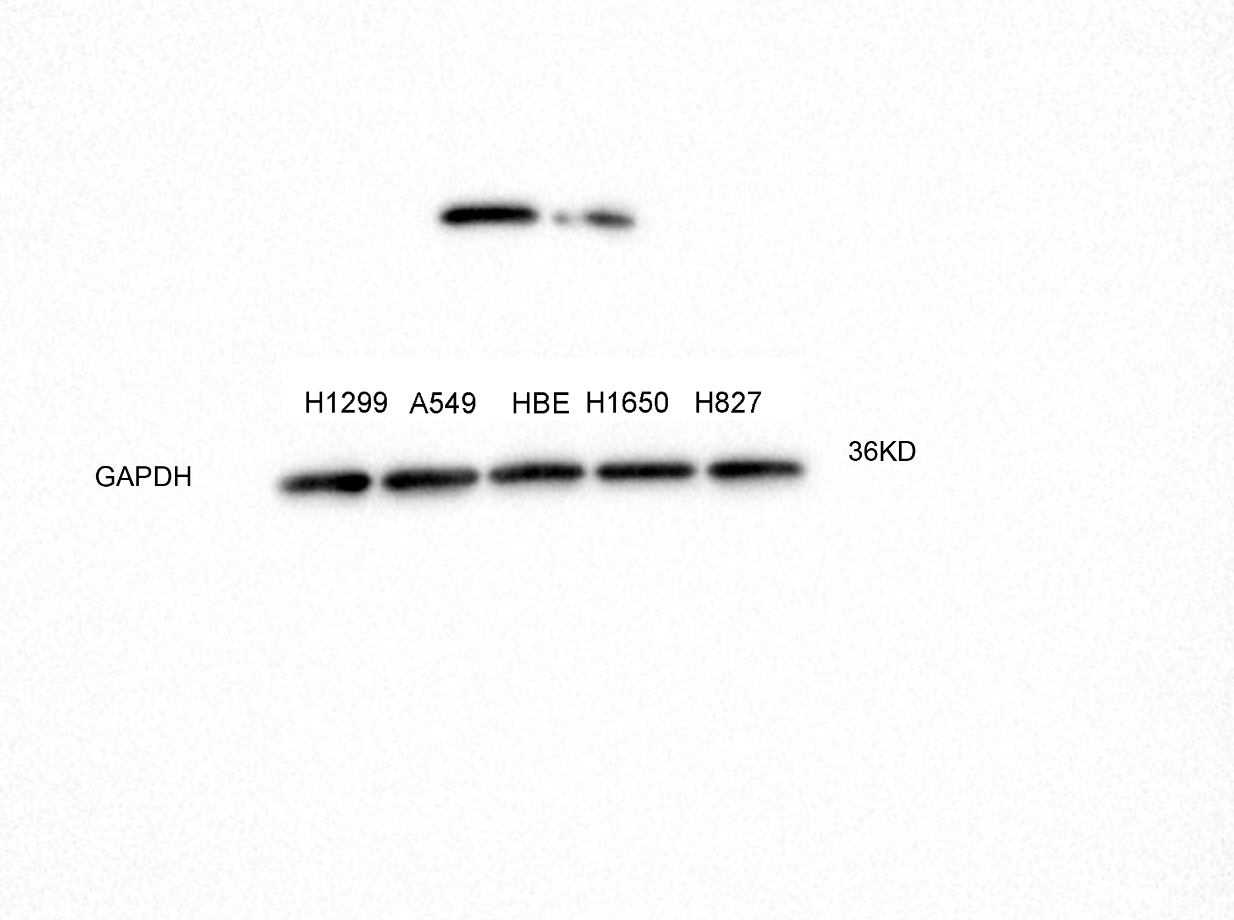


**Fuller-length, original, unprocessed blot  and replicate blots of b-catenin**

**
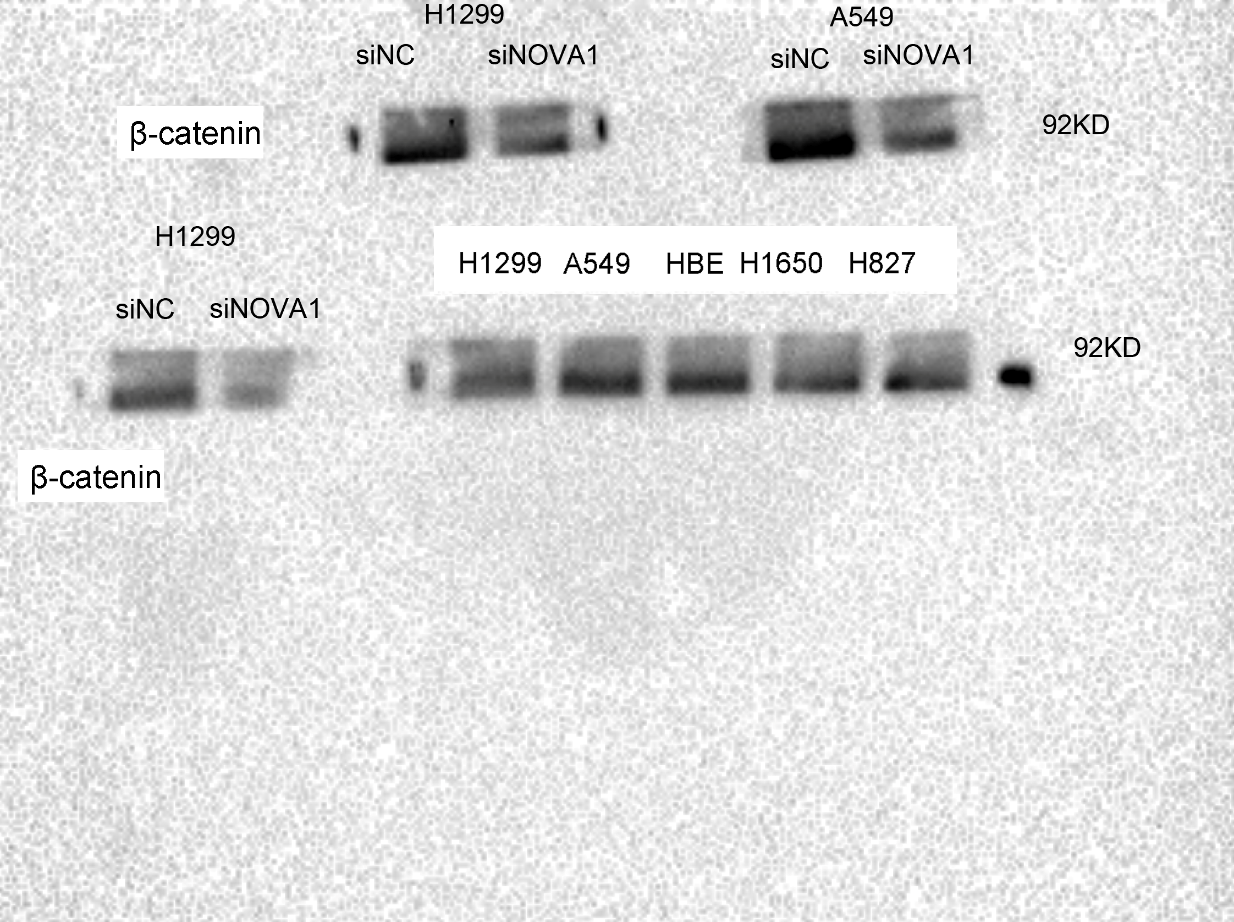

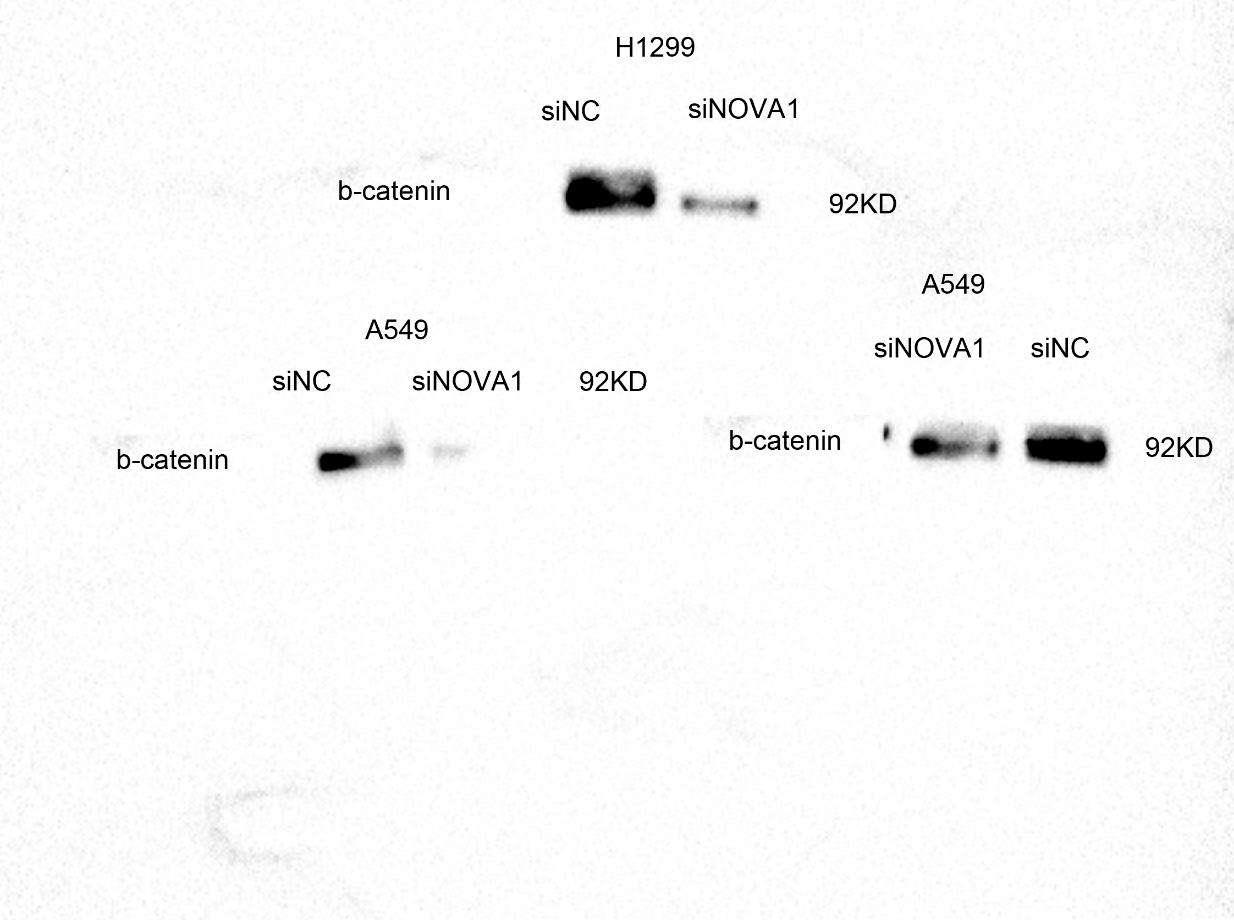
**


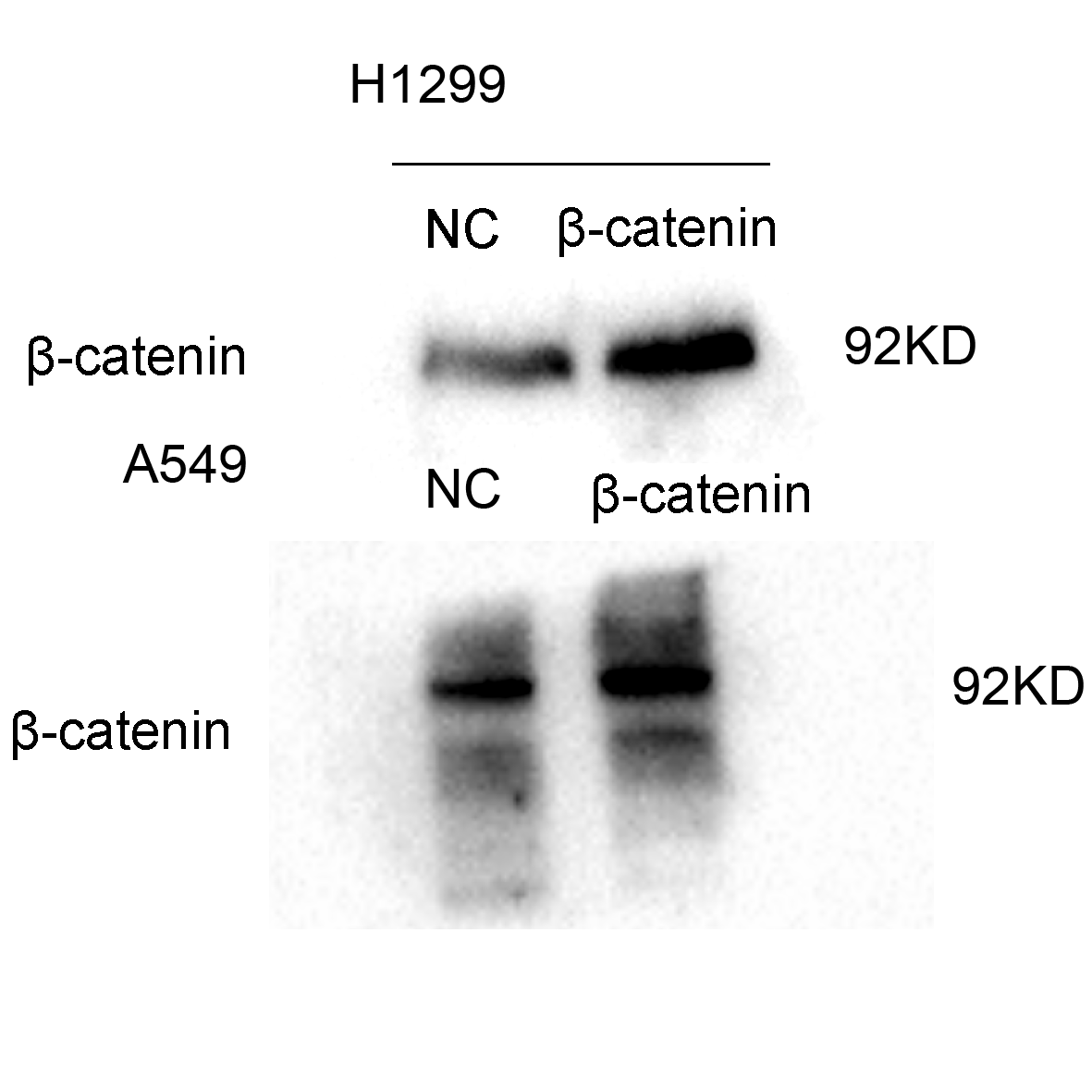


**Fuller-length, original, unprocessed blot  of Active b-catenin**


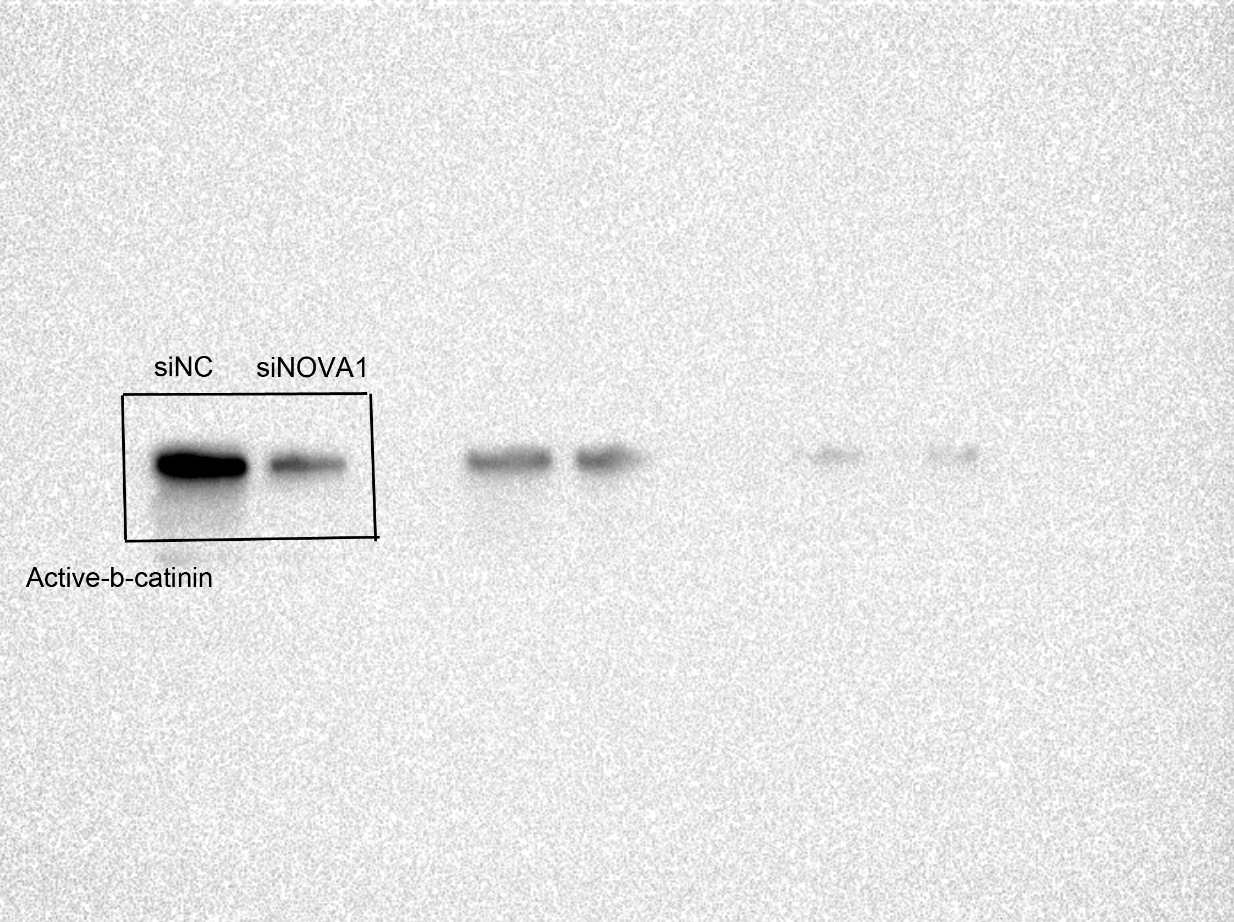


**Fuller-length, original, unprocessed blot  of CyclinD1**


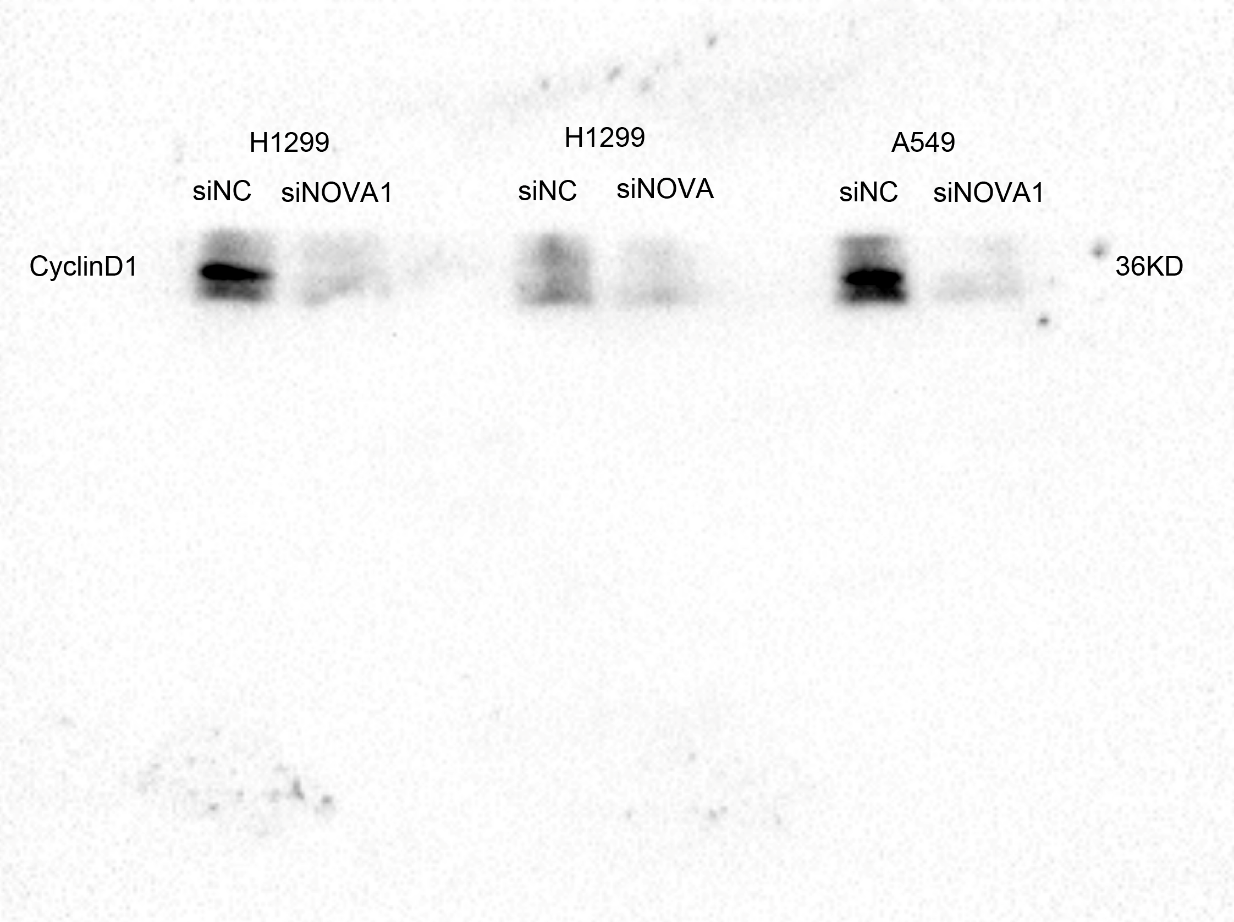


**Replicate blots of Cyclin D1 and Cyclin B**


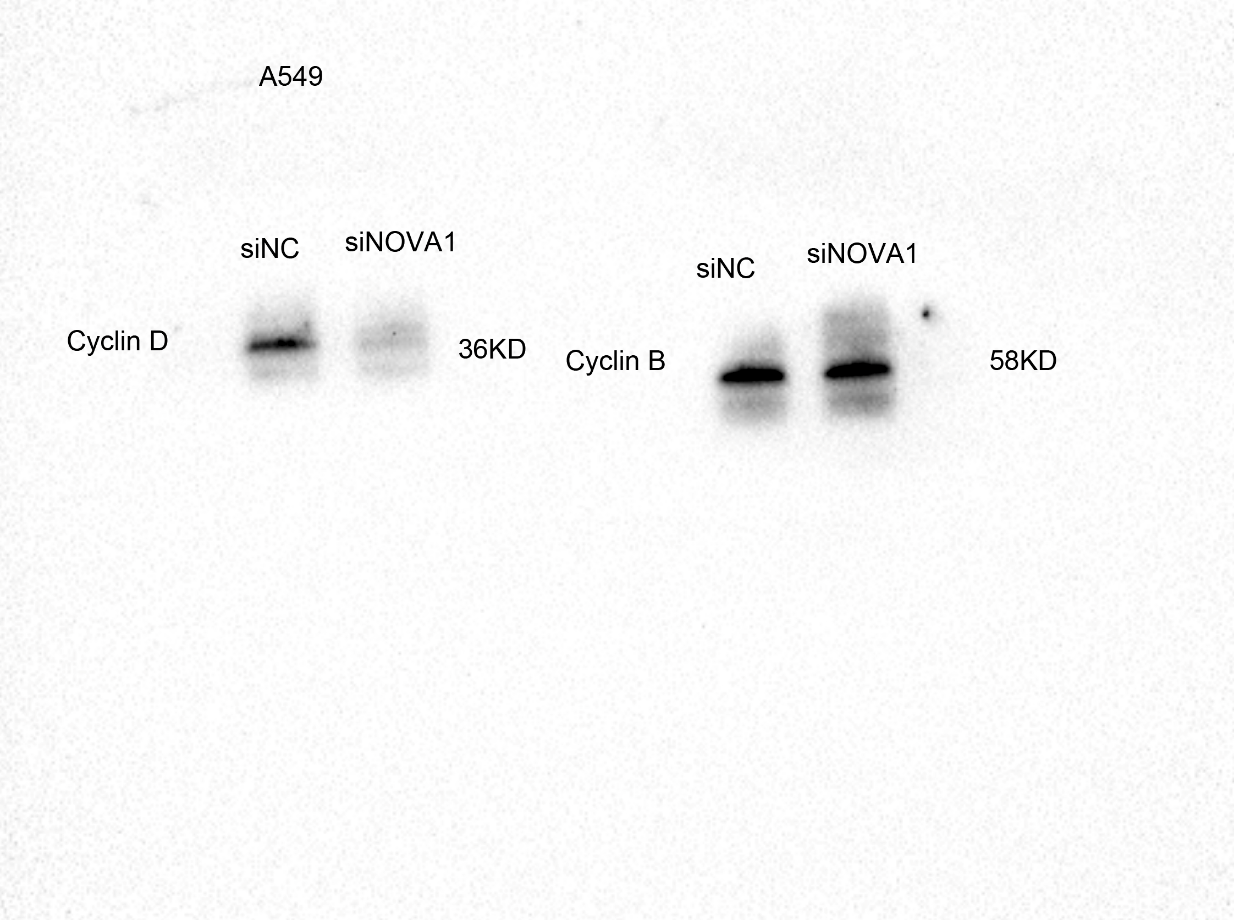

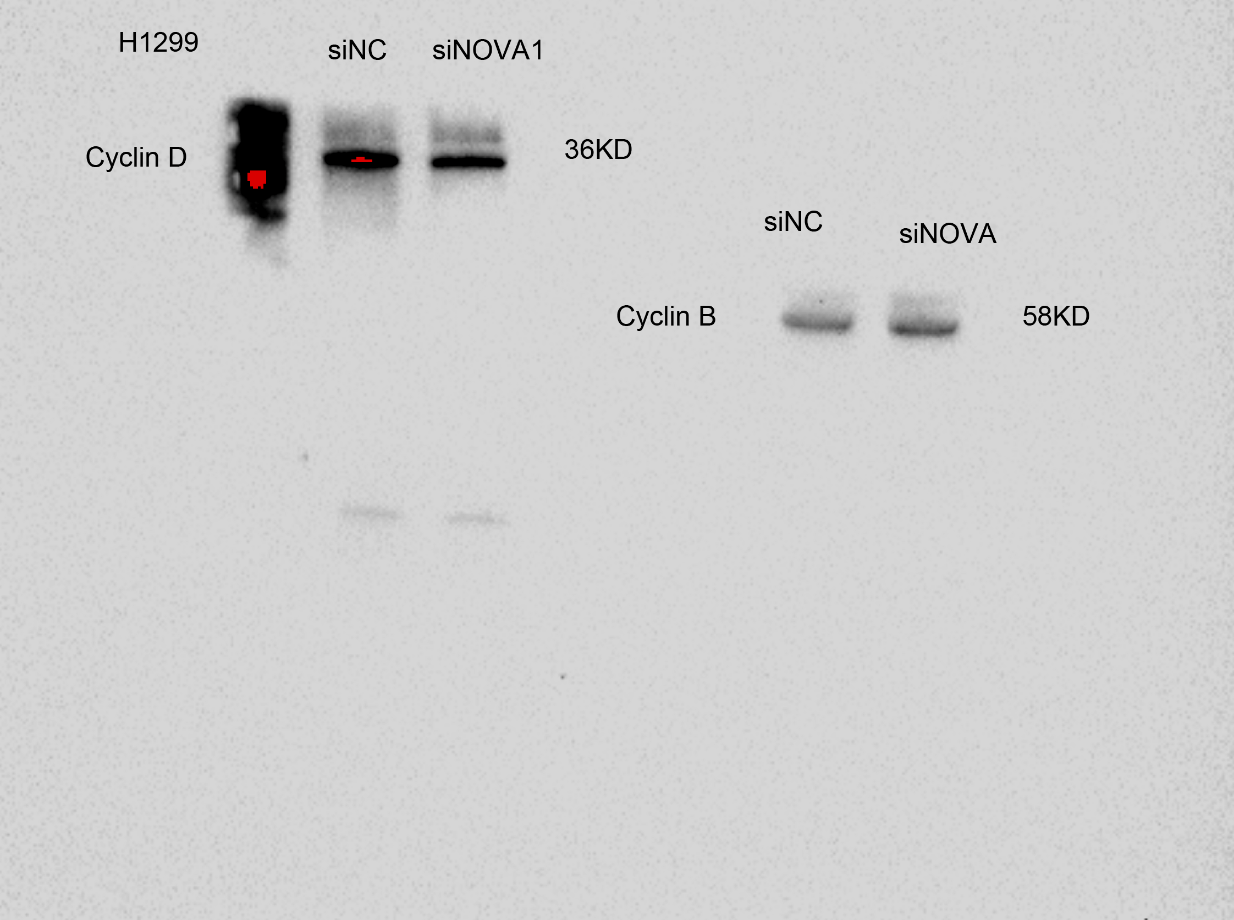


**Fuller-length, original, unprocessed blot  of**

**Cyclin B**


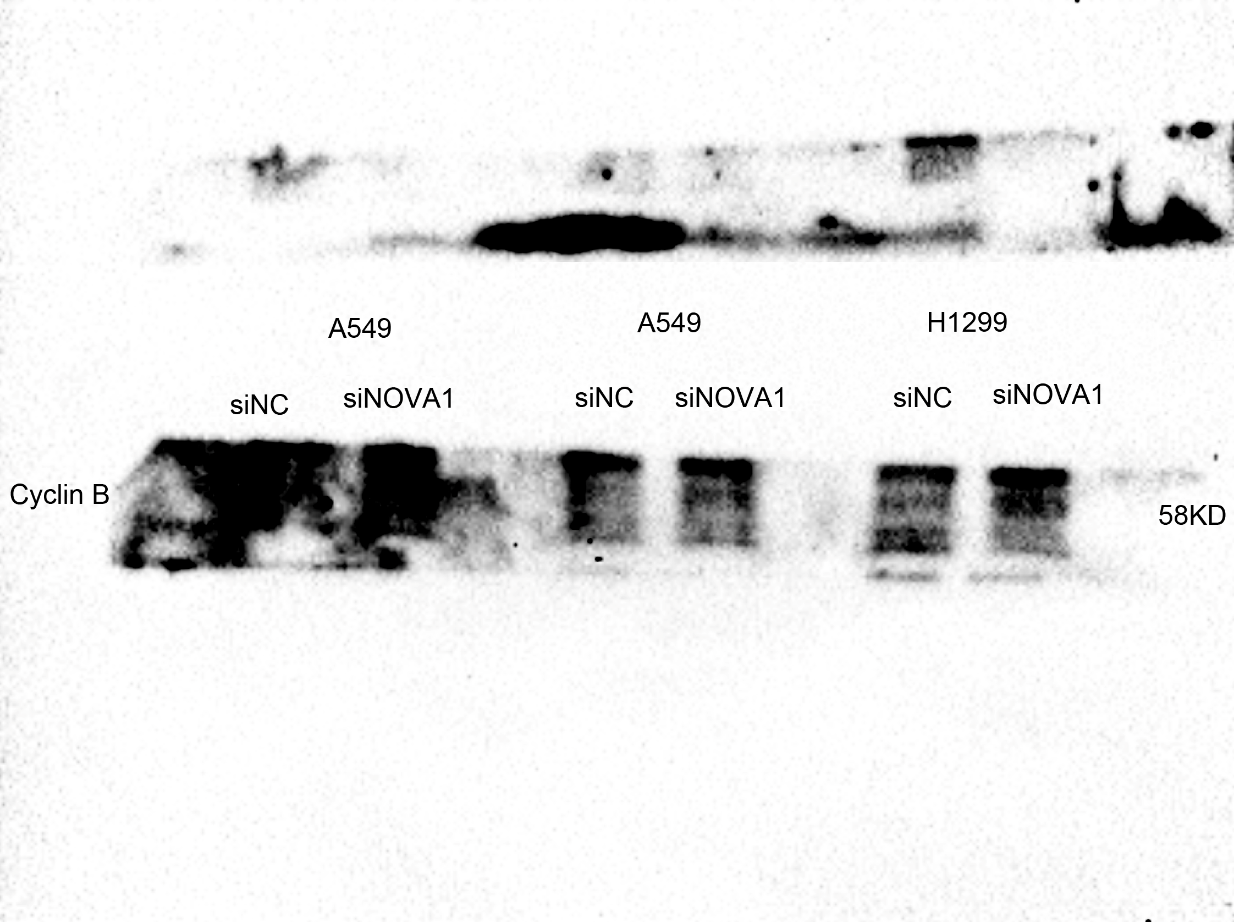


**Fuller-length, original, unprocessed blot  and replicate blots of MMP-2**

**
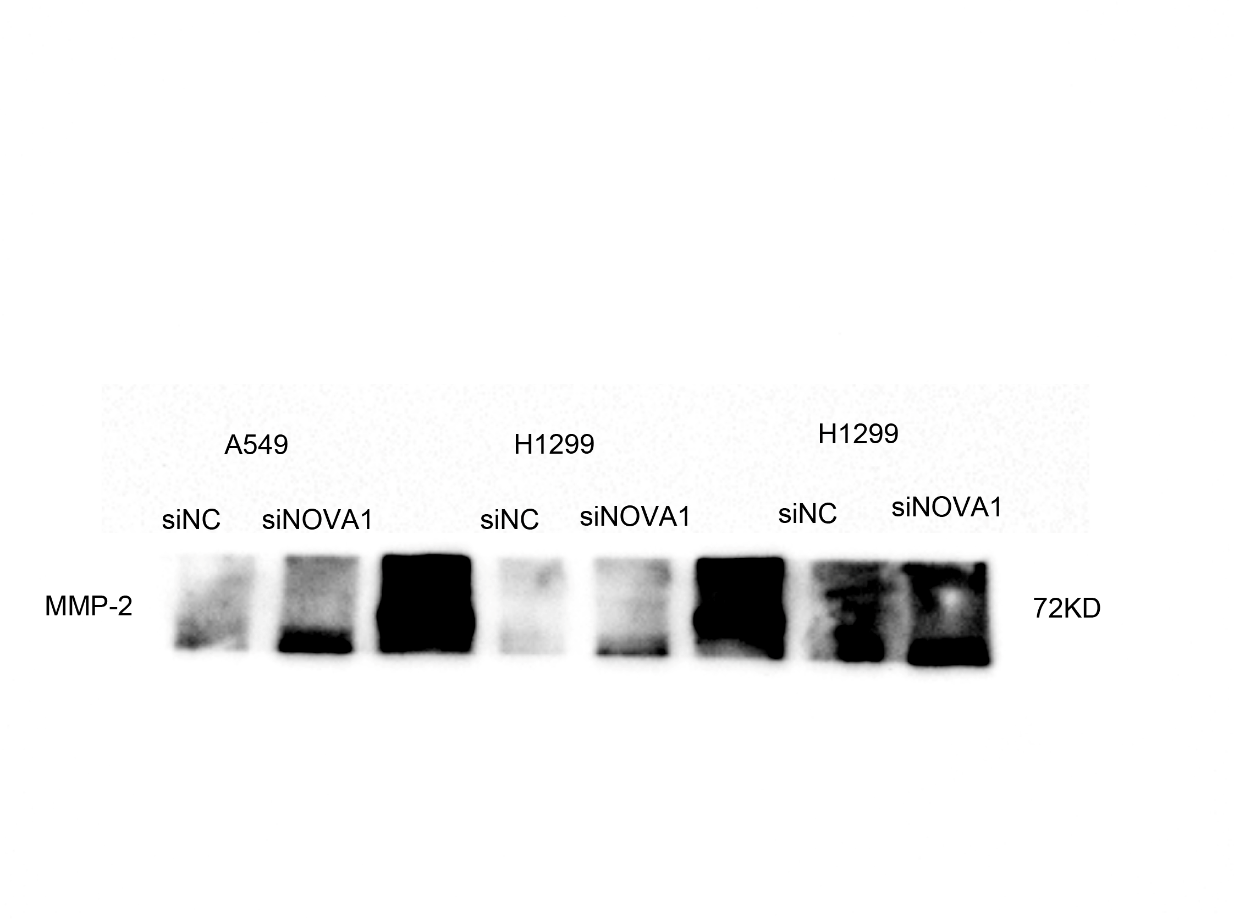
**

**
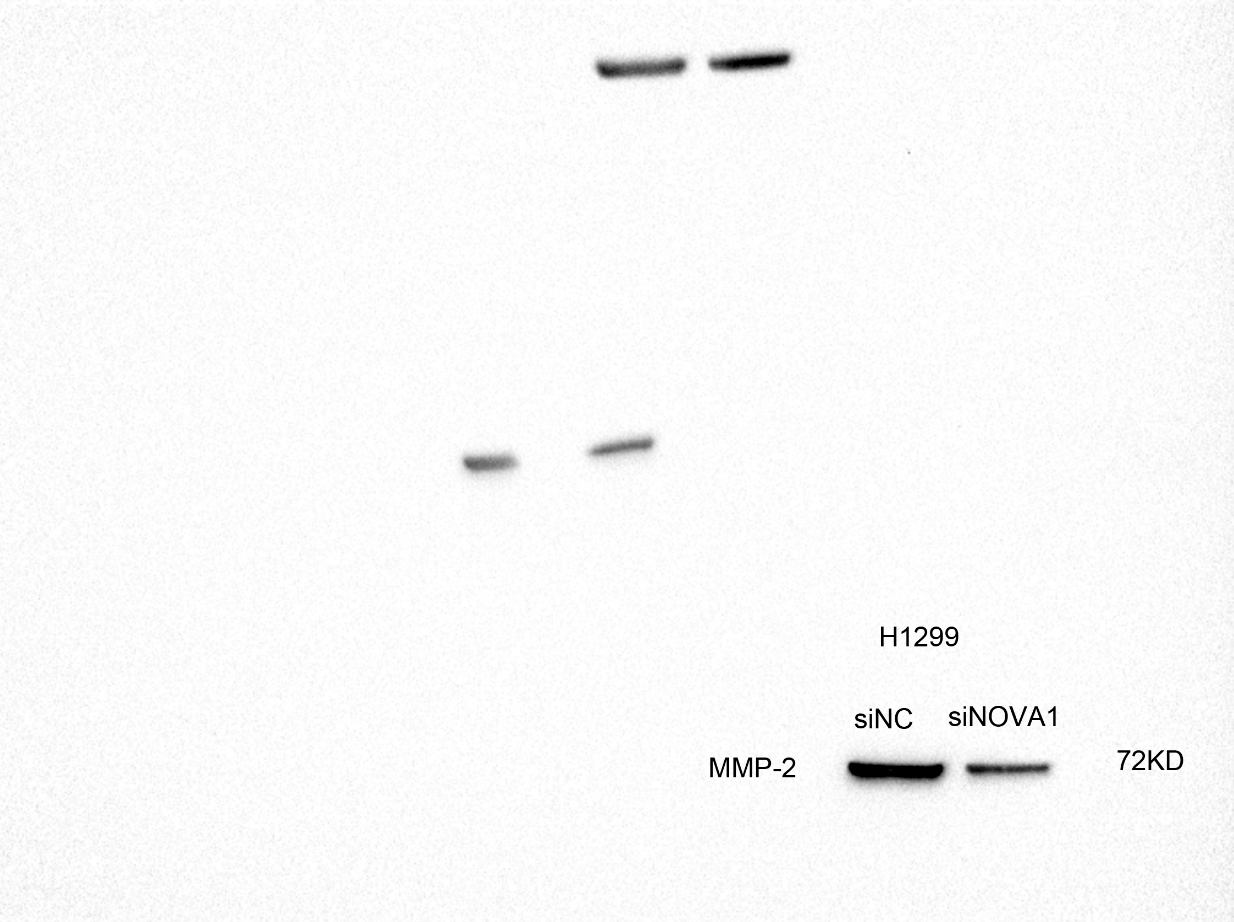

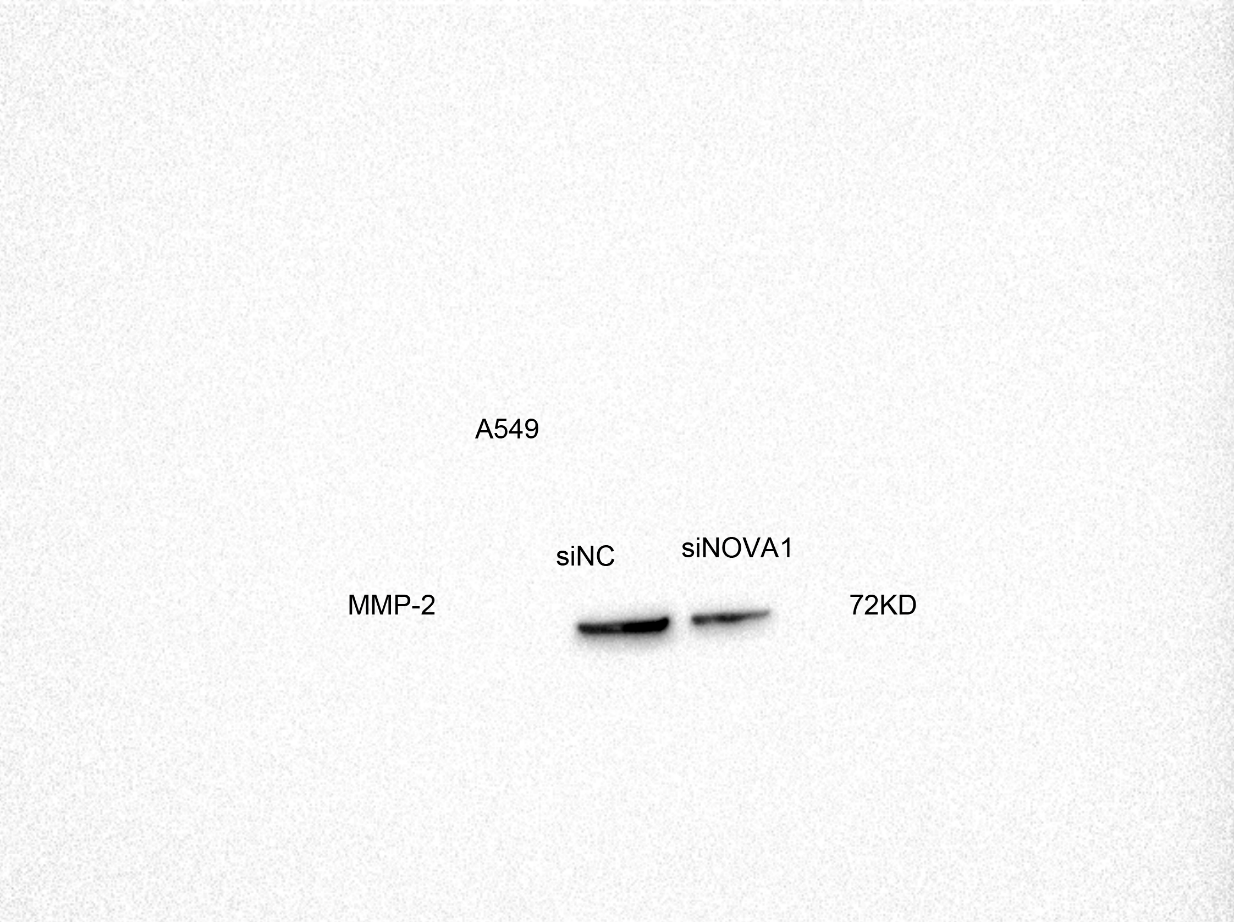

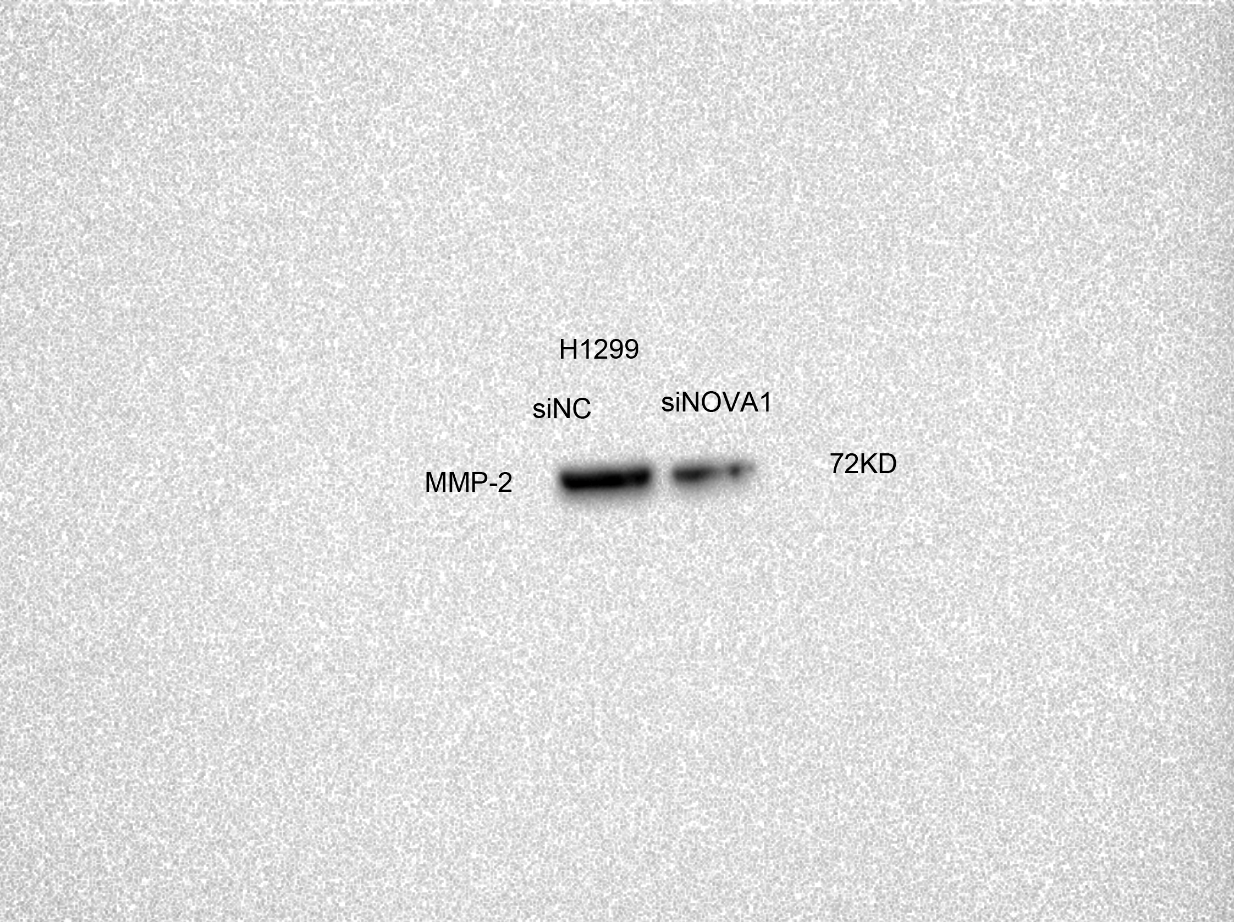
**

**Fuller-length, original, unprocessed blot  and replicate blots of MMP-7**

**
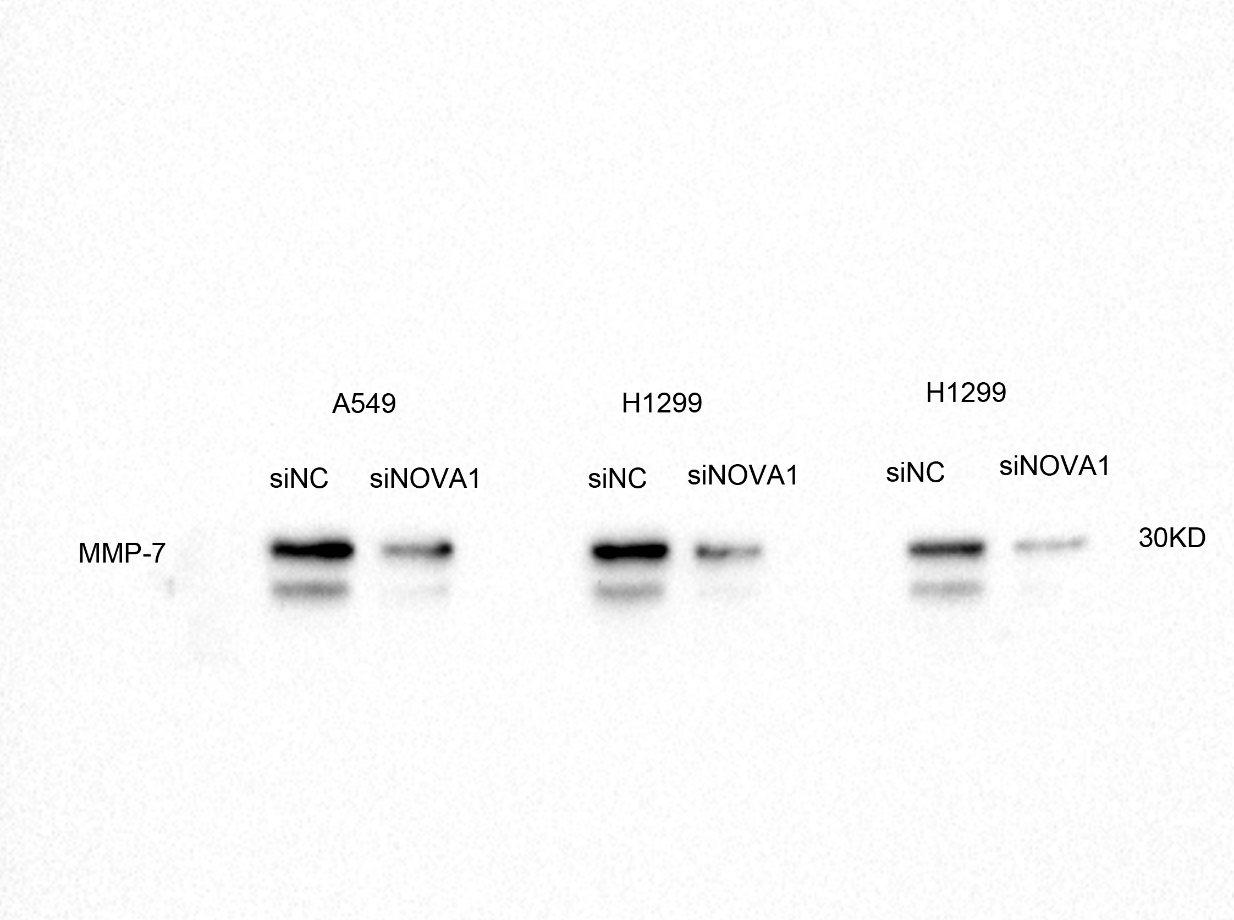
**


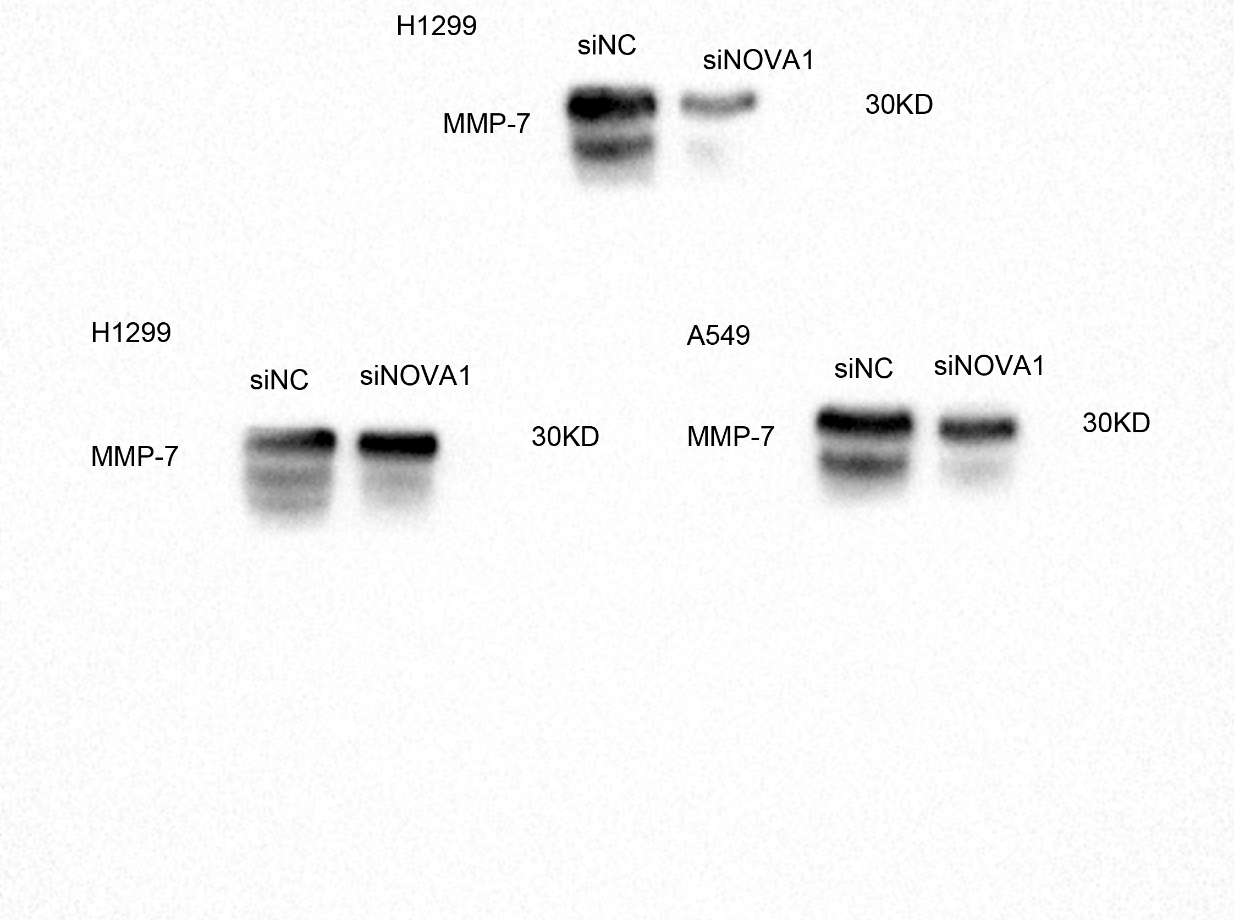

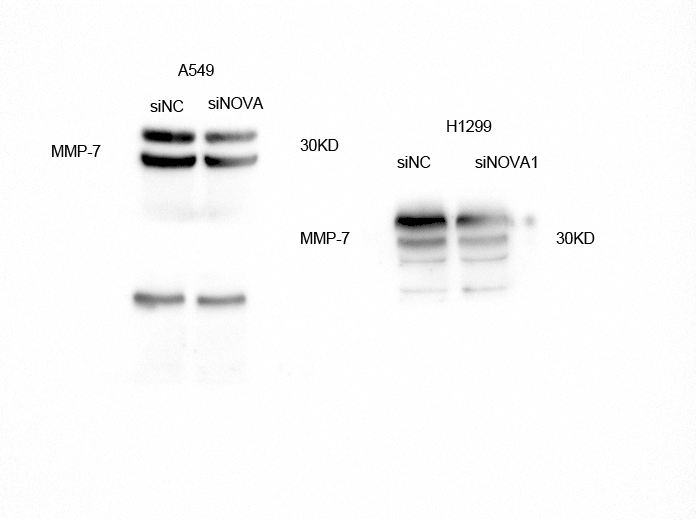


**Fuller-length, original, unprocessed blot  of E-ca**

**
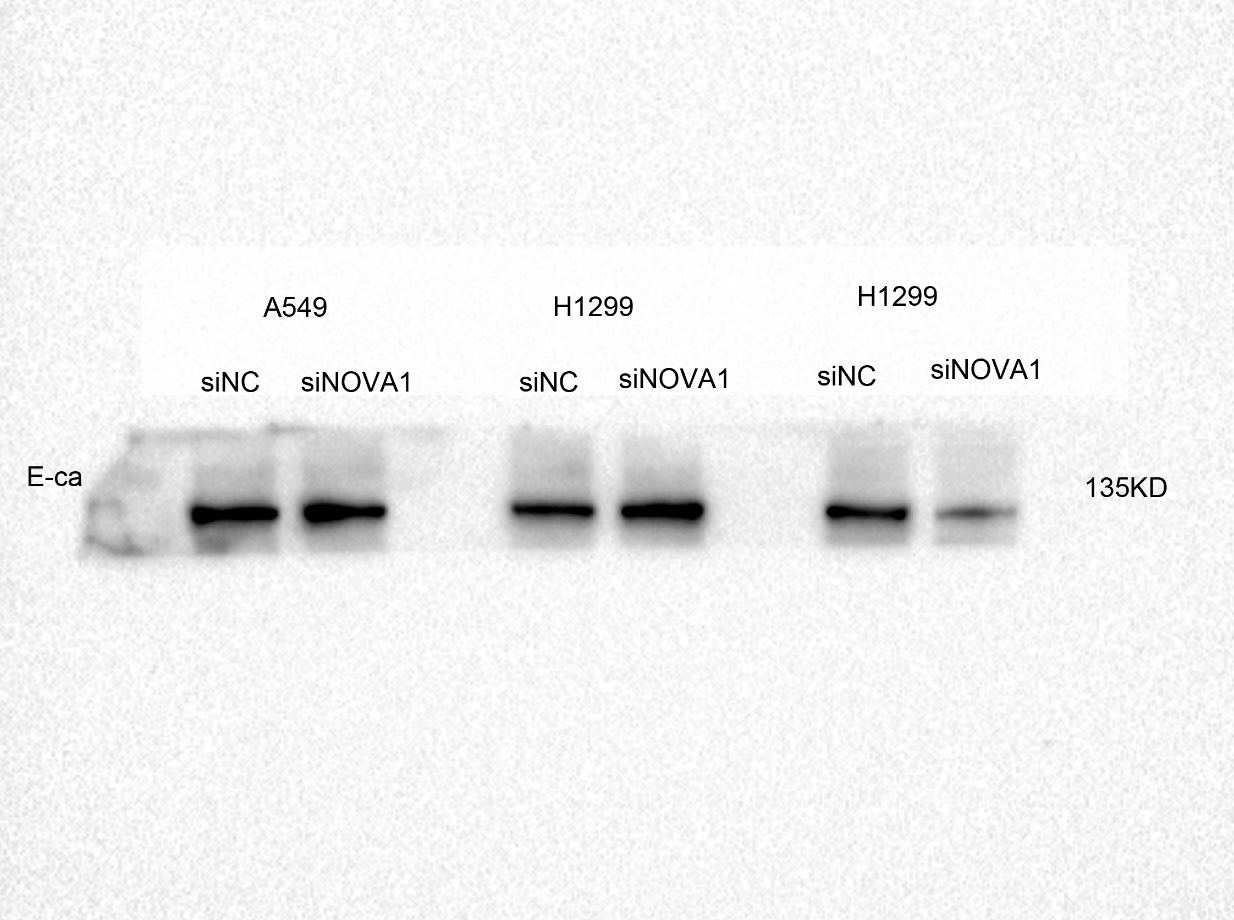
**
